# A gene expression atlas of a juvenile nervous system

**DOI:** 10.1101/2025.11.21.689793

**Published:** 2025-11-22

**Authors:** Seth R. Taylor, Claire Olson, Lidia Ripoll-Sanchez, Giulio Valperga, Rebecca McWhirter, S. Talmage Barney, Alexander Atkinson, Sidharth Goel, Alexis Weinreb, Andrew Hardin, Alexis Rolfson, Jacob Pattee, G. Robert Aguilar, Daniel M. Merritt, Matthew Eroglu, Maryam Majeed, Ethan Grundvig, Ethan Child, Isabel Beets, Petra E. Vértes, William R. Schafer, Erdem Varol, Marc Hammarlund, Oliver Hobert, David M. Miller

**Affiliations:** 1 Department of Cell and Developmental Biology, Vanderbilt University School of Medicine, Nashville, TN, 37240, USA; 2 Department of Cell Biology and Physiology, Brigham Young University, Provo, UT, USA; 3 Neuroscience Center, Brigham Young University, Provo, UT, USA; 4 Neurobiology Division, MRC Laboratory of Molecular Biology, Cambridge, UK; 5 Department of Psychiatry, Cambridge University, Cambridge, UK; 6 Department of Biological Sciences, Columbia University, New York, NY, USA; 7 Howard Hughes Medical Institute, Columbia University, New York, NY, USA; 8 Department of Biology, Brigham Young University, Provo, UT, USA; 9 Department of Computer Science & Engineering, Tandon School of Engineering, New York University, New York, NY, USA; 10 Department of Neuroscience, Yale School of Medicine, New Haven, CT, USA; 11 Department of Genetics, Yale School of Medicine, New Haven, CT, USA; 12 Current address: Allen Brain Institute, Seattle, WA, USA; 13 Department of Biology, KU Leuven, Leuven, Belgium; 14 Neuroscience Institute, Grossman School of Medicine, New York University, New York, NY, USA; 15 Program in Neuroscience, Vanderbilt University, Nashville, TN, USA

## Abstract

Although the fundamental architecture of metazoan nervous systems is typically established in the embryo, substantial numbers of neurons are added during post-natal development while existing neurons expand in size, refine connectivity, and undergo additional differentiation. To reveal the underlying molecular determinants of post-embryonic neurogenesis and maturation, we have produced gene expression profiles of all neuron types and their progenitors in the first larval stage (L1) of *C. elegans*. Comparisons of the L1 profile to the embryo and to the later L4 larval stage identified thousands of differentially expressed genes across individual neurons throughout the nervous system. Key neuropeptide signaling networks, for example, are remodeled during larval development. Gene regulatory network analysis revealed potential transcription factors driving the temporal changes in gene expression across the nervous system, including a broad role for the heterochronic gene *lin-14*. We utilized available connectomic data of juvenile animals in combination with our neuron-specific atlas to identify potential molecular determinants of membrane contact and synaptic connectivity. These expression data are available through a user-friendly interface at CeNGEN.org for independent investigations of the maturation, connectivity and function of a developing nervous system.

## INTRODUCTION

Neural circuits are generated by defined patterns of cell division or lineages giving rise to post-mitotic neurons that adopt distinctive morphologies and synaptic connections. In mammals, including humans, most neurons in the adult nervous system are generated during embryonic development^1,2^. Mature circuits are defined by the addition of post-natal neurons to networks initially established in the embryo. Post-mitotic neurons may also differentiate during development to modify circuit architecture and physiology. Gene expression, regulated for neuron type and across development, orchestrates the overall neurogenic program and thus embodies the genetic blueprint for the brain.

The *C. elegans* nervous system provides an attractive model for studies of neurogenic pathways. Over two-thirds of the neurons (222/302) in the adult hermaphrodite nervous system are generated in the embryo^3,4^. Most additional neurons are added during the first (L1) larval stage^4^. Neurons arise from a defined set of progenitors in the L1 and undergo post-mitotic differentiation as they are incorporated into existing circuits. The differentiated state of embryonically derived neurons is also modified during larval development. Notable changes include the dramatic expansion of cell volume, extension of neuronal processes and remodeling of synaptic contacts^5–11^. These anatomical changes are accompanied by marked differences in locomotory and sensory systems that likely depend on dynamic gene expression during larval maturation^12–15^.

In previous work, we produced a gene expression atlas for every neuron type in the mature *C. elegans* nervous system at the late larval L4 stage, a time by which the animal has reached almost full maturity^16^. Here we describe single cell RNA-seq (scRNA-seq) profiles of all *C. elegans* neurons in the first (L1) larval stage. Unlike previous early larval stage scRNA datasets that featured only portions of the nervous system^17–19^, our data encompass the entire L1 nervous system. In addition, we captured neuronal lineages of cell divisions in the L1 including all progenitors of post-embryonic ventral cord motor neurons. With these panoramic profiles in hand, we could undertake a comprehensive analysis of developmentally expressed genes for neurogenesis and differentiation across the entire nervous system. To identify genes that are differentially expressed in post-mitotic neurons during development, we compared profiles of each neuron at the L1 stage to its corresponding state in the embryo and in the L4. Our approach identified thousands of genes that are differentially expressed across this developmental axis, including ribosomal proteins, neuropeptides and neuropeptide receptors. Using these findings, we employed gene regulatory network analysis to identify a wide range of transcription factors that may orchestrate dynamic gene expression with development. We identified neuropeptide signaling networks at the L1 stage and discovered that these networks are remodeled as larval development progresses and new neuropeptide-dependent behaviors emerge^12,20^. We compared gene expression profiles with connectomic and protein-protein interaction data to identify cell adhesion molecules associated with specific neuronal circuits. Altogether, our data provide a rich resource for investigating the maturation of individual neurons and their progenitors for an entire nervous system.

## RESULTS

### A comprehensive gene expression profile of L1-stage neurons.

**Figure 1.**
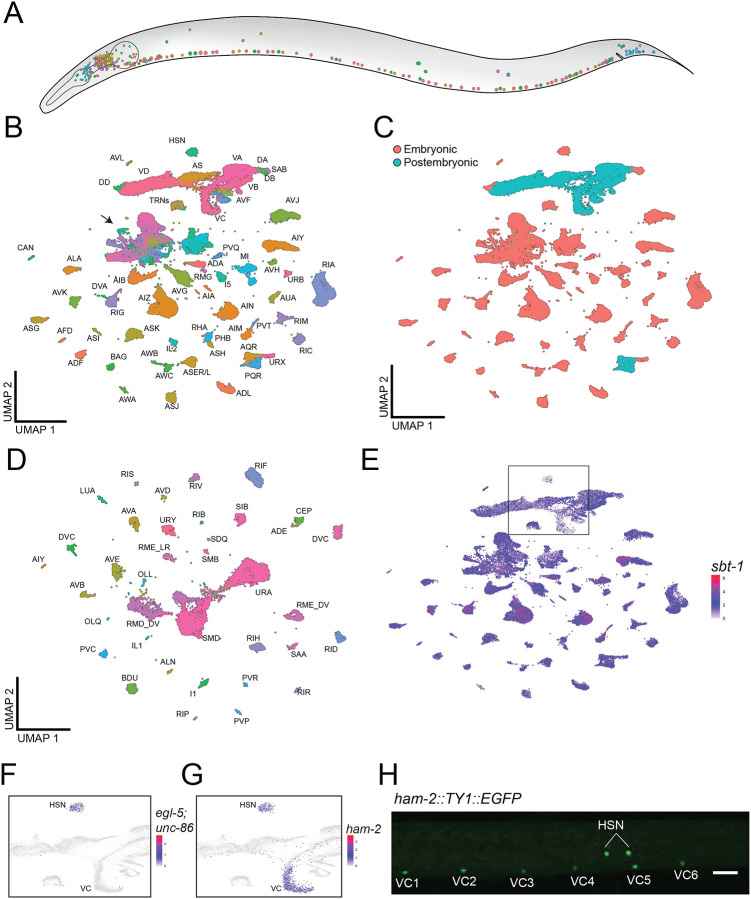
Single-cell RNA sequencing profiles of the L1 hermaphrodite nervous system A) Neurons in the *C. elegans* L1 larval nervous system. B) UMAP projection of 89,073 L1 neurons. Arrow denotes cell groups selected for sub-UMAP shown in panel D. C) UMAP projection of L1 nervous system colored by the generation time (embryonic or post-embryonic) of the neuron class. The majority of post-embryonic neurons grouped together. D) Sub-UMAP of the cluster denoted in panel B (arrow) shows clear separation of neuronal classes. E) UMAP as in B colored by expression of the post-mitotic neuron marker *sbt-1*. The two clusters with low *sbt-1* expression within the boxed region correspond to HSN and VC. F) Enlargement of the boxed region in E, colored by co-expression of *egl-5* and *unc-86*, which uniquely marks HSN. G) Enlargement of the boxed region in E, colored by expression of the transcription factor *ham-2*, which is restricted to HSN and VC. H) Confocal micrograph of a *ham-2* fosmid reporter in late L1 larvae shows expression in VC neurons in the ventral cord and in the HSN neuron pair. Scale bar = 10 μm.

We used 10X Genomics single-cell RNA sequencing (scRNA-Seq) to profile the mid to late first larval stage (L1) nervous system (Figure 1A)^21^. The nervous system of first stage (L1) larvae includes 1) 222 embryonically derived neurons^3,4^, and 2) 80 newly born, postembryonically-generated neurons, which arise from cell divisions in mid to late L1^4^. We used a mesh-filtration protocol to generate large synchronous populations of L1 animals as an alternative to synchronizing by L1 arrest, which alters larval gene expression^22^ (Supplemental Figure 1B). We used established methods to dissociate L1 larval cells for FACS enrichment of neurons from fluorescent marker strains for scRNA-Seq (Supplemental Figure 1A, Supplemental Tables 1, 2)^16,23^. After filtering scRNA-Seq data based on quality control metrics and removal of doublets and damaged cells (see Methods), we generated profiles of 161,072 single cells from 16 experiments (Supplemental Figure 1C, Supplemental Table 3). We detected a median of 841 unique molecule identifiers (UMIs) per cell and a median of 373 genes per cell.

Using previously published datasets from embryos, larval stages (L2, L4), and adults as references^16,17,24,25^, we assigned anatomical identities to 93.5% of the cells in the dataset. In addition to neurons, we annotated cells belonging to postembryonic progenitor lineages and several terminally differentiated non-neuronal cell classes (Supplemental Figure 1C–E, Supplemental Table 4). Altogether, we identified 89,073 post-mitotic neurons in our dataset with a median of 701 UMIs and 326 genes detected per cell (Figure 1B).

### Molecularly distinct neuronal subclasses arise in the first larval stage.

The 302 neurons of the *C. elegans* adult hermaphrodite have been grouped into 118 distinct classes based on morphology and connectivity^26,27^. Ninety-six of the 118 anatomically-defined classes arise from cell divisions in the embryo^3^, six classes are generated from cell divisions in the middle of the first (L1) larval stage (AQR, AVM, PQR, PVM, PVW, SDQ), 11 classes arise in late L1 (AS, AVF, DVB, PDB, PHC, PLN, RMH, VA, VB, VC, VD), and the remaining five classes are generated (PDE, PVD, PVN, RMF) or differentiate (PDA) in the second larval stage (L2) or later^4^. Our L1 dataset contains transcriptomes of all hermaphrodite neuron classes at mid L1 (Figure 1B). Additionally, we captured nearly all the classes born in late L1 (9/11 classes; DVB and PDB were not identified).

Several anatomically-defined classes of neurons have been further separated into molecularly distinct subclasses in the late larval and adult nervous system^16,27,28^. In some cases, the developmental onset of these distinctions has been established. For example, AWC and ASE sensory neurons show clear left/right asymmetries in the embryo^24,29,30^ and we detected these subclasses for ASE (ASER/ASEL) and AWC (AWC^ON^/AWC^OFF^) neurons in the L1 larva. Our results suggest that other subclasses arise at least as early as the L1 stage. We detected subclasses for the IL2 sensory neurons (IL2 DV/IL2 LR), and the RME (RME DV/RME LR) and RMD (RMD DV/RMD LR) motor neurons in the L1, as previously identified in L4 larval animals (Supplemental Figure 2A)^16,25,28^. We detected two subclasses for both the DA and DB motor neurons in the L1 data set, with the most anteriorly located neurons of each class, DA1 and DB2, adopting distinguishable gene expression profiles (Supplemental Table 4, Supplemental Figure 2B–F), consistent with recent scRNA-Seq data from adult animals^28^. We also detected subclasses for the postembryonic VA, VB, and VD ventral cord motor neuron classes. These results are discussed in detail below, in the section featuring postembryonic motor neurons derived from the P cell lineages (Figure 3). We did not detect DA9 and VA12 motor neuron subclasses in the L1 data set, possibly due to low sampling or a later time of maturation for the most posteriorly located members of the VA motor neuron classes^4^.

Two classes of hermaphrodite-specific neurons, the HSN and VC neurons, are generated early in development but do not fully differentiate until late L4/adulthood. The HSN neuron pair is generated in the embryo, whereas the six VC neurons are born at the end of the first larval stage^3,4^. We identified distinct clusters corresponding to HSN and VC neurons. Consistent with the delayed differentiation of HSN and VC neurons, the HSN and VC clusters show significantly weaker expression of pan-neuronal marker genes in comparison to all other neurons at the L1 stage (Supplemental Figure 3A). We identified HSN neurons based on the co-expression of *unc-86*, *egl-5,* and *ham-2* (Figure 1F–H)^31,32^. VC neurons were identified by co-expression of *lin-39, unc-62, ham-2* and *ceh-6* (Figure 1H, Supplemental Figure 3C–E)^33,34^. In total, we identified 126 transcriptionally distinct neuron classes in L1 larvae.

### Temporally regulated genes distinguish progenitors from post-mitotic neurons

The 17 classes of postembryonically derived neurons generated in the L1 derive from a limited number of progenitor lineages. The G1 neuroblast in the head gives rise to RMH, the Q lineage yields six neurons in the midbody (AVM, AQR, PVM, PQR, SDQL/R), the P lineage produces seven neuron classes along the anterior to posterior axis of the ventral nerve cord (AVF, AS, PDB, VA, VB, VC, VD), the T lineage generates three sets of neurons in the tail (PVW, PHC, and PLN), and the tail K cell gives rise to DVB^4^. We captured cell-specific profiles of both progenitor cells and post-mitotic neurons from all these lineages (G1, Q, P and T) except for the K cell lineage. These data provide a valuable foundation for further investigation into neuron differentiation and will be described in the ensuing sections.

### Distinguishing progenitors and post-mitotic neurons in the Q lineage.

**Figure 2.**
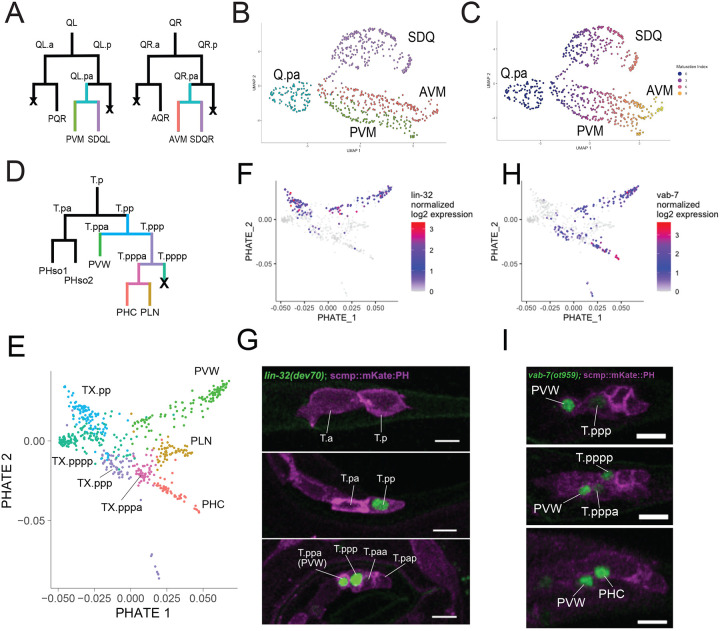
Neuronal progenitor cells in the Q and T lineages A) QL and QR lineages each giving rise to three neurons and two daughter cells that undergo apoptosis (X). B) Sub-UMAP of Q.pa and descendants, SDQ, AVM and PVM colored by neuron class. C) Sub-UMAP of the Q.pa lineages with post-mitotic neurons colored by maturation index. D) The T.p lineage produces two glial cells (phasmid sockets), three neurons (PVW, PHC, and PLN) and a cell death (X) on each side of the worm. E) PHATE plot showing annotations of T.pp and descendants. F) PHATE plot showing expression of *lin-32* in the T.pp lineages. G) Confocal micrographs of an endogenous *lin-32* reporter and a membrane bound mKate expressed in the T lineage by a seam cell promoter. H) PHATE plot showing expression of *vab-7* in PVW (T.ppa) and a subset of progenitor cells. I) Confocal micrographs showing endogenously tagged *vab-7* in combination with the membrane bound mKate in the T-lineage. Expression of *vab-7* begins in PVW (T.ppa), its sister T.ppp, and the T.ppp descendants, including PHC. All scale bars = 10 μm.

Neurons from the Q lineage are generated in the mid-L1 (Figure 2A), whereas neurons from the P and T lineages arise a few hours later, in the late L1^4^. Recent work used scRNAseq to profile the Q lineage, with preferential sampling of early progenitors^35^. As we sampled from mid to late L1, our dataset preferentially samples post-mitotic AQR and PQR neurons, the neuroblasts QL.pa/QR.pa, and the newly born AVM, PVM, and SDQ neurons. The UMAP embeddings for the QL.pa/QR.pa lineages match the pattern of known cell divisions. Two branches diverge from the progenitor population (marked by transcripts such as *cdk-1,* a known cell-cycle gene^36,37^), corresponding to SDQL/R and AVM/PVM (Figure 2B, Supplemental Figure 4A). Within the post-mitotic cells, the UMAP embeddings appear to separate cells by developmental age. For example, the POU transcription factor UNC-86 is detected in the SDQ neurons at birth, but disappears within hours^38^. In the scRNA-seq data, the SDQ cells closest to the SDQ progenitor cluster in the UMAP express the *unc-86* transcript, but this expression decreases until it is undetectable in SDQ cells furthest from the progenitor cluster (Supplemental Figure 4C). In the two postembryonically derived AVM and PVM touch neurons, expression of the cell-fate determining transcription factor *mec-3* is detected in all the cells. In contrast, *mec-3*-dependent touch neuron markers (e.g., *mec-17, mec-4*)^39^ are exclusively detected in cells further from the progenitor cluster (Supplemental Figure 4D–E).

Since the UMAP embeddings appeared to capture the developmental stage of individual cells, we assigned a ‘maturation index’ (see Methods) based on the Euclidean distance of each post-mitotic neuron from the progenitor cluster. We then correlated gene expression with the maturation index to identify genes that vary in expression. Transcripts positively correlated with the maturation index reflect genes that are upregulated in differentiating post-mitotic neurons (e.g., *mec-17*) (Supplemental Figure 4F, H). Conversely, transcripts that are negatively correlated with the maturation index are downregulated after neurons are generated in the Q lineage. For instance, the zinc finger transcription factor *egl-46* is strongly expressed in the AVM/SDQR progenitor but not in the mature AVM neuron (Supplemental Figure 4G–H), a result consistent with the finding that *egl-46* regulates exit from the cell cycle in Q-derived neurons and neuronal differentiation in other neuron classes^40–44^.

Overall, we identified hundreds of genes that were significantly correlated with maturation in AVM, PVM, and SDQ (Supplemental Figure H-L, Supplemental Table 5). We noted many genes with either negative or positive correlations in all three neuron classes, including the cathepsin A carboxypeptidase *ctsa-1.1* and the stomatin ortholog *mec-2* (Supplemental Figure 4K). Other transcripts with positive correlations were unique to each neuron class at the L1 stage, such as the neuropeptides *flp-20, flp-8* and *flp-12* (Supplemental Figure 4L).

### Distinguishing progenitors and post-mitotic neurons in the T lineage

The left and right T cells (TL and TR) are the most posteriorly located seam cells at hatching. T cells give rise to epidermal cells, glial cells and neurons. In the late L1, the posterior daughters of TL and TR generate three tail neurons and two glial cells (Figure 2D)^4^. The T.p-derived neurons and some of their progenitors express the POU-domain transcription factor *unc-86*^38,45,46^ and the posterior Hox genes *php-3* and *nob-1*^38,45^. By using FACS to isolate cells marked with the *unc-86* promoter driving myristoylated-GFP (CX5974), we captured both progenitors and the newly generated neurons of the T.p lineage (Figure 2E; Supplemental Table 3). The PHATE dimensionality reduction method^47^ generated two-dimensional embeddings with three distinct branches corresponding to the three neuron classes PVW, PLN and PHC that arise from the T.p lineage (Figure 2E, Supplemental Figure 4M–N). We annotated multiple clusters as T.p lineage neuronal progenitors based on co-expression of *cdk-1*, *unc-86*, *nob-1* and *php-3* (Supplemental Figure 4M). Several transcription factors were expressed in specific subsets of progenitor cells, including the pro-neural ATOH1 bHLH transcription factor (TF) homolog *lin-32*. We validated this finding *in vivo* with an endogenously tagged *lin-32* GFP reporter that is initially expressed in T.pp, and later in its descendants, T.ppa (PVW) and T.ppp (Figure 2F–G).

The EVX1/2 TF homolog *vab-7* is expressed in PVW and in a distinct progenitor cluster that gives rise to PHC (Figure 2H). We confirmed expression of the VAB-7 protein in these lineages with an CRISPR/Cas9 engineered GFP-tagged *vab-7* reporter allele that is detected in PVW and its sister, the T.ppp neuroblast, and daughter cells, including PHC (Figure 2H–I). We used these combined results to annotate the T.pp lineage (Figure 2E). Additionally, we detected a distinct group of cells among T-lineage progenitors with strong *unc-86* and *egl-1* expression (data not shown). This cluster had few other strongly enriched genes beyond *egl-1*, and fewer genes and UMIs than other T-lineage cells. Given the role of *egl-1* in apoptosis^48,49^, we annotated this cluster as T.pppp (Figure 2E), which undergoes apoptosis during normal development.

Previous work demonstrated that *lin-32* regulates neuronal fate in several lineages^50–55^. Studies on *C. elegans* males have shown variable effects of *lin-32* loss on the T-lineage derived ray neurons^56,57^. To determine if *lin-32* influences neuronal fate in the hermaphrodite T-lineage, we combined the loss of function *lin-32(u282)* allele with a CRISPR/Cas9 engineered GFP-tagged *vab-7* reporter allele as a readout of PVW and PHC cell fate. Loss of *lin-32* dramatically reduced the number of VAB-7 expressing neurons in the tail (Supplemental Figure 4O), indicating that *lin-32* is required for neuronal development in the hermaphrodite T lineage.

### Distinguishing progenitors and post-mitotic neurons in the P lineage

**Figure 3.**
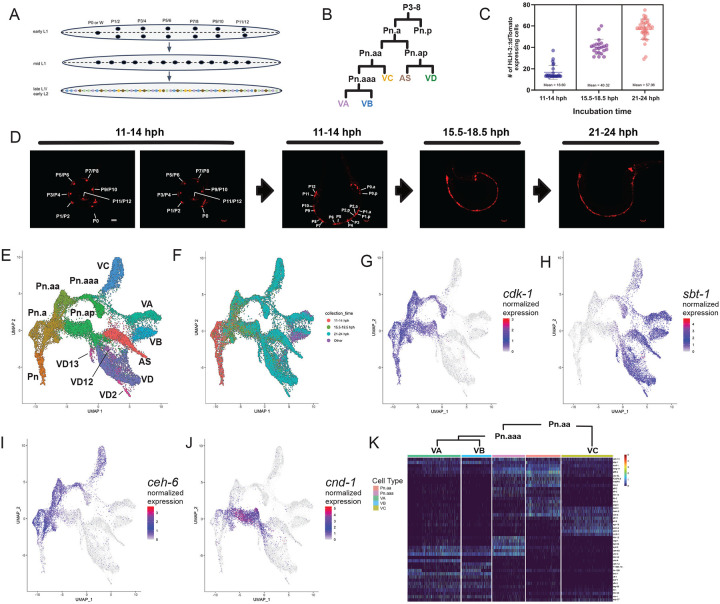
P-lineage derived progenitors and neurons (A) Diagram of P-cell migrations to the ventral cord and subsequent cell divisions during the L1 stage. (B) Diagram of the P3-P8 lineages, each of which produce five motor neurons. (C) Quantification of HLH-3::tdTomato expressing cells counted during each collection period (hph, hours post hatch). (D) Confocal micrographs of HLH-3::tdTomato expressing cells in the ventral cord during each collection period. The first two images featuring the bilateral array of P-cells before they migrate to the ventral cord, show two different Z-planes in the same individual. (E) Sub-UMAP of P1-P12 lineage derived progenitors and neurons colored by cell type, (F) colored by the time in which cells were collected, (G) colored by expression of the cell cycle marker *cdk-1*, (H) colored by expression of the post-mitotic neuron marker *sbt-1*, (I) colored by expression of the transcription factor *ceh-6*, and (J) colored by expression of the transcription factor *cnd-1*. (K) Heatmap showing scaled expression values of 44 transcription factors differentially expressed between cell types in the P.aa sub-lineage.

Most (56/80) of the postembryonically derived neurons are generated from 13 P cell progenitors (P0 – P12) and the majority of these differentiate as motor neurons. In the newly born L1 larva, P1-P12 appear as left-right pairs of cells on either side of the body; P0 (aka “W”) is positioned in the anteriorly located retrovesicular ganglion. At the mid L1 stage, P1-P12 progenitors migrate toward the ventral cord and initiate a stereotypical pattern of cell division in the late L1 (Figure 3A). The central six P cells (P3-P8) give rise to identical lineages: The anterior daughter, Pn.a, generates five classes of ventral cord motor neurons (VA, VB, VC, VD, AS), while the posterior daughter, Pn.p, gives rise to an epidermal cell (Figure 3B). P cells at the anterior (P0-P2) and posterior (P9-P12) ends of the nerve cord adopt related patterns of division (Supplemental Figure 5B)^4,58^. We used nuclear localized *hlh-3p::tdTomato* to label P cells and progenitors^59,60^ for FACS isolation and scRNA-Seq during three intervals: Before P cell migration to the ventral cord (11–14 hph, “hours post hatch”), during P cell divisions (15.5–18.5hph), and after generation of post-mitotic neurons (21–24hph) (Figure 3C–D).

From these combined data sets, we identified 39,054 cells spanning the P-lineage, including 10 progenitor classes (Pn, Pn.p, Pn.a, Pn.aa, Pn.aaa, P0, P0.a, P0.p, P0.aa/P1.aaa) and 6 post-mitotic neuron classes (VA, VB, VC, VD, AS, AVF) (Figure 3E; Supplemental Figure 5A) (see Methods). P cell-derived progenitors were identified by expression of cell cycle genes (e.g., *cdk-1*)^36,37,61^ (Figure 3G) vs post-mitotic cells that express markers (e.g., *sbt-1*)^62,63^ for mature neurons (Figure 3H). Notably, we observe strong concordance with a set of 49 transcription factors previously assigned to post-embryonic neuronal progenitors on the basis of GFP reporter analysis^64^ (Supplemental Figure 5N). Specific progenitor types (e.g., Pn.aa, Pn.ap) were annotated by expression of known marker genes (Figure 3J–K; Supplemental Table 4). Notably, P-lineage clusters form continuous trajectories in the UMAP embedding with the earliest progenitors at one end of the projection and post-mitotic neurons at the other (Figure 3E–F). The trajectory reflects the pattern of known cell divisions and suggests gradual transcriptional changes as cells divide and mature.

### P0-P1 lineages are transcriptionally distinct from P2-P12 lineages

Interestingly, two distinct groups of cells in the UMAP projection express markers of P-lineage cells. One group includes VA and VB neurons branching from one progenitor cluster (Pn.aaa) and VD and AS neurons branching from a separate group of progenitor cells (Pn.ap) (Figure 3E). These trajectories align with known cell divisions of P2-P12 progenitors (Figure 3B, S5B) (P12.ap gives rise to PDB instead of AS). An additional group of P-lineage cells includes VB and AVF neurons branching from one progenitor cluster and VA and VD neurons branching from another progenitor cluster (Supplemental Figure 5C), trajectories that align with known cell divisions of P0-P1 progenitors (Figure S5B). To confirm the identities of P0-P1 and P2-P12 derived cells, we examined Hox genes, because transcriptionally distinct subclasses of adult motor neuron classes are distinguished by unique combinations of Hox gene expression. Adult motor neurons derived from P0-P1 (e.g., VA1, VD1, VB2, VB1) do not express Hox genes (with the exception of AS1 and VD2, which both express an endogenous *ceh-13* reporter^28^), whereas other P-lineage-derived motor neurons express at least one of the Hox genes *ceh-13, lin-39,* or *mab-5*^28^. On that basis, we confirmed that P0-P1 derived cells in our dataset lack Hox gene expression (Supplemental Figure 5D–F), whereas P2-P12 derived cells show consistent expression of Hox genes (Supplemental Figure 5H–J).

At later developmental stages, each anatomical ventral cord motor neuron class can be further categorized into transcriptionally distinct subclasses that form independent clusters in UMAP space^16,28^. In our profile of L1-stage cells, P1-P12 derived neuron classes did not separate into subclusters, except for VB1 and the VD subclasses VD2, VD12, and VD13 (Figure 3E, Supplemental Figure 5K–L). Other subclusters of VA, VB, VC, and AS motor neurons previously identified in the adult (e.g., VA1, VA2, VA11, VA12) were not observed at the L1 stage (Supplemental Figure 5M), despite capturing an equal or larger sample size for each neuron type and detecting a similar or greater number of genes per cell (Supplemental Table 6). These findings suggest that transcriptional differences among some postembryonic motor neuron subclasses may not arise until later in development.

### Differential expression of transcription factors across the P lineage

To identify candidate regulators of cell fate determination in the P-lineage, we examined differential gene expression across the lineage. We generated a list of differentially expressed genes between every pair of parent-daughter cells and sister cells in the P0 and P1-P12 sub-lineages (Supplemental Table 7). To illustrate our results, we highlight 44 transcription factors that are differentially expressed across the Pn.aa sub-lineage that gives rise to VA, VB and VC motor neurons (Figure 3K). This list should serve as a useful starting point for investigating the gene regulatory mechanisms that drive motor neuron differentiation.

### Defining gene expression across the L1 nervous system

We performed dynamic thresholding^16,28^ of our L1 data (see Methods) to generate aggregated expression tables at four thresholds (available in the L1 CengenApp at cengen.shinyapps.io/L1app). We used threshold 2 for most subsequent analyses. The number of genes detected per neuron class (median = 5478, range = 2266 [M5] to 8438 [VD12]) was positively correlated with the number of cells captured per neuron class (median 357 cells, range = 20 [RMH] to 6216 [AIZ]; Supplemental Figure 6A, Spearman rank correlation = 0.366; p = 2.477 × 10^−5^). We noted 13 neuron classes with the lowest numbers of detected genes and true positive rates compared to the ground truth, labeled in Supplemental Figure 6B. Several of these neuron classes were sparsely sampled (e.g., DVA with 29 cells, ADE with 38 cells) and consequently are likely to include higher numbers of false negatives.

**Figure 4.**
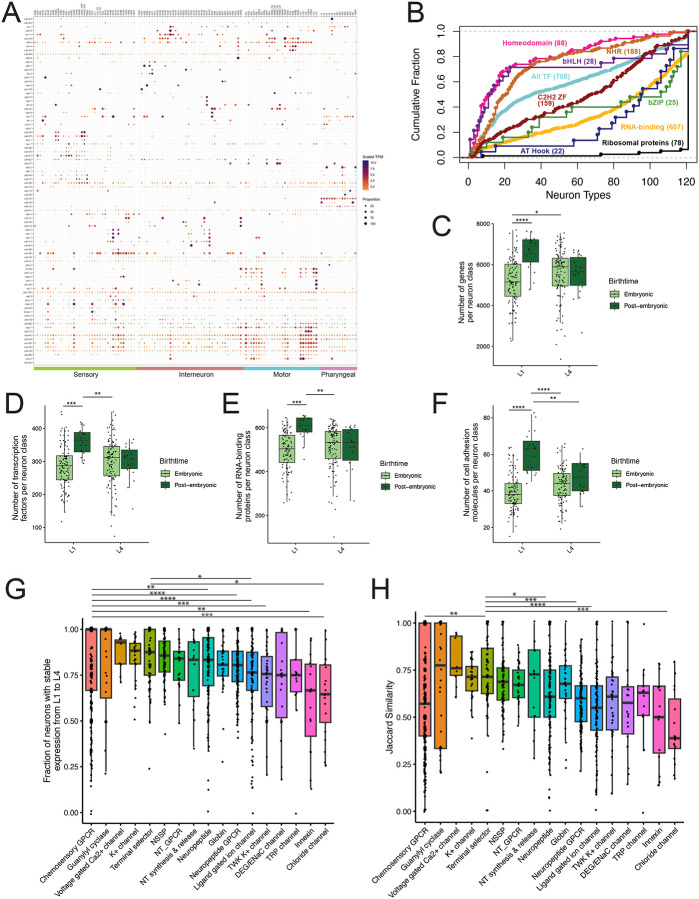
Expression of transcription factors, RNA-binding proteins and cell adhesion molecules in the larval nervous system A) Dot plot showing expression of homeodomain transcription factors across 121 neuron types at the L1 stage. Neuron types are grouped by functional category (bottom). Transcription factors are clustered. Each dot is colored by scaled TPM value. The size of the dot represents the proportion of cells expressing that transcription factor. B) Cumulative distribution of various transcription factor families across 121 neuron types at the L1 stage. The number in parentheses denotes the total number of genes expressed from each category C) Quantification of the total number of genes expressed at threshold 2 in each neuron class at the L1 (left) and L4 (right) stages. Neuron types are divided by birthtime (embryonic vs post-embryonic). D) Quantification of the total number of transcription factors expressed at threshold 2 in each neuron class at the L1 and L4 stages. E) Quantification of the total number of RNA binding proteins expressed at threshold 2 in each neuron class at the L1 and L4 stages. F) Quantification of the total number of cell adhesion molecules expressed at threshold 2 in each neuron class at the L1 and L4 stages. All statistical tests were performed using linear models featuring the birthtime, stage, and number of cells per neuron class as covariates. Between group comparisons were performed on the estimated marginal means using the Tukey p-value adjustment for multiple comparisons. G) Combined box and jitter plots of gene expression stability from L1 to L4 for several gene families with neuronal function. This comparison is restricted to embryonically born neurons. Stability is measured for each gene by the fraction of L1-expressing neurons which still express the gene at L4. H) Combined box and jitterplots of Jaccard similarity between L1 and L4 for neuronal gene families. The Jaccard similarity index accounts for both loss and gain of expression between the L1 and L4 stages. Only a subset of significant comparisons are shown, designated by horizontal lines. For G-H), Kruskal-Wallis tests followed by Dunn’s multiple comparison test. * p < 0.05, ** p < 0.01, *** p < 0.001, **** p < 0.0001.

We catalogued expression of gene families with key roles in the nervous system, e.g., transcription factors, cell adhesion molecules, and neuropeptides and their receptors. To illustrate our findings here, we note that members of the homeodomain family of transcription factors, in most cases, are sparsely expressed across neuron types (Figure 4A–B) in patterns, as noted below, very similar to their expression at the L4 stage. In contrast, other families of transcription factors (e.g. AT-Hook, bZIP) are more broadly detected (Figure 4B). Similar results were obtained at the L4 stage, indicating that these characteristic patterns of expression for transcription factor classes are largely established early in neuronal development.

We detected more genes per neuron class in the L1 in postembryonically derived neurons compared to embryonically born neurons (Figure 4C). We noted a similar pattern for several gene families: In the L1, postembryonically derived neurons expressed more transcription factors, RNA binding proteins and cell adhesion proteins per neuron than embryonically derived neurons. No differences were seen at L4 (Figure 4D–F). The increased number of cell adhesion molecules at early larval stages compared to later larval stages is consistent with a recent report characterizing expression of cadherins across larval development^65^. For two gene families, we observed the opposite effect; postembryonically born neurons expressed fewer GPCR neuropeptide receptors and metabotropic receptors in comparison to embryonic neurons in the L1 and in comparison to both embryonic and postembryonic neurons at the L4 stage (Supplemental Figure 6E–F). No differences were observed between postembryonically derived and embryonically born neurons for the number of ribosomal genes or ligand-gated ion channels expressed per neuron class (Supplemental Figure 6C–D). We suggest that these results could point to gene families (e.g., cell adhesion proteins) that are upregulated in neurons newly born in the L1 stage to promote post-mitotic differentiation versus neurons that are generated earlier in the embryo. Conversely, lower expression of GPCRs and metabotropic receptors in postembryonically derived neurons in the L1 could indicate that the signaling function of these neurons is immature at this stage.

### Stability of gene expression between the L1 and L4 nervous systems

The anatomy and function of the larval nervous system are extensively modified during development. Postembryonic neurons are generated after hatching and neurons throughout the nervous system expand in size and add new synapses during larval development^4,6^. Some embryonic neurons alter their anatomy, by either elaborating or pruning neurites^7,8,66,67^, whereas others remodel the locations of synaptic inputs and outputs^9–11,68^. L1 larvae show distinct patterns of locomotory behavior^12,13^ and odor preferences compared to L4 larvae^14,15^. These differences likely arise from changes in gene expression in the nervous system during larval development.

For embryonically born neurons, we investigated the stability of expression from L1 to L4 of gene families with neuronal functions. Using threshold 2, we asked whether each gene is expressed in a given neuron only at L1, at both L1 and L4, or only at L4. We calculated, for each gene, the fraction of neurons with expression at L1 that was maintained at L4. Most neuronal gene families had high levels of stability (Figure 4G), with medians of 80% of neurons having stable expression. Chemosensory GPCRs, guanylyl cyclases, voltage-gated Ca2+ channels, K+ channels and terminal selector transcription factors all had highly stable expression. Chloride channels and innexins had the least stable expression (Figure 4G).

To include a measure that accounts for expression at L4 only, we calculated a Jaccard similarity index (see Methods), as the number of neurons with expression at either stage divided by the number of neurons with expression at both stages (Figure 4H). By this metric, expression of terminal selector transcription factors was significantly more similar across development than several other neuronal gene families, including chemosensory GPCRs, neuropeptides, neuropeptide receptors, ligand-gated ion channels, and chloride channels (Figure 4H). Guanylyl cyclases, voltage-gated Ca2+ channels, K+ channels, and neurotransmitter synthesis and release genes also showed high levels of similarity. Overall, these data demonstrate the stability of several families of neuronal genes, including classical neurotransmitter systems, in the embryonically born nervous system across larval development. Stability in gene expression likely contributes to stable core functionality across the nervous system which is refined throughout larval development.

### Differential gene expression analysis detects widespread changes in gene expression across specific neuron types during development.

**Figure 5.**
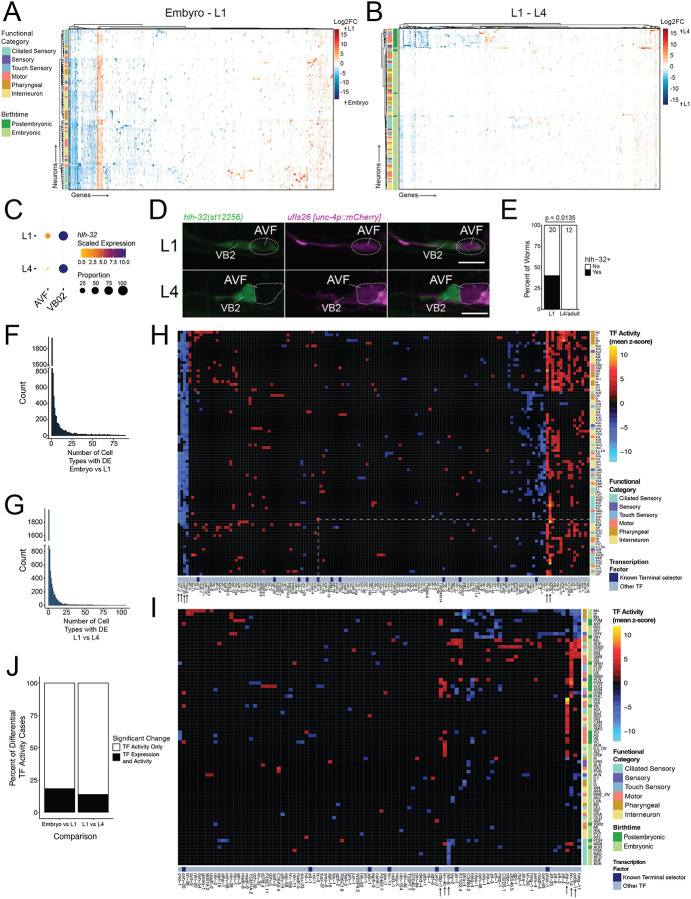
Thousands of genes are differentially expressed across larval development A) Heatmap showing the average log fold change for genes with significant differential expression between embryonic and L1 stages. Positive log fold change values (orange, red) reflect higher gene expression in the L1 whereas negative log fold change values (blue) reflect higher expression in the embryo. Both genes (columns) and neurons (rows) are clustered. Neuronal functional categories are depicted on the left. B) Heatmap as in A, but for differential expression between the L1 and L4. Positive fold change values (red, orange) reflect higher expression in L1, negative values reflect higher expression in L4 (blue). Y-axis labels include functional categories and developmental birthtime (embryonic vs postembryonic) for neurons. C) Dotplot of scRNA-seq data showing expression of *hlh-32* in AVF and VB2 in L1 and L4. D) Confocal micrographs showing expression of endogenously GFP tagged *hlh-32* in AVF in L1, but not L4. Note stable expression in the AVF sister cell VB2. Scale bar = 5 μm. E) Quantification of *hlh-32* expression in AVF in L1 and L4/adult worms, Fisher’s Exact Test. F) Histogram showing the distribution of neuron classes in which a gene exhibited significant differential expression for the embryo vs L1 comparison. G) Histogram as in F, but for the L1 vs L4 comparison. H) Heatmap showing transcription factors (TFs) with significant TF activity (columns) across neurons (rows) between the embryonic and L1 stages. Warmer colors (red-yellow; positive numbers) denote increased expression of a TF regulon in L1. Cooler colors (blue; negative numbers) denote decreased expression of a TF regulon in L1. Neuronal functional categories are color coded, and TFs with known roles as terminal selectors (in any neuron) are denoted in dark blue. Dotted lines denote the increased activity of *hlh-4* in ADL. TFs with significant changes across many neurons are indicated by black arrows. I) Heatmap as in H, showing transcription factors (TFs) with significant TF activity (columns) across neurons (rows) between the L1 and L4 stages. Neurons are color-coded by both functional categories and birthtime, and TFs with known roles as terminal selectors (in any neuron) are denoted in dark blue. TFs with significant changes across many neurons are indicated by arrows. J) Bar graph showing the fraction of instances of differential TF activity which also showed differential TF expression (black) or which lacked significant differential expression (white). Most cases of differential activity of a TF did not show differential expression of the TF at the transcript level.

Prior work has shown that relative levels of transcription factor expression can differentially influence cell fate^69^. We therefore performed differential gene expression analysis on the unthresholded single-cell data between the embryo and the L1 for each of 89 transcriptionally distinct neuron classes detected in both embryonic^24^ and L1 datasets and between the L1 and L4 stages for each of 121 transcriptionally distinct neuron classes detected in both the L1 and L4^16^ datasets. In the embryo to L1 comparison, we detected 54,757 instances of differential expression (i.e., a gene showing differential expression between ages in a given neuron) featuring 5941 differentially expressed genes (DEGs) among the 89 neuron classes (Figure 5A, Supplemental Table 10). In the L1 to L4 comparison, we detected 36,350 instances of differential expression featuring 5810 DEGs among the 121 neuron classes (Figure 5B, Supplemental Table 11).

Our list of DEGs overlapped significantly with bulk RNA sequencing of pooled neuronal nuclei from L1 and L4 larvae and from images of fluorescent reporter strains for 18 genes^12^. We found concurrence with cases in which a gene was reported higher in the L4 than L1 and with instances with stable expression (Supplemental Figure 7A). We observed little overlap, however, with cases where imaging results reporting higher expression in L1 than L4. Some of these discrepancies could be due to disparate timing for L1 imaging and scRNA-seq experiments or to differences in the perdurance of mRNA vs GFP-tagged protein reporters^70–72^.

We used endogenously tagged reporter strains to validate additional instances of differential expression between the L1 and L4 stages (Figure 5C–E, Supplemental Figure 7B–F). We confirmed decreased expression of the basic helix-loop-helix transcription factor *hlh-32* in AVF in the L4 vs L1 (Figure 5C–E). We validated two instances of differential expression in CAN, with both the insulin-like neuropeptide *ins-30* and the noncoding RNA *lep-5* showing no expression at L1 and clear expression in L4 animals (Supplemental Figure 7B–D). We validated differential expression for the neuropeptides *srlf-1* in HSN and *flp-27* in ALM and PLM, both of which decreased from L1 to L4 (Supplemental Figure 7B, E, F). Imaging of endogenous reporters for *nlp-64*, *nlp-73, ins-5, flp-32* and *flp-5* also largely confirmed the scRNA seq DE results (Supplemental Figure 8A–E).

We performed gene set enrichment analysis of the differentially expressed genes using Wormcat^73,74^. The embryo vs L1 DEGs showed enrichment of neuropeptides, globins, ribosomal proteins and genes related to neuronal function (Supplemental Figure 9A, B). The L1 vs L4 DEGs were enriched for neuropeptides, globins, mitochondrial complex 1, ribosomal proteins and heteromeric G protein receptors (Supplemental Figure 10A, B). To identify patterns across the nervous system and the genome for each age comparison, we clustered both neurons and genes based on differential expression profiles (Figure 5A, B). Across both comparisons, most genes showed significant differential expression in a small number of neuron classes (Figure 5F–G, median number of neurons with DE per gene = 3 in both comparisons). Some genes showed differential expression across a broader array of neurons especially at younger vs older developmental stages in each comparison. These broadly differentially expressed gene clusters were enriched for genes involved in basic cellular functions, including ribosomal, mitochondrial and ER-resident proteins (Supplemental Figure 9B, Supplemental Figure 10B).

Transcripts encoding most ribosomal proteins, including *rps-4*, showed broad transient upregulation in neurons from embryo to L1 (Supplemental Figure 9A, B), with significant downregulation by L4 (Supplemental Figure 10A, B). Four genes (*rpl-25.1, rpl-27, rpl-7A,* and *rps-16*) showed a distinct pattern, with little change from embryo to L1, and widespread higher expression in L4 neurons (Supplemental Figure 11A). We imaged endogenously tagged versions of three ribosomal proteins (RPL-25.1, RPL-7A, RPS-4) to validate these findings (Supplemental Figure 11B–D). RPL-7A protein levels moderately decreased from L1 to L4, which differs from the pattern of *rpl-7A* transcripts (Supplemental Figure 11B). However, changes in RPS-4 and RPL-25.1 protein levels were consistent with transcript changes in the scRNA-seq data. We found that RPL-25.1 was expressed at low levels in neurons at L1 but at significantly higher levels in neurons at the L4 stage and RPS-4 protein levels showed a moderate decrease in expression from L1 to L4 (Supplemental Figure 11C–D). These data suggest highly dynamic regulation of protein translation during *C. elegans* nervous system development, consistent with reports from other organisms^75–77^.

Only five genes, *lep-5, pgal-1, ctc-3, hsp-70, pab-1*, showed broadly higher expression in the L4 compared to L1 (up in L4 in >50% of neuron classes). The gene with broadest upregulation from L1 to L4 was the non-coding RNA *lep-5* (higher in L4 in 101 neuron classes), which has been shown to promote the larval to adult transition and sexual maturation^78,79^. Additionally, we noted broad differential expression of several heat-stock related proteins (*hsp-70, hsp-16.48, hsp-16.41, F44E5.5, F44E5.4*). In most cases these genes showed higher expression in L4, but some genes, such as *hsp-16.48,* were higher in L1 neurons, indicating possible differences in stress responses between L1 and L4.

We also noted patterns of genes that featured shared differential expression among subsets of neurons. In both age comparisons, ciliated sensory neurons largely clustered together. In the embryo vs L1, two clusters of genes showed shared patterns of higher expression in the embryo across many ciliated sensory neurons (Supplemental Figure 9A, dark blue). These two clusters were strongly enriched for genes involved in non-motile cilia formation and function (Supplemental Figure 9B), indicating downregulation of their expression in L1 compared to the embryo. An additional cluster of genes (Supplemental Figure 9A, denoted in red-orange) contained several genes that show higher expression in L1 in restricted subsets of ciliated sensory neurons. This cluster showed significant enrichment of insulin-related genes, *irld-* family genes (denoted as “receptor L domain”) and several families of seven transmembrane domain G-protein coupled receptors (Supplemental Figure 9B). We observed additional gene sets enriched for G-alpha subunits, nuclear hormone receptors and GPCRs among the DEGs in ciliated neurons between L1 and L4 (Supplemental Figure 10A, B). These results highlight a pattern of shared downregulation of genes involved in initial cilia development followed by neuron-specific upregulation of genes that contribute to individualized functional states.

In the L1-L4 comparison, most postembryonically derived neurons clustered together. These neurons featured shared differential expression of a subset of genes with higher expression in the L1 than L4 (Figure 5B, dashed box, Supplemental Figure 10A, B). Many of these same DEGs were higher in the embryo compared to the L1 stage across much of the embryonically derived nervous system (Supplemental Figure 9A). We identified a set of 41 genes that were broadly higher in the embryo compared to L1 among embryonic neurons and broadly higher in L1 than L4 in postembryonically derived neurons (see Methods). These genes included cell adhesion molecules (*fmi-1, eva-1, sdn-1*), genes involving in protein folding and processing (*ric-3, cnx-1, ugt-50, cpi-2, crt-1, ZK792.7)*, transcription factors or chromatin modifiers (*hbl-1, syd-9, hmg-1.1, ldb-1, F54C8.4*), histones (*his-24, hil-7, htz-1, hil-2*) and several genes with various or uncharacterized functions (*C23F12.4*, *ZK287.1, hrp-2, cav-1, aqp-2, asp-4, cki-1, W04A8.4, far-1, F26F12.3, tts-2, F59B2.8, T05E11.9, Y7AD9.1, F15G9.1, C06G3.6, lsm-5, prcc-1, ret-1, scpl-3, T10B5.4, ubq-1, Y54E10BL.3*).

These findings suggest that newly born neurons of all functional categories downregulate similar genes in their early post-mitotic state. Consistent with this idea, the earliest generated postembryonic neurons AQR and PQR do not show higher levels of this cohort of developmentally down-regulated genes in L1 compared to L4, suggesting that AQR and PQR have downregulated early genes by the late L1 period. We also identified distinct subsets of genes that showed a more restricted pattern of shared differential expression only among postembryonic ventral cord motor neurons. Genes involved in mRNA splicing, the proteasome and chromatin modification were significantly enriched in this group (Supplemental Figure 10A, B, magenta). Together with the patterns described above for ciliated neurons, these data indicate that: 1) all neurons downregulate a shared set of genes in their early post-mitotic state, 2) subsets of functionally related neurons (e.g., ciliated neurons, VNC motor neurons) downregulate distinct sets of genes during early maturation, and 3) subsequent maturation across the nervous system largely entails cell-type specific differential expression rather than a pan-neural genetic maturation program.

### Heterogeneity in gene-level differential expression

A substantial number of genes showed differential expression in more than one neuron class. Such genes could exhibit three possible patterns: 1) higher expression in the younger stage in all neuron classes in which they are differentially expressed, 2) higher expression in the older stage in all neuron classes in which they are differentially expressed, or 3) mixed changes, with higher expression in the younger stage for some neuron classes but higher expression in the older stage in other neuron classes. Between the embryo and L1, ~80% of genes with DE in > 1 class (3192/4011) consistently changed expression in the same direction across neurons (i.e., either higher in the embryo or higher in L1). For example, the H1 histone gene *hil-7* is differentially expressed in all 89 neuron classes between embryonic and L1 stages and is more highly expressed in the embryo in all cases. Between L1 and L4, 53% (2014/3823) of genes consistently changed expression in the same direction. For example, the neuropeptide processing gene *pgal-1* is higher in the L4 stage in all 92 neuron classes in which it was differentially expressed. A substantial number of genes, however, (819/4011, 20%, in embryo–L1 and 1809/3823, 47%, in L1-L4) exhibited changes in opposite directions depending on the neuron class. For example, the cysteine synthase enzyme *cysl-1* was differentially regulated in 34 neuron classes between embryonic and the L1 stages. *cysl-1* was higher in the embryo in 17 neuron classes, but higher in the L1 stage in 17 other neuron classes. Between L1 and L4, the neuropeptide *flp-16* was differentially expressed in 16 neuron classes, with higher expression in the L1 stage in eight neuron classes and higher expression in the L4 stage in eight neurons. These results further highlight the cell-type specificity of neuronal maturation.

At the neuron level, 571 genes (median) were differentially expressed per neuron class for the embryo vs L1 comparison (Supplemental Figure 9C–D) and 227 genes for the L1 vs L4 comparison (Supplemental Figure 10C–D). No significant differences were seen in the number of DEGs between functional categories in the embryo vs L1 comparison (Supplemental Figure 9D). In the L1 vs L4 comparison, no significant differences were present in the number of DEGs between functional categories (Supplemental Figure 10C–D), but postembryonic neurons exhibited significantly more DEGs than embryonic neurons (Supplemental Figure 10E–F). HSN was a notable exception, with the third highest number of DEGs after the larval VC and VD neurons. The HSN neuron pair exhibits delayed maturation compared to other embryonic neurons^32,80^. HSN neurons are generated and migrate to the midbody region in the embryo, but do not acquire neuronal features until the fourth larval stage^32,80,81^. Among the 193 genes expressed at higher levels in HSN in the L1, we identified several genes that indicate possible roles of HSN in the early larval nervous system. At L1, but not L4, HSN expressed transcripts of the polycistronic innexin genes *inx-12* and *inx-13* (Supplemental Table 11). These innexins are primarily expressed in non-neuronal tissues, including the epidermis, and their expression in HSN could facilitate communication between HSN and these tissues in early larval development. We also detected higher transcript levels in HSN at the L1 stage for genes involved in neuronal signaling, including the neuropeptide *srlf-1* (Supplemental Figure 7B, E) and the putative acetylcholine gated chloride channel *lgc-47* (Supplemental Table 11). Several transcription factors were expressed in HSN in L1 but not L4, including *ham-2, bed-3* and the SoxC homolog *sem-2*. Many of these same genes are expressed at higher levels in L1 in the VC neurons, which also have delayed maturation. Notably, in the postembryonic M lineage, *sem-2* antagonizes terminal differentiation of muscle cells^82^ which suggests that *sem-2* may be acting to pause full neuronal maturation in HSN and VC.

### Gene regulatory network analysis identifies putative drivers of developmental changes in gene expression

To identify possible transcriptional regulators driving differential gene expression across developmental stages, we used the recently described *C. elegans* Estimation of Transcription Factor Activity (*Cel*EsT) gene regulatory networks (GRNs)^83^. This resource contains TF-target data for 487 TFs, determined from a combination of DNA-binding motif analysis, ChIP-seq and enhanced yeast one-hybrid (eY1H) screens, with an average of 829 targets per TF. We reasoned that it should be possible to infer the activity of a given TF from expression of its targets^84^. We used the decoupleR package^85^ to estimate transcription factor activity based on the log fold changes of all genes for each neuron. We performed this analysis separately for the embryo to L1 and the L1 to L4 comparisons. We filtered the results to retain only TFs with significant activity (Benjamini-Hochberg adjusted p-values < 0.05) and in which the TF was detected in the queried neuron in at least one of the ages being compared. From the embryo versus L1 differential expression data, we identified 966 cases of significant TF activity for 151 distinct TFs in the DEGs of 89 neuron classes (Figure 5H, Supplemental Table 12). For the L1 versus L4 comparison, we detected 355 instances of significant activity for 102 distinct TFs among DEGs across 78 neurons (Figure 5I, Supplemental Table 13).

Several identified instances of TF activity are consistent with known roles for these TFs in specific neurons. For example, in the ADL neuron, the basic helix-loop-helix TF HLH-4 showed the highest level of activity in the L1 versus the embryo comparison (Figure 5H), a finding consistent with HLH-4 serving as terminal selector of ADL fate^86^. The *fkh-8* forkhead TF, a direct regulator of sensory cilia gene expression^87^, showed significant activity between the embryo and L1 primarily in ciliated sensory neurons. We detected significantly decreased activity of the proneuronal factor NeuroD homolog *cnd-1* from the embryo to the L1 stage in 50 neuron classes, a finding in line with the broad role of NeuroD in promoting neuronal differentiation^88–90^. Several additional transcription factors exhibited significantly decreased activity across a broad set of neurons from the embryo to L1 stage, including *ceh-39* (75 neuron classes), *hmg-4* (63 classes), and *nhr-2* (40 classes). These data are consistent with the known roles of *nhr-2* in embryonic development and subsequent downregulation^91^ and indicate that *ceh-39* and *hmg-4* may also be involved in the early stages of nervous system patterning.

We noted broad changes in activity for the *lin-14* heterochronic TF. *lin-14* is expressed at the highest levels in the embryo and early L1 stages and is downregulated by *lin-4* miRNA with entry into the L2 stage^92–94^. Multiple studies have identified roles for *lin-14* in age-related changes in the nervous system^12,79,95–99^. Our data showed significant activity of *lin-14* (i.e., increased *lin-14* target expression) from embryo to L1 in 55 neuron classes and from L1 to L4 in 17 neuron classes. The observed elevation of in *lin-14* targets with age is correlated with the known age-related decrease in *lin-14* expression and suggests that LIN-14 functions primarily as a transcriptional repressor in these neurons. Our data support a model wherein *lin-14* exhibits a broad role in driving the postembryonic maturation of the nervous system, consistent with a recent finding from bulk RNAseq data^12^.

From the L1 to L4 comparison (Figure 5I), we identified additional TFs with activity patterns suggesting broad regulation (across several neuron classes) of temporal gene expression including the bZIP proteins, *zip-2* (24 neuron classes) and *cebp-1* (21 neuron classes), the GATA factor *lin-40* (10 neuron classes), and the C-terminal binding protein-1 *ctbp-1* (8 classes). These TF genes are known to function in immune responses (*zip-2*)^100,101^, aging (*zip-2*^100^ and *lin-40*^102^), axon regeneration (*cebp-1*^103^), stress resistance (*lin-40*^102^) and maintenance of neuronal identity (*ctbp-1*^104^). Our data suggest that these TFs may also fulfill previously undescribed roles in neuronal maturation during larval development. The neurons with elevated *zip-2* activity were almost completely non-overlapping with the set of neurons showing increased *lin-14* target expression (Figure 5I), indicating that ZIP-2 and LIN-14 may regulate postmitotic development of distinct subsets of neurons.

In contrast to the broadly acting TFs described above, most TFs show more restricted patterns of significant activity (median of significant activity in 2 neurons for the embryo to L1, and 1.5 neurons in the L1 to L4 comparison). This finding is consistent with our differential gene expression results showing largely neuron class-specific changes in gene expression from L1 to L4. Although nearly all known terminal selector TFs are included in the *Cel*EsT GRNs, these TFs comprise only a small fraction of the TFs with significant activity among DE genes, indicating that terminal fate regulator activity is stable in post-mitotic neurons. Strikingly, most TFs with differential activity (as measured by changes in regulon expression) in a given neuron did not exhibit differential expression (as measured by changes in TF transcript) (Figure 5J). This finding points to the possibility of widespread regulation of TF activity at the post-transcriptional level during nervous system development and highlights the value of gene regulatory network analysis.

Overall, our analysis identified an extensive list of thousands of differentially expressed genes across the nervous system at single neuron resolution between the embryo and the L1 stage and between the L1 and L4 stages. We additionally uncovered potential roles for dozens of TFs in driving differential expression in the developing nervous system. These data provide a comprehensive resource for investigating the maturation of post-mitotic neurons and for associating alterations in gene expression with behavioral changes during larval development.

### Neuropeptidergic networks in the L1 larval stage

In addition to classical small molecule neurotransmitters, the *C. elegans* nervous system also features “wireless” networks of neuropeptides and neuropeptide receptors^16,105–107^. Neuropeptide-encoding genes were significantly enriched in the differentially expressed genes throughout larval development (Supplemental Figure 9B, Supplemental Figure 10B) and exhibited some of the highest log fold changes (Supplemental Tables 10, 11).

**Figure 6.**
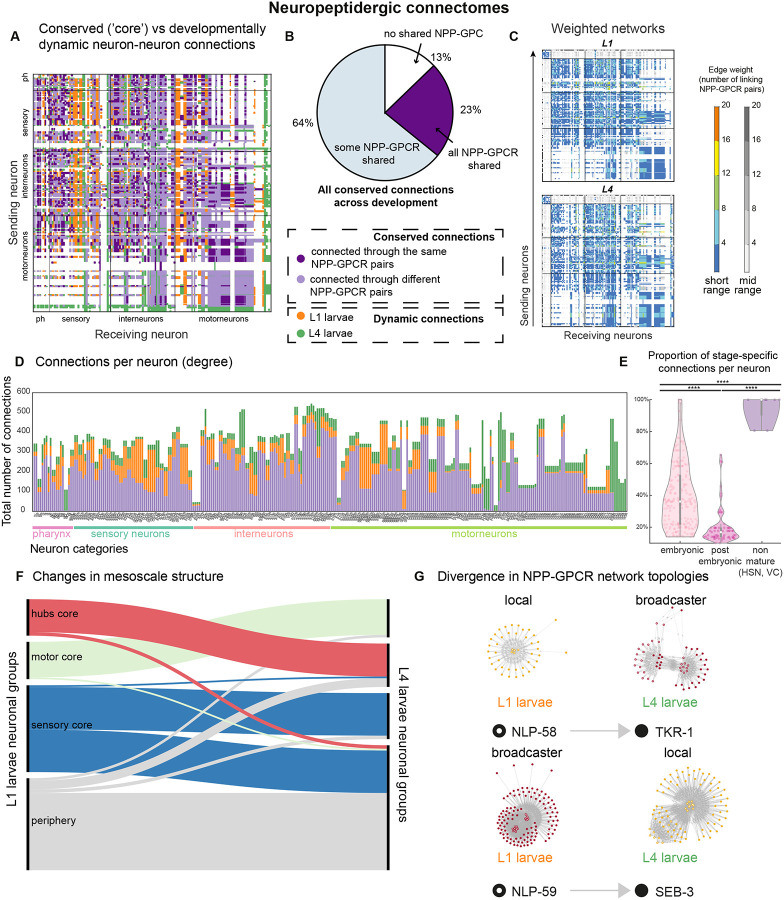
Developmental alterations in neuropeptide networks A) Thresholded neuropeptidergic connectome (mid-range) showing the developmental pattern of connections between sending neurons (y-axis) and receiving neurons (x-axis). Core (conserved) connections and developmentally dynamic connections are color-coded. Only neuropeptide-receptor couples with functionally validated threshold of EC_50_ < 500 nM binding *in vitro*^105,106^ were included. Neurons are grouped by functional category (Ph = Pharyngeal). B) Percentage distribution of the conserved connections between NPPs (Neuropeptide Precursor genes) and GPCRs (G-Protein Coupled Receptors) forming the core connectome. 13% of those connections do not share a common NPP-GPCR pair across development. The other 87% connections share at least one NPP-GPCR pair (64%) or all NPP-GPCR pairs (23%). C) Weighted neuropeptidergic connectomes, indicating for every cell-cell connection how many pairs of neuropeptide-receptors mediate the connections. D) Total number of degrees (y-axis, mid-range networks) in homologous neurons across development (x-axis) classified by functional categories. Bars are color-filled according to the subsets of core connections and developmentally dynamic neuropeptides (See key in B) of each neuron across development. E) Proportion of developmentally dynamic neuropeptide connections (y-axis) per neuron classified by embryonic development (colors and x-axis; “non-mature” refers to the HSN and VC neurons). Kruskal-Wallis test with Dunn’s correction, ****P < 0.0001, ***P < 0.001, *P < 0.025. F) Variation in mesoscale grouping across development. The same 4 groups (hubs core, sensory core, motor core, periphery) are conserved but some individual neurons change between groups through development. G) Network topologies of the indicated NPP/GPCR pairs. Empty circles represent neuropeptide expression, filled circles represent receptor expression. Local networks display restricted NPP and GPCR expression (≤50 neurons), pervasive networks display broad NPP and GPCR expression (>50 neurons), broadcaster networks display restricted NPP but broad GPCR expression, integrative networks display broad NPP but restricted GPCR expression. NLP-58 and NLP-49 = NPPs; TKR-1 and SEB-3 = GPCRs

By combining data on biochemical neuropeptide-receptor interactions^105^, nervous system-wide expression profiles^16^ and anatomical positioning of neurons^6,26,108^, we recently described neuropeptidergic networks in the L4 stage^106^. Following the same procedure, we analyzed the L1 neuropeptide network (Figure 6A–C) and compared it to the L4. In these networks, a connection or edge between two neurons is defined when one neuron expresses a specific neuropeptide ligand, another neuron expresses the biochemically validated cognate receptor, and both neurons are within a diffusion range determined by spatial constraints on signaling. The L4 larval neuropeptide network was built using the most stringent threshold for expression (threshold 4), and neuropeptide-receptor pairs that show high-potency interactions (EC_50_ ≤ 500 nM), including 49 neuropeptide precursor (NPP) genes and 51 GPCRs^16,105,106^. The diffusion range was established by defining a matrix of neuronal proximity, in which we used electron microscopy (EM) data of the L4 nervous system to assign neurons to specific process bundles^26^.

For the L1, we focused on 45 neuropeptide precursor genes (NPPs) and 47 GPCRs that are expressed in both the L1 and L4 stages, forming 88 validated ligand-receptor pairs with high-potency interactions (EC_50_ ≤ 500 nM). Transcripts for the neuropeptides *flp-23*, *nlp-22*, *nlp-27*, *nlp-25* and the receptors *npr-41, dmsr-16, gnrr-3,* and *dmsr-11* were detected in the nervous system at the L4 but not at the L1 stage. We built a matrix of neuronal proximity using EM data of the L1 nervous system 16 hours after hatching to define diffusion range^6,9^ (Supplemental Figure 12A–B). For the L1 network we focused on short-range diffusion, which allows neuropeptide diffusion between neurons that belong to the same process bundle (~10nm), and mid-range diffusion, which allows neuropeptide diffusion between neurons that are in the same anatomical area (head, midbody, tail) (<50nm).

To compare *C. elegans* neuropeptide networks at the L1 and L4 stages, we limited our analysis to the 291 neurons present at both the L1 and L4 stages. Across the 88 NPP-GPCR pairs, 39% (35 pairs) displayed expression changes that led to alterations in network topology (Supplemental Figure 13), but the global architecture of the neuropeptide network is largely similar in the L1 and L4 (Fig 6A–C). Between 77% and 79% of neuron-to-neuron connections (depending on the developmental stage and the network range) are conserved across the two stages. Among these, ~23% (5310/23207 in short-range and 7390/29801 in mid-range) of conserved connections use identical NPP-GPCR pairs, ~64% share at least 1 common NPP-GPCR pair, and ~13% are topologically conserved but involve different molecular pairs (Figure 6B). L1 networks exhibit a modest 2–3% (depending on the network range) increase in overall connectivity compared to L4 (Figure 6C).

Using the number of connections each neuron receives and sends (degree), we defined a group of highly connected neuropeptidergic neurons at the L4 stage that seem to be morphologically specialized for neuropeptide connectivity^106^. Most of these neurons are embryonically derived and exhibit high degree at the L1 stage as well, with a connectivity correlation >0.8 across stages (Supplemental Figure 14A–B). These data support the notion that these neuropeptide hubs likely form the scaffold of the neuropeptide connectome, with their organizational role established from birth.

Outside of this conserved core, stage-specific connections emerge, particularly in neurons undergoing significant developmental changes. Postembryonically born neurons like HSN, SDQ, VC, PVM, and AVM show >80% stage-specific neuropeptide connectivity (Figure 6D–E, Supplemental Figure 14C). Neuropeptide connectivity in these cases is restricted by shorter processes in undeveloped neurons like HSN and large variations in gene expression between L4 and L1 stages (Supplemental Figure 12B). Among embryonically developed neurons, connectivity divergence is more prevalent in sensory and head motor neurons, such as OLQ, RMD, IL1 and CEP (Figure 6D; Supplemental Figure 14C). These neurons are critical for mechanosensation, a modality known to undergo functional refinement during development^109–111^.

These changes are mirrored in the mesoscale structure of the neuropeptide connectome. The network segregates into a sparsely connected periphery and three densely connected modules composed of motor neurons, highly connected hubs, and sensory neurons^106^. Neurons such as HSN and AVM, initially peripheral at L1, migrate into the hub module by L4, reflecting maturation. Conversely, neurons like OLQ shift from the sensory module to the periphery, suggesting age-related reduction in functional connectivity (Figure 6F; Supplemental Table 16).

Most of the cases in which NPP-GPCR pairs changed topology (22/35) reflected an increase in network breadth as the organism developed, shifting from “local” or “broadcaster” networks in L1 to “broadcaster” or “pervasive” networks in L4. For instance, the *nlp-58/tkr-1* tachykinin-like homologue shows a more specific network in L1, displaying a “local” topology, whereas it has a “broadcaster” topology in L4 (Figure 6G). Some NPP-GPCR pairs, however, showed instances in which the network breadth decreased during development. For example, the *nlp-49/seb-3* network displays a change from a “broadcaster” connectivity at L1, where the receptor is expressed very broadly and the neuropeptide is not, to a “local” network in which both neuropeptide and receptor are expressed sparsely (Figure 6G).

### Identifying molecular determinants of neuronal membrane contact

We next sought to use the L1 transcriptomic data to identify possible molecular determinants of nerve ring structure and connectivity. We restricted our analysis to cell surface proteins and secreted peptide ligands, since these molecules have been shown to regulate axon guidance, fasciculation and synaptogenesis^112–117^. Importantly, we also incorporated data from the literature and recent work which have directly measured protein-protein interactions between the extracellular domains of many of these proteins^118^. We compiled a list of 443 transmembrane, GPI-anchored or secreted proteins (collectively referred to as cell adhesion molecules, CAMs) that could potentially provide contacts or instructive communication between adjacent cells^118–122^. Of these, we detected expression of 298 (69%) genes in the L1 nervous system. Neurites in the *C. elegans* nerve ring have been grouped into five bundles or strata^123,124^ based on shared contacts. We detected enriched expression of two genes in the neurons of a specific stratum (as defined^123^) compared to all others: higher expression of *pan-1* in stratum 1 and higher expression of the Ephrin *vab-2* in stratum 4.

**Figure 7.**
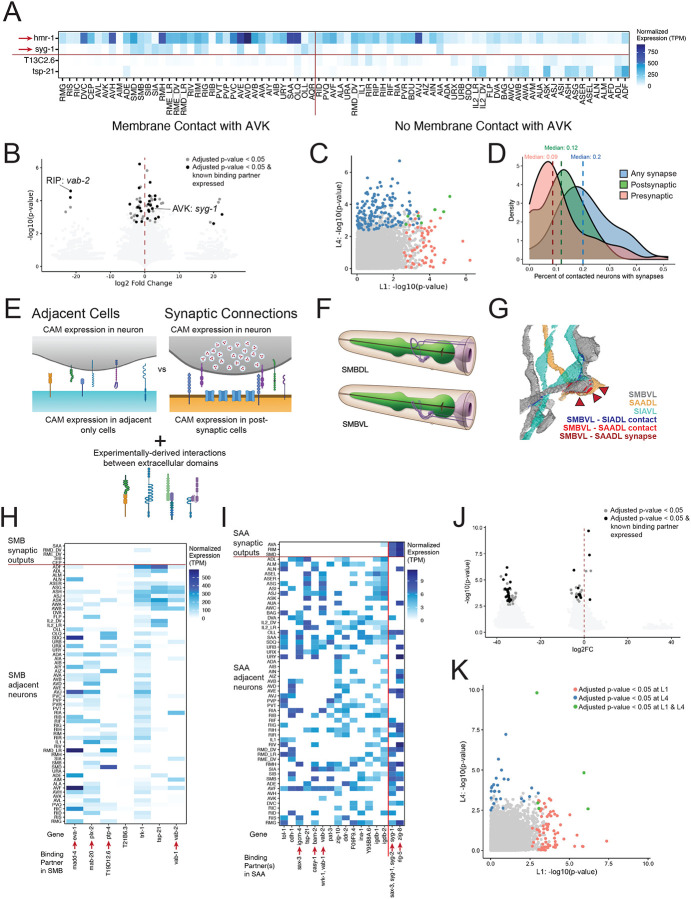
Differential expression of secreted and cell surface molecules based on patterns of membrane contact and synaptic connectivity A) Heatmap showing enrichment of *hmr-1* and *syg-1* in neurons contacting AVK (left of vertical red line) compared to neurons not contacting AVK (right of vertical red line). T13C2.6 and *tsp-21* (below horizontal red line) are enriched in cells not contacting AVK. Red arrows indicate genes with known binding partners expressed in AVK. B) Volcano plot of secreted and cell surface enrichment in neurons based on membrane contact. Each dot represents a gene tested for a given neuron. Positive log2 Fold Changes indicate enrichment in cells with membrane contact. Light gray dots represent instances with Benjamini-Hochberg adjusted p-values > 0.05. Dark grey dots represent instances with Benjamin-Hochberg adjusted p-values < 0.05. Black dots represent significant instances in which at least one known binding partner is expressed in the tested neuron (i.e., *syg-1* is enriched in neurons that contact AVK, and the *syg-1* binding partner *sax-3*^172^ is detected in AVK). C) Scatterplot showing Benjamini-Hochberg corrected p-values for each gene/neuron combination testing expression enrichment in membrane contacted vs non-contacted cells in L1 (x-axis) and in L4 (y-axis). Genes only detected at one age are not shown. Pink: significant enrichment only in the L1, blue: significant enrichment only in the L4, green: significant enrichment in both stages. D) Smoothed density plot of the fraction of membrane-contacted neurons with synaptic connections in the L1 nerve ring. The blue curve shows the fraction of membrane-contacted neurons with any synaptic connections (median of 0.2). The green curve represents the fraction of neurons that receive postsynaptic output (median: 0.12), and the pink curve represents the fraction of membrane-contacted neurons that provide presynaptic input (median: 0.09). E) Top: Graphical representation of cell adhesion molecule (CAM) expression in cells with membrane contact only (left) and cells with synaptic connections (right). Bottom: CAM binding partners confirmed by biochemical assays. F) Cartoon representations of the SMB neurons that innervate the nerve ring, from WormAtlas. G) 3D reconstruction from NeuroScan of late L1 nerve ring featuring three neurons with membrane contact sites and synapses. SAADL (orange) has both membrane contact (red patches) and synaptic contacts (red triangles) with SMBVL (gray), whereas SIAVL (light blue) only has membrane contact (dark blue) with SMBVL. H) Heatmap showing normalized expression (TPM) of 7 CAMs enriched in SMB-non-synaptic adjacent cells (below horizontal line) compared to in SMB postsynaptic outputs (above horizontal red line). Red arrows denote genes encoding proteins with known binding partners expressed in SMB. I) Heatmap showing normalized expression of genes enriched in SAA-non-synaptic, adjacent neurons (14 genes, to the left of vertical red line) and genes enriched in SAA-synaptic partners (2 genes, to the right of the vertical red line). Red arrows denote genes encoding proteins with known binding partners expressed in SAA. J) Volcano plot showing enrichment of CAMs based on synaptic connectivity. Each dot represents one gene/neuron comparison. Negative log fold change values represent enrichment in non-synaptic adjacent cells, whereas positive log fold change denote enrichment in synaptically-connected cells. Light gray dots correspond to Benjamini-Hochberg adjusted p-values > 0.05. Dark grey dots represent significant enrichment (Benjamini-Hochberg adjusted p-value < 0.05), and black dots denote significant enrichment with known binding partners expressed in the tested neuron. K) Scatterplot showing -log10 transformed p-values for each gene-neuron combination in L1 (x-axis) and in L4 (y-axis). Genes detected at only one age are not shown. Significant cases (Benjamini-Hochberg adjusted p-value <0.05) of enrichment only in L1 (pink), significantly enriched only in L4 (blue), and cases enriched at both L1 and L4 (green). The p-value adjustments were made for multiple testing within each neuron.

To gain additional insight into the molecular regulation of membrane contact, we tested the enrichment of genes in contacted vs non-contacted cells for each of the 80 neurons in the L1 nerve ring. Membrane contact is a prerequisite for synapses, and the extent of membrane contact can be predictive of synaptic connectivity^26,108,125^. We detected 62 instances of gene enrichment in either contacted or non-contacted cells (Supplemental Table 17). For example, the cadherin *hmr-1* and the immunoglobulin superfamily adhesion molecule *syg-1* were significantly enriched in cells that have membrane contact with AVK vs cells with no AVK contact, whereas the transmembrane low density lipoprotein receptor *T13C2.6* (now known as *gldi-2)* and the tetraspanin *tsp-21* were enriched in cells not contacting AVK (Figure 7A). Notably, both HMR-1 and SYG-1 have documented binding interactions with proteins whose transcripts are expressed in AVK (*hmr-1* and *sax-3*, respectively), thus suggesting that these molecules may promote the membrane contacts of AVK.

The 62 significant cases of gene enrichment related to cell membrane contact involved 42 genes across 19 neurons (Figure 7B). In extreme cases, a gene was only detected in either the contacted or uncontacted neurons, such as with the Ephrin *vab-2* and the RIP neurons (Figure 7B). None of the 18 cells that contact RIP express *vab-2*, whereas 40% of the non-contacting cells do. Notably, RIP expressed transcripts for the Ephrin receptor *vab-1* and the Ephrin interacting protein *wrk-1,* which has been shown to promote mid-line repulsion in the developing *C. elegans* ventral nerve cord^126^. The secreted semaphorin *mab-20* was the most frequently enriched gene, showing significant enrichment in the contacted cells of six neurons, and enrichment in non-contacted cells in one additional neuron, all of which express transcripts of at least one *mab-20* interacting protein (Supplemental Table 17). These results suggest that Ephrin and semaphorin signaling may regulate the membrane contacts of individual neurons in the *C. elegans* nerve ring. This analysis is well-suited to identify individual molecules that regulate membrane contacts of a given neuron with many potential other neurons. It is plausible that a given neuron establishes membrane contact with other cells through combinatorial interactions involving multiple proteins or via distinct, individualized molecular pathways. We did identify cases with high log fold differences which do not pass significance because a transcript is detected only in one or two contacted cells (Figure 7B), which could possibly point to a role in individual interactions.

We then applied this same analysis to the L4 nerve ring to determine to what extent enrichment in contacted or non-contacted cells is maintained across larval development. Unexpectedly, while we detected 150 cases of enrichment at L4, only seven of these cases were also significantly enriched at the L1 stage (Figure 7C). Prior work has shown that distinct molecular mechanisms can mediate the maintenance of neuron and neurite placement and neuronal architecture in the *C. elegans* nerve cord and nerve ring^127–129^. The distinct patterns of gene enrichment in contacted vs non-contacted cells at L1 and L4 may reflect differences in genes involved in establishing vs maintaining membrane contact in the nerve ring. Overall, these data provide testable hypotheses for cell adhesion molecules that may underlie membrane contacts within the nerve ring.

### Identifying determinants of connectivity from a neuron-specific transcriptome

Electron micrograph reconstructions of the nerve ring at L1 revealed that a neuron is synaptically connected to only a small fraction of cells with which it has membrane contact, sending synaptic output to 12% (median) of contacted neurons and receiving presynaptic input from 9% (median) of contacted cells^6^ (Figure 7D). Previous work has shown that while the amount of membrane contact between nerve ring neurons in *C. elegans* is predictive of synaptic connectivity, it cannot predict all connections^130^. We reasoned that the molecular mechanisms either promoting or inhibiting the formation of synapses between neurons may be distinct from those driving membrane contact (Figure 7E). We therefore sought to identify transcripts of cell surface molecules and secreted proteins that are differentially expressed in synaptically connected neurons compared to neurons with only membrane contact. We restricted this analysis to the nerve ring of a late L1 larva for which EM reconstruction has described both membrane contacts and synapses^6^. We considered input and output connections separately and limited our analysis to neurons with ≥ 3 synaptic inputs or outputs.

### Detecting molecular determinants of postsynaptic connectivity

The sublateral motor neuron class SMB provides an example of our analysis for a neuron and its postsynaptic partners (Figure 7F–H). In the L1 nerve ring, the four SMB neurons have membrane contact with 67 neuron types but make presynaptic connections to only five of these classes (Figure 7G–H)^6,26,108^. We detected significant enrichment of seven genes in SMB-adjacent, non-synaptic neurons compared to SMB-postsynaptic partners (Figure 7H, Supplemental Table 16). Four of the seven genes, including the semaphorin receptor *plx-2* and ephrin *vab-2,* have known binding partners expressed in SMB (*mab-20* and *vab-1,* respectively). These data are consistent with the established roles of semaphorins and ephrins in axonal repulsion and inhibition of cell adhesion during *C. elegans* embryonic development^131–135^ and suggest that *mab-20/plx-2* and/or *vab-2/vab-1* signaling may antagonize the formation of postsynaptic connections in the nerve ring.

In another example from our analysis, SAA neurons had the highest number of enriched genes (16), with 14 enriched in non-synaptic, adjacent cells, and two transcripts enriched in synaptically connected cells (Figure 7I). Five of these cases feature genes with known binding partners including the Ig-domain IgLON molecules *rig-5* in SAA and *zig-8* enriched in SAA postsynaptic partners. This finding is notable, as RIG-5 and ZIG-8 are known to specify connections between selected classes of interneurons and motor neurons in the ventral nerve cord^136^. In addition, closely related IgLONs in *Drosophila melanogaster*, DIPs and Dprs, promote selective adhesion in the developing nervous system^137–140^. These data validate our computational approach for identifying molecular determinants of connectivity.

Overall, we identified 75 cases of genes with enriched patterns based on synaptic connectivity in a given neuron (Figure 7J), featuring 44 genes among 22 neurons. Forty-three percent of these instances involved genes with known binding partners expressed in a given neuron. Only five of the 75 cases, including *zig-8* shown in SAA outputs, were enriched in synaptically connected neurons compared to adjacent cells. The other synaptically-enriched cases were: *hpo-30* in synaptic contacts of the BAG neurons, M03F4.6 in synaptic contacts of the IL2 L/R pair, and *syg-1* in the postsynaptic contacts of SAA and RME D/V. The remaining 70 cases were enriched in non-synaptic adjacent cells. The higher number of genes significantly enriched in adjacent cells may, in part, be due to the larger number of neurons in the adjacent category compared to the synaptically-connected category, which influences the power of the statistical tests. However, our findings also suggest the possibility that multiple signaling pathways may actively prevent synapse formation in the nerve ring.

### Detecting molecular determinants of presynaptic connectivity

The analysis of each neuron and its presynaptic inputs in the L1 revealed 247 instances of significant gene expression enrichment in a presynaptic partner or solely in adjacent cells, featuring 94 genes across 29 neuron classes (Supplemental Figure 15A, Supplemental Table 19). All but one of these cases involved genes enriched in adjacent, non-synaptic partners, with only one instance involving a gene enriched in synaptic partners: *wrk-1* enriched in the synaptic partners of RMH relative to solely adjacent neurons. AIY neurons (Supplemental Figure 15B–C) provide an example of genes enriched in non-synaptic, adjacent cells (Supplemental Figure 15D). Two observations from this analysis support the idea that individual neurons utilize distinct molecules for synaptic partner recognition: 1) In addition to its enrichment in presynaptic inputs to RMH, *wrk-1* is enriched in adjacent, non-synaptic partners of four neurons: DVA, URX, RIG and SIA; 2) The 90 genes which showed enrichment in adjacent, non-synaptic partners were enriched in the adjacent cells of a median of two neuron classes. The genes showing the broadest enrichment in adjacent cells encode the semaphorin *mab-20* (9 classes), the receptor tyrosine phosphatase *clr-1* (8 classes), the glutamate receptor regulatory protein *neto-1* (7 classes), the Ig-domain containing protein *oig-5* (7 classes), and the neuronal GPI-anchored IgCAM protein *rig-6* (7 classes).

We note that this analysis is well-suited to identify genes expressed in many neurons in either the synaptically-connected or adjacent, non-synaptic categories. However, if a molecule had restricted expression to just a subset of connections for a given neuron and was instrumental in the establishment of those connections, it would not pass the significance threshold used in this analysis. For example, several genes are expressed in just one or two of the 11 presynaptic inputs to AIA and in none of its non-synaptic, adjacent neurons (Supplemental Figure 15E). Such cases can be identified by high log fold changes (Supplemental Tables 16, 17) and provide attractive hypotheses for molecules that may drive synaptic specificity in individual neuronal circuits.

EM reconstructions of the *C. elegans* nerve ring across larval stages show that connections between neurons change over development^6^. The majority (80%) of synaptic alternations in the nerve ring during larval development involved the strengthening of existing connections. Only 20% of synapses corresponded to new connections^6^. These findings point to potential mechanisms that sustain the specificity of synaptic partners throughout development in addition to pathways that specify initial connections earlier in development. We therefore sought to compare patterns of gene expression enrichment among synaptic or adjacent partners between the L1 and L4 stages, which may reflect distinct molecular components defining the establishment vs maintenance of neuron-specific synapses^129,141–143^. At L4, we identified 36 instances of significant enrichment in the comparison of postsynaptic outputs vs adjacent-only neurons and 134 significant cases of enrichment in presynaptic inputs vs adjacent-only neurons (Supplemental Tables 15, 16). A limited number of instances showed significant enrichment at both ages (Figure 7K, Supplemental Figure 15F). This observation suggests that distinct molecular mechanisms may underlie the initial patterning of synapses (i.e., prior to and during the L1 stage) and its subsequent maintenance (during the L4 stage). Altogether, this analysis reveals patterns of gene expression based on neuronal connectivity in the nerve ring and provides concrete hypotheses for future experiments to empirically test candidate molecular determinants of membrane contact and synaptic partner specificity.

## DISCUSSION

We have generated scRNA-seq profiles of the entire *C. elegans* nervous system in the first larval stage including post-embryonic neuronal lineages and newborn post-embryonic neurons. These data complement scRNA-seq profiles from embryonic and later larval and adult stages^16,17,24,25,144,145^ and provide a rich resource for understanding neuronal differentiation and maturation throughout development.

The profiles of post-embryonic neurogenic lineages, including the entire P lineage, should allow for mechanistic studies of cell fate specification and motor neuron development. We also identified thousands of differentially expressed genes within post-mitotic neurons between the embryonic and L1 stages and between the L1 and L4 larval stages. Our findings complement recent work characterizing temporal changes in the *C. elegans* nervous system using bulk RNA-sequencing of pooled neurons^12^. This pooled approach and subsequent validation with reporter strains revealed extensive changes in gene expression across development, driven in part by a genetic pathway involving the heterochronic genes *lin-4* and its negatively regulated target *lin-14*. In this work, we assign temporal changes in gene expression to at the resolution of single neuron to reveal thousands of additional differentially expressed genes. Many of these genes could have been missed using the pooled approach due to quantitative changes in a small number of neurons or to changes in opposing directions in different neuron classes. Notably, the increased resolution of our scRNA-seq dataset led to the identification of potential transcriptional regulators of cellular maturation at the level of single neurons.

Our data show that neuropeptide encoding genes and neuropeptide receptors are highly differentially expressed across development throughout the nervous system. This finding is consistent with results from bulk RNA sequencing and imaging of individual neuropeptide reporters^12^. Our nervous-system wide analysis of the neuropeptide signaling network at L1 and comparison to the L4 reveals both conserved connections and novel stage-specific patterns of signaling. Strikingly, neuropeptide expression is also highly sexually dimorphic within the *C. elegans* nervous system^146,147^ as well as highly divergent between *Caenorhabditis* species^18^. Collectively, these data identify neuropeptidergic signaling as a highly dynamic feature of nervous system function across age, sex and evolution.

Lastly, we combined expression data with larval nerve ring connectomic data to identify potential secreted and cell surface proteins with roles in the establishment and maintenance of synaptic connections. This analysis detected instances of gene expression negatively correlated with synaptic connectivity, suggesting possible widespread roles for anti-synaptic signaling in the *C. elegans* nerve ring.

The data can be explored at the following CengenApps: L1: https://cengen.shinyapps.io/L1app; updated L4 data: https://cengen.shinyapps.io/L4app. Raw fastq files for all the L1 data are available at Gene Expression Omnibus (https://www.ncbi.nlm.nih.gov/geo) under the accession number: GSE310667. We expect that these data will inspire future studies of genes, neurons and circuits, and will allow for mechanistic insight into neuronal development and maturation across an entire nervous system.

### Limitations of the study

While we captured every embryonically derived neuron class in L1 and nearly all the postembryonically derived neurons, we did not identify two neuron classes that are born in late L1: DVB and PDB. In addition, not all neuron classes are equally represented, and the under-represented classes likely have incomplete detection of expressed transcripts. For scRNA-Seq, we used a 3’ gene expression assay, which fails to capture non-polyadenylated transcripts and many non-coding RNAs.

## RESOURCE AVAILABILITY

### Lead contact

Requests for further information and reagents should be directed to the lead contact, Seth Taylor (seth_taylor@byu.edu).

### Materials availability

The strains used in this study can be obtained from the *Caenorhabditis Genetics Center* or upon request from the lead contact.

### Data and code availability

The raw data are available at GEO under the accession number GSE310667. The data can be explored interactively at the L1 CengenApp (https://cengen.shinyapps.io/L1app) or the L4 CengenApp (https://cengen.shinyapps.io/L4app). Examples of the analysis code can be found at github: https://github.com/cengenproject or upon request from the lead contact.

## STAR Methods

### Worm Maintenance

Worms were cultured at 20° C unless otherwise described and maintained on NGM agar plates seeded with OP50–1 bacteria or on 8P agar plates seeded with NA22 bacteria.

### Strain List

See Supplemental Table 1.

### Mesh Synchronization

To obtain synchronized cultures of mid-late L1 worms, we scaled up worm cultures by chunking ¼ of a 60 mm plate of recently starved worms onto a 100 mm plate seeded with OP50–1. After culturing for 2–3 days at 20 C, 1/5 of each plate was chunked onto 8P nutrient agar 150 mm plates seeded with *E. coli* strain NA22. Worms were cultured at 20 C for 3–4 days. Approximately ten 150 mm plates were used for each strain to be sorted (see below). Five 150 mm plates were used for sorting controls. We scraped worms and eggs off 150 mm plates with a cell scraper and pelleted by centrifugation at 150 rcf for 2.5 minutes. Embryos were isolated by hypochlorite treatment of the pellet and floatation in 30% sucrose. Embryos were washed 2x in M9 and placed on a nylon mesh grid with 10-micron or 18-micron pores. Embryos will not pass through the pores of the mesh, but hatched L1 larvae are able to crawl through. The mesh was placed on M9 containing NA22 bacteria, and embryos were allowed to hatch at room temperature. Larvae collected in the first 1.5 hours after completion of hypochlorite treatment were discarded, and the mesh grid was placed over a fresh collection media of M9 with NA22 bacteria for 2–4 hours. Hatched L1s were pelleted by centrifugation and plated onto fresh 8P × 150 mm plates with NA22 and incubated at 20° C for 12–17 hours. This timetable yields larval populations synchronized to within 14–20 hours after hatching. Where possible, we estimated the developmental stage of cultures by counting fluorescent protein expression in post-embryonic neurons that are born during the first larval stage (L1). For example, with strain OH10689, which features rab-3::2xNLS-TagRFP in all neuronal nuclei, we counted the number of RFP+ neurons in the ventral nerve cord. For strains without fluorescent labeling of easily identifiable post-embryonic neurons, we cultured worms for similar durations of time post hatching. Estimates of developmental staging are found in Supplemental Table 2.

### Dissociation

Single cell suspensions were obtained with modifications from prior work^16,23,148–150^ as follows. Synchronized larvae were collected and separated from bacteria by washing twice with ice-cold M9 and centrifuging at 150 rcf for 2.5 minutes. Washed larvae were transferred to a 1.6 mL centrifuge tube and pelleted at 16,000 rcf for 1 minute. 100–250 μL pellets of packed worms were treated with 500 μL of SDS-DTT solution (20 mM HEPES, 0.25% SDS, 200 mM DTT, 3% sucrose, pH 8.0) for 2 minutes. Following SDS-DTT treatment, worms were washed five times by diluting with 1 mL egg buffer and pelleting at 16,000 rcf for 30 seconds. Worms were then incubated in pronase (15 mg/mL, Sigma-Aldrich P8811, diluted in egg buffer) for 23 minutes on a rocking nutator. During the pronase incubation, worms were mechanically disrupted by pipetting through a P1000 pipette tip for four sets of 80 repetitions. The status of dissociation was monitored under a fluorescence dissecting microscope at 5-minute intervals. The pronase digestion was stopped by adding 750 μL L-15 media supplemented with 10% fetal bovine serum (L-15-10), and cells were pelleted by centrifuging at 530 rcf for 5 minutes at 4° C. The pellet was resuspended in L-15-10, and single cells were separated from whole worms and debris by centrifuging at 150 rcf for 2 minutes at 4° C. The supernatant was passed through a 35-micron filter cap into a 5 mL round bottom tube. The pellet was resuspended a second time in L-15-10, spun at 150 rcf for 2 minutes at 4° C, and the resulting supernatant was added to the collection tube.

### Fluorescent-Activated Cell Sorting

Fluorescence Activated Cell Sorting (FACS) was performed on a BD FACSAria^™^III Cell Sorter equipped with 70-micron diameter nozzles^16,23,28^. DAPI was added to the sample (final concentration of 1 μg/mL) to label dead and dying cells. We used a variety of fluorescent reporter strains to label subsets of neuronal cells (Supplemental Table 2). For example, we used an *acr-2::GFP* reporter (strain CZ631) to target ventral nerve cord cholinergic motor neurons and an *unc-47::GFP* reporter (strain EG1285) to target GABAergic neurons. N2 worms lacking fluorescence and single-color controls (for intersectional labeling strategies and dual-color sorts) were used to set gates to exclude auto-fluorescent cells and to compensate for spillover between fluorescent channels. In three experiments, single-cell suspensions from separate strains were combined prior to FACS: (1) NC3583, NC2957; (2) OH16003, EG1285, ZM5488 and *uIs152*; (3) CZ631, NC3523, PS3504. Cells were sorted under the “4-way purity” mask. Cells expressing the desired fluorescent proteins were collected into 400 μL of chilled (4° C) L-15 media containing 33% FBS (L-15-33) and stored on ice. We concentrated the collected cells by centrifuging at 500 rcf for 5 minutes at 4° C. We removed all but ~30–50 μL of the supernatant, resuspended the cells and counted fluorescent cells on a hemocytometer. Cell suspensions loaded into the 10X Chromium Controller ranged in concentration from 350–1000 cells/uL.

From one preparation of sorted cells, we generated both a live-cell sample and a methanol-fixed sample after sorting. This preparation contained strains NC3583 and NC2957. The live cell sample was prepared as described above. After concentrating the sample, we had ~117 μL of sample at ~1000 cells/μL. We submitted 22 μL for the live-cell sample. The remaining amount was fixed in methanol by adding 1 mL of ice-cold methanol drop-wise while gently vortexing the cells, followed by an additional 3 mL of ice-cold methanol. The cells were stored at −20° C for 22 days. Fixed cells were pelleted by centrifugation at 1000 rcf for 12 min at 4° C. We removed 4.05 mL of the supernatant. The fixed cells were suspended in 400 μL of egg buffer with 1% BSA and 2.5 U/μL RNAse inhibitor (NEB M0314L). Resuspended cells were concentrated by centrifuging at 800 rcf for 12 min at 4° C. 350 μL of the supernatant was removed and cells were gently resuspended. An aliquot was taken, DAPI was added (to concentration of 1 μg/mL), cells were counted on a hemocytometer based on the DAPI signal and then loaded onto the 10X Chromium Controller.

### Single-cell RNA-Sequencing

Each sample (targeting 10,000 cells per sample) was processed for single cell 3’RNA sequencing utilizing the 10X Chromium system. Libraries were prepared using P/N 1000121 following the manufacturer’s protocol. The libraries were sequenced using the Illumina NovaSeq 6000 with 150 bp paired end reads. Real-Time Analysis software (RTA,version 2.4.11; Illumina) was used for base calling and analysis was completed using 10X Genomics Cell Ranger software (v6.0.0), and a custom reference genome based on Wormbase release WS273^151^ with extended 3’ UTR sequences^16^.

### Data Analysis

Data analysis downstream of CellRanger was performed in R. We used the DropletUtils R package (version 1.6.1) function EmptyDrops^152^ to distinguish cells from empty droplets. We used a threshold of 50 UMIs for all samples except sample 8672-ST, for which we set the threshold at 90 UMI due to apparently higher background contamination, as evidenced by a pronounced plateau when plotting the number of UMIs in each barcode. Contamination from ambient RNA was estimated and corrected using the SoupX R package (version 1.6.1)^153^. The parameters and genes used to estimate the amount of contamination for each sample are listed in Supplementary Table 2. We performed quality control with the scater package (1.24)^154^, using the percentage of UMIs mapping to the mitochondrial transcripts nduo-1, nduo-2, nduo-3, nduo-4, nduo-5, nduo-6, ctc-1, ctc-2, ctc-3, ndfl-4, atp-6, ctb-1, MTCE.7, and MTCE.33. The individual datasets for each sample were then combined and further processed using the monocle3 package (version 1.2.9)^17,24,155,156^. Genes detected in fewer than 5 cells were removed, and data were log-normalized. We performed dimensionality reduction using PCA (using 100 PCs, based on an elbow plot showing the variance explained by each principle component), followed by UMAP (with umap.min_dist = 0.3, umap.n_neighbors = 75). Cells were clustered on UMAP coordinates using the default leiden algorithm (res = 1e-3). Clusters were assigned cell type identities based on the expression of known cell-type specific genes^16,27,28,45^.

### Reanalyzing published embryonic data

We re-analyzed single-cell data from *C. elegans* embryonic cells to obviate potential artifactual results which might arise from differences in analysis pipelines^24^. We downloaded fastq files from the SRA archives for GSE126954. This series includes data from 15 separate 10X data sets. We processed these files through CellRanger (v8.0.0), using the same custom WS273 reference genome used for the L1 data. A different reference genome was used in the original analysis of these data. We then processed the raw feature × barcode matrices using EmptyDrops. In the original analysis, the authors initially used stringent UMI count thresholds to distinguish cells from empty droplets. We reasoned that this approach may have excluded many neurons, including possible additional classes not originally annotated. From the processed embryonic data available at https://cello.shinyapps.io/celegans/, we extracted the unique cell barcode IDs and corresponding metadata from each individual experiment. We generated new filtered feature × barcode matrices that retained all the same cell barcodes from the original analysis as well as cell barcodes detected with our EmptyDrops method. We then used SoupX, scater and monocle3 to further process the data. Details for SoupX contamination correction of embryonic datasets can be found in Supplemental Table 9.

We then merged the individual datasets and performed dimensionality reduction. We used pan-neuronal markers *sbt-1* and *egl-21*^16,17,24^ to identify the post-mitotic neuronal populations. We subset clusters containing neurons and performed additional rounds of dimensionality reduction, using a range of principal components (18–50) and varying the UMAP parameters (umap.min_dist from 0.1 to 0.3; umap.n_neighbors from 25 to 50) to iteratively separate groups of cells. In each round of subsetting, cells were clustered and cluster markers were identified using the monocle3 “top_markers” function. Cluster markers were compared to expression of L4 neuronal data using the CeNGENApp and to expression atlases of homeodomain transcription factors^45,157^ to annotate cell identity. In addition to clustering with previously identified embryonic cell types, newly detected embryonic neurons also appeared as novel clusters in the UMAP. Using gene expression patterns from previously published single-cell datasets and the literature, we annotated most of these new clusters. In total, we annotated 92 neuronal classes. Of these, 89 types had a matching type in the L1 data. The three classes that did not match were instances in which subclasses were detected in L1 that could not be unambiguously distinguished in the embryonic data (AWC, RMD, and RME in embryo; AWC^ON/^AWC^OFF^, RMD LR/RMD DV, and RME LR/DV in L1). Our reanalysis identified 28 additional neuronal classes that were not conclusively annotated in the original embryonic data.

### Reannotation of L4 single-cell data

During annotation of the L1 single cell data, we identified subsets of cells for several neuron types that showed high expression of genes involved in unfolded protein responses (UPR). We annotated these separately from cells without high levels of UPR-related genes and excluded them from further analysis to avoid expression artifacts likely resulting from the dissociation procedure. For direct comparisons with the new L1 data, we also removed cells with high UPR responses from the previously obtained L4 data set^16^. Additionally, both the current L1 single-cell dataset and recently published data from adult animals^28^ resolved some cell types that were not originally identified in the L4 data set. Using new marker genes described in the L1 and adult datasets, we were able to separate the DD and VD neurons and identify a distinct VA1 population in the L4 data.

### Differential Gene Expression

Differential gene expression analyses were completed using the Wilcoxon rank-sum test in Seurat (v4.4.0 for P lineage comparisons; v5.3.0 for L1 vs embryo, L1 vs L4)^158–161^. For the L1 to L4 comparisons, monocle3 cell_data_set objects containing the L1 and L4 neuronal single-cell datasets were combined into one object. This object was then converted into a Seurat object. 121 neuronal cell types were present in both stages. For each cell type, the Seurat object was subset to include only that cell type and the data were then normalized using the Seurat function NormalizeData with default parameters. The FindMarkers function was used to identify differentially expressed genes between L1 and L4 cells. The following parameters were used: ident.1 = “L4,” ident.2 = “L1,” logfc.threshold = 0, min.pct = 0. With these parameters, positive average log2 fold change values indicate higher expression in L4, whereas negative log2 fold change values indicate higher expression in L1. The results from all 121 separate comparisons were compiled into one table. We filtered the results to retain comparisons based on four criteria: 1) an adjusted p-value of < 0.05; 2) an absolute average log2 fold change of >= 1; 3) the gene must have been detected in > 10% of the cells in the higher-expressing stage; 4) the gene must have been detected in > 5 cells in the higher-expressing stage. Additionally, we identified 23 genes whose quantitative expression is unreliable in either the L1 or L4 data as their genomic loci were overexpressed in the transgenic reporter strains (Supplemental Table 8). These genes were also removed from the differential expression results.

We used the pheatmap R package^162^ to visualize differential expression results. We removed genes that did not show any significant differential expression, resulting in 5810 genes. For visualization, we show the average log2 fold change values for these genes across all neurons. The embryo to L1 differential gene expression analysis was performed in the same way between 89 neuron classes present in both datasets. For this comparison, negative log2 fold change values indicate higher expression in the embryo, and positive values indicate higher expression in L1.

We used the following criteria to identify genes broadly downregulated in early postmitotic neurons from both comparisons: genes had to be significantly differentially expressed in more than 10 neuron classes between L1 and L4, with at least 75% of those cases higher at L1 and they had to also be differentially expressed in > 40 neuron classes between the embryo and L1 with higher expression in the embryo in at least 75% of the cases. Forty-one genes met these criteria. We used linear regression including functional categories or neuron birthtimes and the number of cells present at each age as covariates to account for sampling differences.

For each differential expression comparison within the P-lineage, the Seurat object was subset to include only the two cell types to be compared. The FindMarkers function was used to identify differentially expressed genes between the two cell types, with parameters min.logFC.threshold = 0 and min.pct = 0. For parent-daughter cell comparisons, ident.1 was set to the parent cell and ident.2 was set to the daughter cell, so positive log2FC changes indicate higher expression in the parent. For sister-sister cell comparisons, ident.1 was set to the anterior sister and ident.2 was set to the posterior sister, so positive log2FC changes indicate higher expression in the anterior sister. The results were filtered on 4 criteria: (1) adjusted_p_value < 0.05; (2) log2FC > 1; (3) gene detected in 10% cells in the higher expressing cell type; (4) and gene detected in >5 cells in the higher expressing cell type.

For comparisons involving VD, we combined VD, VD2, VD12, and VD13 cells. For comparisons involving the P0.aa-AVF-VB02 and P1.aaa-AVF-VB01 sub-lineages, we combined VB01 and VB02 cells. We used the pheatmap R package for visualization of differential expression results. For Figure 3K, we filtered the P-lineage differential expression for transcription factors differentially expressed between at least one pair of directly related cell types under the above parameters. We show single-cell scaled TPM values for each gene across a subset of P-lineage cell types.

### Gene Set Enrichment Analysis

Gene set enrichment was performed in R using Wormcat 2.0 annotations^74^. The whole genome annotations (version from Nov 11, 2021) were downloaded from www.wormcat.com and the wormcat R package was installed from GitHub. The background gene set for genes from the embryo vs L1 comparison consisted of the 17626 genes detected (at least one UMI in at least six cells) in the merged embryo-L1 dataset. The background gene set for genes from the L1 vs L4 comparison consisted of the 18330 genes detected in the merged L1-L4 neuronal datasets. We used Bonferroni corrected p-values and calculated a fold-enrichment from the background set using the formula^144^:

Foldenrichment=(Numberofqueriedgenesincategory/Numberofgenesinqueryset)/(Numberofbackgroundgenesincategory/Totalnumberofgenesinbackgroundset).

### Gene Regulatory Network Analysis

We used the *Cel*EsT gene regulatory network (GRN)^83^ to estimate transcription factor activity from the differential expression analyses between the embryo and L1 stages and between the L1 and L4 stages. For each neuron in each age comparison, the average log2 fold-change for all genes was used as input with the *Cel*EsT GRN, which contains data for 487 transcription factors, for the decouple function in the decoupleR R package^85^. We used the multivariate linear model (‘mlm’). We calculated Benjamini-Hochberg corrected p-values to control for multiple comparisons within each neuron. We retained only those transcription factors with adjusted p-values < 0.05. We also filtered our results to retain TFs that were detected in the neuron of interest in at least one of the ages of the comparison.

### Thresholding

To distinguish true gene expression from noise, we used a thresholding procedure^16^ on pseudobulk aggregated data based on the proportion of cells in each neuron type in which a gene was detected. We performed this thresholding on the reannotated L4 data and the L1 dataset separately. We first calculated aggregate statistics for each neuron type, including the proportion of cells expressing each gene and a normalized TPM expression value^24^. This TPM value is calculated by normalizing each cell’s UMI counts by a size factor (the Monocle software package generates this as the cell’s total UMI count divided by the geometric mean of all cells’ total UMI counts), taking the mean normalized expression of each gene across the single cells corresponding to a cell type, and rescaling the resulting values to sum to 1,000,000^24^. We set initial filters based on proportions to retain ubiquitously expressed genes and to remove genes with very low to no expression across all neurons. In the L4 dataset, 183 genes were detected in >1% of all neuron classes and were retained, whereas 4109 genes were detected in <2% of every neuron class and were removed. In the L1 dataset, 474 genes were found in >1% of the cells in every neuron class and were retained, and 5345 genes which were detected in <2% of every neuron class were considered not expressed and were removed. No genes were detected in > 1% but < 2% of every neuron class in either stage.

The remaining genes were thresholded using a dynamic thresholding procedure in which each gene was considered individually. For each gene, we determined the cell type which had the highest proportion of cells expressing that gene. Thresholds were set as fractions of the highest proportion for each gene. We compared each threshold to a ground truth dataset^16^ consisting of 160 genes with known expression patterns across the nervous system. We generated an L1 ground truth dataset (Supplemental Table 14) which differed slightly from the previously used L4 ground truth dataset due to developmental changes in innexin expression as described^163^. The L4 ground truth was the same as previously used. For a wide range of threshold values (272 possible thresholds, ranging from 0 – 1.0), we generated 5,000 stratified bootstraps of the ground truth genes using the R package boot^164,165^, and computed true positive (TPR), false positive (FPR) and false discovery rates (FDR). We estimated 95% confidence intervals with the adjusted percentile (BCa) method.

We performed this thresholding for the L1 dataset using the L1 ground truth and generated new thresholded datasets for the reannotated L4 data using the L4 ground truth. For the L4 data, we used threshold values of 0.02, 0.04, 0.09 and 0.15 as previously described^16^. We selected four threshold levels of varying stringency for the L1 data, at 0.025, 0.04, 0.08, and 0.13 *of the highest proportion* of cells in which a gene was detected. These thresholds were selected to match the TPR, FPR, and FDR values from the L4 data (Supplemental Table 15).

At each dynamic threshold level, for each gene the TPM value was set to 0 for each neuron type in which the proportion of cells expressing the gene was below the dynamic threshold for that gene. For example, in the L1 data, the VA1 neuron cluster had highest proportion of cells with transcripts for the transcription factor *unc-4*, at 90.7% of the cells, giving proportion thresholds of 0.02265, 0.03624, 0.07248, and 0.11778. For the posterior Hox gene *php-3*, the neuron class PHC had the highest proportion of positive cells, with 42.3% of PHC cells having *php-3* transcripts. Proportion thresholds for *php-3* were 0.0106 (i.e., 0.025 * 0.423), 0.01692, 0.03384 and 0.05499. Thus, at the lowest stringency (threshold 1), the *unc-4* TPM value was set to 0 in all cell types in which it detected in fewer than 2.265% of the cells, whereas at the highest stringency (threshold 4), *unc-4* TPM value was set to 0 in all neuron types in which it was detected in fewer than 11.778% of cells. In the case of *php-3*, at threshold 1, all neuron types in which it was detected in fewer than 1.06% of the cells were set to 0, and at threshold 4, all neuron types in which *php-3* was in fewer than 5.499% of cells were set to 0. For all neuron classes in which the proportion of cells expressing a gene was above the threshold, the continuous TPM values were retained.

Thresholds and stats for each age:

L4 data

**Table:** 

Threshold	Fraction of highest proportion	TPR	FPR	FDR
1	0.02	0.853	0.118	0.197
2	0.04	0.810	0.0880	0.145
3	0.09	0.695	0.0458	0.105
4	0.15	0.578	0.0301	0.085

L1 data

**Table:** 

Threshold	Fraction of highest proportion	TPR	FPR	FDR
1	0.025	0.853	0.120	0.202
2	0.04	0.807	0.092	0.170
3	0.08	0.689	0.055	0.127
4	0.13	0.577	0.037	0.103

We also performed thresholding using the same procedure for the newly annotated embryonic data. We used the same thresholds as for the L1 data, but as we do not have an equivalent ground truth dataset for the embryonic nervous system, we were unable to generate TPR, FPR and FDR rates.

### L1 stability and Jaccard similarity

We calculated the stability of gene expression from L1 to L4 using binarized expression data from threshold 2. We assessed the stability of expression for each gene as follows:

NumberofneuronsinwhichgeneisdetectedatbothL1andL4/(NumberofneuronsinwhichgeneisdetectedatbothL1andL4+NumberofneuronsinwhichgeneisdetectedonlyatL1)

To account for the appearance of expression at L4 as well, we also calculated a Jaccard similarity index for each gene:

NumberofneuronsinwhichgeneisdetectedatbothL1andL4/Numberofneuronsinwhichgeneisdetectedatanystage

Genes were grouped into functional gene families based on the literature, known function and sequence homology^121^.

### Microscopy

For confocal imaging, worms were mounted on slides containing agarose pads (2–5%) and immobilized with levamisole (17 mM). Images were acquired on a Nikon A1R or Olympus FV1000 laser scanning confocal microscope, with 40X or 60X oil immersion objectives.

#### Validation of differential expression between L1 and L4

For imaging analysis of neuropeptide reporters in the L1 and L4 stage, well-fed, gravid adults were treated with a hypochlorite solution. Between 20 to 30 eggs were transferred to a standard OP50 seeded plate. Animals were bleached and eggs seeded 19h before imaging for L1 stage and 48h before imaging for the L4 stage. In some experiments, L1 and L4 worms identified by morphology and picked off mixed stage, well-fed plates. Worms were immobilized with 100 mM sodium azide (NaN_3_) and placed on 5% agarose pads on glass slides. Imaging was conducted at the L1 or L4 stage. Images were captured using a wide-filed microscope (Axio Imager Z2) at 40X magnification. Image processing and the fluorescence intensity measurements were carried out using Fiji^166^. We combined CRISPR/Cas9 engineered reporter alleles for neuropeptide genes with the NeuroPAL landmark strain *(otIs669)* to determine in which neuron types they are expressed^167^. Neuron types were identified by the NeuroPAL colour code and the location of their nuclei compared to other neurons. Comprehensive guides for identifying neurons using NeuroPAL are available at https://www.hobertlab.org/neuropal/. In cases in which NeuroPAL was not used for neural ID, the expression of the reporter in a neuron was determined by assessing the presence of “speckles” in the cell of interest^168^. Anatomical position within a specific ganglion was used to further specify the final ID.

#### In vivo quantification of ribosomal protein expression

Ribosomal protein encoding genes *rps-4, rpl-7A* and *rpl-25.1* were tagged endogenously with *C. elegans* codon optimized mEosEM at their C-terminus by CRISPR/Cas9 genome editing as described^169^. Briefly, single stranded repair templates encoding mEosEM flanked with 40 bp homology arms were co-injected with pre-complexed Cas9 RNP targeting the respective locus. Worms at the L1 or L4 stage were immobilized with 50mM sodium azide on 5% agarose pads and imaged on a Zeiss LSM980 laser scanning confocal microscope (40x objective) with the 488nm laser and full spectral range of the GaAsP-PMT detector at 800V for fluorescence and transmitted light PMT detector for Nomarski co-imaging. Regions corresponding to indicated cell types were outlined and measurements were restricted to cytoplasmic areas using the “threshold” function in Fiji before pixel intensity was quantified.

### Maturation index

To calculate a maturation index for cells within newly born individual cell types, for each post-mitotic cell type, we selected a UMAP coordinate pair at the branch of the progenitor cluster and the post-mitotic neural cells as a baseline. We then calculated both the Euclidean and Manhattan distances in UMAP space of each cell of that type from the baseline. We used the Euclidean distance as the maturation index. We generated Pearson correlations and corresponding p-values between the expression of each gene and the maturation index within each post-mitotic cell type. We kept only genes that had Bonferroni corrected p-values of < 0.05. For visualization, we ordered cells within a neuron class by maturation index and plotted the log2 normalized expression using the R package pheatmap. Genes were clustered by similarity using the “binary” distance measure the “ward.D2” clustering method.

### Neuropeptide Network analysis

#### Neuropeptide network spatial constraining

Neuropeptide signalling was locally thresholded to filter out connections between neurons that were anatomically distant from each other. A developmental-stage dependent matrix of anatomical proximity was constructed using electron microscopy data of the nervous system of the *C. elegans* L1 larvae (16h after hatching)^6,9,106^. These data were used to create a table of locations for each neuronal process, identifying 27 different neuronal process bundles in the *Caenorhabditis* nervous system as previously defined^26^. This classification was then used to filter out neuropeptidergic connections based on putative signalling ranges in the three species. The mid-range stringency thresholding used in this analysis allows connections between neurons with neuronal processes in the same anatomical area (~50μm distance): head (including pharynx and the ventral cord neurons that are in the ventral ganglion), midbody and tail.

#### Neuropeptide network construction

The neuropeptide networks were constructed as described in^106^ using biochemically validated interactions, and the L1 larvae expression data gathered in this article and for the 45 neuropeptide precursor genes (NPPs) and 47 GPCR receptors out of these validated pairs that are expressed in both L1 and L4 larvae. The adjacency matrix was built using a binary version of the expression data for the 291 neurons present at both developmental stages (L1 and L4 larvae).

For a given point *A*(i, j)^N^ and for a given neuropeptide receptor pair N the connection between two neurons is defined by *A*(*i*, *j*)^*N*^ = *NPP*(*i*, *j*)^*N*^ × *GPCR*(*i*, *j*)^*N*^. Each neuropeptide receptor interaction forms an individual binary network. To get the overall neuropeptide network we added each individual neuropeptide receptor network resulting in a weighted network where the weight indicates the number of neuropeptide receptor pairs that connect two nodes. For the across-development network analysis, we binarised all the connections of each developmental-stage network separately, then integrated the data into a single network reflecting the conservation pattern of connections between homologous neurons throughout development.

#### Degree

Edge counts and adjacency matrices were all computed using binary directed versions of the networks. These networks were used to compute degree using the Brain Connectivity Toolbox^170^ for MATLAB. Degree is the number of edges connected to a given node. In-degree is the number of incoming connections connected to a given node and out-degree is the number of outgoing connections.

#### Dimensionality reduction analysis

The dimensions of the neuropeptide network adjacency matrix were reduced using a combination of t-SNE and PCA methods, and clustering was performed based on the pattern of connections due to receptor expression. Individual clustering was performed for each developmental stage and a comparison between neuronal identities in each cluster per developmental stage was performed.

#### Quantification and statistical analysis

Statistical data is reported in the main text, figures, and tables as noted. Significance adheres to the common standard, after adjusting for multiple testing, of p < 0.05. The symbols *, **, ***, **** refer to p < 0.05, 0.01, 0.001, and 0.0001, respectively. Not significant is described as n.s. The n for each statistical test is described in each figure.

### Connectome comparison

#### Standardizing chemical synaptic and membrane contact matrices

The EM data of larval nerve rings^6^ provide resolution at the level of single individual cells (e.g., ASIL and ASIR). To match the resolution of the scRNA-seq expression data, we summed the number of synaptic connections and the physical membrane contacts of the neurons within each transcriptionally defined class (ASIL + ASIR for the ASI neuron). We did this independently for larval datasets from late L1 through adult (datasets 4 – 8 and series JSH and N2U in source publications)^6,124^. To account for the variability between animals, we focused on connections present in multiple datasets. For the L1 dataset, we used the summed synapse counts and membrane contact data from dataset 4 (~16 hours after birth, in late L1) but only kept those synapses and membrane contacts that were also present in dataset 5 (~23 hours after birth, L2 stage).

For the L4 comparison, we used the numbers from dataset 8 (~50 hours after birth) but only kept synapses that were also present in dataset 7, and both the JSH and N2U reconstructions^124^ and membrane contacts that were also present in the JSH and N2U series (membrane contact data are not available for dataset 7^6^).

We generated a list of 443 genes that encode proteins with extracellular domains from several published resources^118,119,121^. These include genes encoding transmembrane cell surface proteins, predicted GPI-anchored proteins and secreted proteins. 298 of these were detected in nerve ring neurons at L1. 313 of these were detected in nerve ring neurons in the updated L4 data. Binding data for extracellular domains was taken from Table S3 in Nawrocka, et al^118^ and from Wormbase and the Alliance for Genome Resources^171^. We used the SimpleMine tool in the Alliance for Genome Resources website to search for curated Protein-Protein interactions between the genes in our CAM list. We then used the Wormbase interaction data to identify publications supporting these interactions and manually curated interactions that included the extracellular domains to exclude binding of intracellular regions.

#### Membrane contact enrichment

For each neuron (‘tested neuron’) with contacts in the nerve ring (80 neurons at L1, 84 at L4), we separated all other neurons into two categories based on whether they had any membrane contact with the tested neuron. We performed unpaired two-sample Welch’s t-tests (assuming unequal variances) in R on the mean expression of each gene in contacted neurons versus the mean expression in non-contacted neurons. We used the threshold 2 TPM expression values for this analysis. We also generated a t-statistic representing the magnitude and direction of enrichment (positive values indicate enrichment in contacted cells, negative values indicate enrichment in non-contacted cells), p-value, the fold change of the means and log2 transformed fold changes as a measure of effect size. In calculating log2 fold changes, we added a value of ‘1e-5’ to the mean of each group to prevent undefined values when one mean was equal to zero. We generated adjusted p-values for multiple comparisons across genes in each neuron using the “p.adjust” function in R with the Benjamini-Hochberg method. We also determined for each gene whether any known binding partners were expressed in the tested neuron.

#### Synaptic partner gene enrichment

For each tested neuron, we split all other cells having membrane contact with the tested neuron into one of two categories: 1) synaptic partners or 2) non-synaptic, but adjacent cells. We performed this operation separately for presynaptic inputs and postsynaptic outputs for each tested neuron. We restricted this analysis to neurons that had at least three presynaptic inputs or postsynaptic outputs. We used the threshold 2 TPM normalized expression values for this analysis. We performed unpaired Welch’s t-tests on the mean expression of each gene in the synaptically-connected neurons versus the non-synaptic, adjacent neurons. We generated fold change, log2 transformed log fold change, t-statistics, p-values and Benjamini-Hochberg adjusted p-values for each tested gene/tested neuron combination. We then assessed whether a tested gene had any known binding partners in the tested neuron. We performed this analysis separately for the data from L1 and L4.

## Supplementary Material

Supplement 1 **Supplemental Figure 1. Single-cell RNA sequencing of L1 larvae**. A) Graphical representation of neuron-specific fluorescent reporter strains and developmental ages sampled for scRNA-Seq. The *rab-3*, *flp-7* and *ceh-34; unc-4* reporter strains were used for two experimental samples each. B) Schematic of mesh protocol used to generate large synchronous cultures (top) and use of FACS to enrich for targeted cell populations (bottom). C) UMAP showing the entire dataset of 161,562 cells, colored by cell type annotation. D) UMAP of all cells colored by expression of the pan-neural gene *sbt-1* marking post-mitotic neurons. E) UMAP of all cells colored by expression of the cell cycle gene *cdk-1* marking progenitor cells.

Supplement 2 **Supplemental Figure 2. Neuronal subclasses are present in the L1.** A) Sub-UMAP showing separation of neuronal subclasses for RMD, RME, IL2, ASE, and AWC. B) Sub-UMAP showing embryonic motor neurons that include the newborn RMH cluster, the G neuroblast, DD, SAB, and subclasses of the DA and DB motor neurons. C) Sub-UMAP showing expression of the DB class marker *vab-7*. D) Sub-UMAP showing expression of the DB2-specific marker *hlh-17*. E) Expression of the DA and SAB class marker *unc-4*. F) Expression of the tubulin *tba-9*, which is restricted to DA1 among DA neurons^28^.

Supplement 3 **Supplemental Figure 3. Annotation of VC neurons in L1.** L1 HSN and VC neurons express low levels of the pan-neural genes *egl-21* (A) and *snt-1* (B). C) The VC neuron cluster uniquely shows co-expression of the transcription factors *ceh-6* and *lin-39*. D) UMAP of *ham-2* expression, which is restricted to HSN and VC.

Supplement 4 **Supplemental Figure 4. Q and T lineage progenitors and neurons in L1.** A-E) Sub-UMAPs of the Q.pa lineage showing expression of A) the cell cycle gene *cdk-1* marking the Q.pa cluster B) *sbt-1* in post-mitotic neurons, C) *unc-86* in Q.pa, AVM and PVM, but with decreased expression in the SDQ cluster (top right), especially in cells further from the Q.pa cluster. D) Expression of *mec-3* is stable among all AVM and PVM neurons. E) Expression of *mec-17* increases in both AVM and PVM in cells with increasing UMAP distance from the Q.pa progenitor cluster. F) Jitter plot of *mec-17* expression (y-axis) by maturation index (x-axis) for AVM. Maroon line shows the smoothed mean expression using the loess method. G) Jitter plot as in F, but for *egl-46*. Note the decrease with maturation index. H) Heatmap of log2 expression of 530 genes that showed significant statistical correlations (Bonferroni-corrected p-value < 0.05) with the maturation index in AVM. The columns are cells ordered by increasing maturation index. Rows are genes, clustered by similarity. I) Heatmap as in H, but for 475 genes with statistically significant correlation with maturation index in PVM. J) Heatmap as in H, I, but for 395 genes with significant correlation with maturation index in SDQ. K) Jitter plots showing shared regulation of genes across early postmitotic development in AVM, PVM, and SDQ. The lysosomal enzyme cathepsin A, *ctsa-1.1* (left), shows shared downregulation with maturation in all three neurons, whereas *mec-2* showed shared upregulation with maturation (right). L) The neuropeptide encoding genes *flp-20*, *flp-8* and *flp-12* showed cell-type specific upregulation. M) PHATE plot of the T-lineage showing expression of *cdk-1*, which marks neuronal progenitors. N) PHATE plot of the T-lineage showing expression of *egl-21* in three branches corresponding to postmitotic neurons. O) An endogenous GFP reporter of *vab-7* is expressed in the PVW and PHC neuron pairs in the tail in wild-type (top) but not *lin-32(u282)* animals (bottom). *vab-7* expression is decreased in PVW and PHC in *lin-32* mutants (right). Fisher’s Exact Test, p = 2.243×10^−7^.

Supplement 5 **Supplemental Figure 5. Distinguishing P0-P1 and P2-P12 derived progenitors and neurons.** (A) Sub-UMAP of the P0-P12 lineages, colored by cell type. (B) Diagrams of the P0, P1, P3-P8 lineages, which adopt related but distinct patterns of division for a subset of cells. (C) Sub-UMAP of P0-P1 progenitors and neurons, colored by cell type. Sub-UMAP in C, colored by expression of the Hox genes (D) *ceh-13,* (E) *lin-39,* and (F) *mab-5*.(G) Sub-UMAP of P2-P12 progenitors and neurons, colored by cell type. Sub-UMAP in G (Same as Fig 3E), colored by expression of the Hox genes (H) *ceh-13*, (I) *lin-39* and (J) *mab-5*. (K) Sub-UMAP of P.ap progenitors, VD neurons, and AS neurons, showing the VD subclasses VD2, VD12, and VD13. (L) Sub-UMAP as in panel K, colored by expression of the Hox gene *egl-5*. (M) Sub-UMAP of P.aaa progenitors, VA neurons, and VB neurons, showing lack of distinct VA and VB subclasses. N) Dotplot showing the expression of 49 transcription factors previously assigned to post-embyronic neuronal progenitors across all scRNA-seq cell types.

Supplement 6 **Supplemental Figure 6. Expression of selected gene families in the larval nervous system.** A) Scatterplot showing the relationship between the aggregate number of genes detected in each neuron class (y axis) using threshold 2 and the number of cells captured per neuron class (x axis) in L1. B) Scatterplot showing true positive rate (TPR, x-axis) and number of genes detected on aggregate (y-axis) for each neuron class from threshold 2. Labeled neuron classes in red show the lowest TPRs and numbers of genes and are more likely to have false negatives. C-F) Combined boxplot and jitter plots showing the number of genes belonging to selected gene families detected per neuron class in L1 (left) and in L4 (right) for embryonic (light green) and post-embryonic (dark green) neurons. Gene families: C) ligand-gated ion channels, D) ribosomal proteins, E) GPCR neuropeptide receptors and F) metabotropic neurotransmitter receptors. All statistical tests were performed using linear models featuring the birthtime, stage, and number of cells per neuron class as covariates. Between group comparisons were performed on the estimated marginal means using the Tukey p-value adjustment for multiple comparisons. * p-value < 0.05, ** p-value < 0.01, *** p-value < 0.001, **** p-value < 0.0001.

Supplement 7 **Supplemental Figure 7.** A) Venn diagrams showing overlap of Differentially Expressed Genes (DEGs) between L1 and L4 in scRNA-Seq and data from (Sun and Hobert, 2021). There was significant overlap between scRNA-seq comparisons and bulk neuronal samples using INTACT (left), between scRNA-seq and reporter strain data for cases where expression was higher in L4 than L1 (middle left), and between scRNA-seq and reporter strain data for stable expression (middle right). There was less overlap for cases with higher expression in L1 than L4 (rightmost). One-sided Fisher’s Exact Tests were used to test for significant overlap. B-F) Validation of individual DEGs between L1 and L4. B) Dotplot denoting a subset of DEGs between the L1 and L4 in specific neurons compared to fluorescent reporter strains. The color scale reflects the log2 fold change from scRNA-seq data (red, positive values = higher in L4; blue, negative values = higher in L1). The shape of the point reflects whether the fluorescent reporter was consistent with scRNA-seq data. Squares indicate the reporter showed the same differential expression as scRNA-seq. Circles indicate the reporter did not show differential expression. Some of these genes showed differential expression in additional neuron classes which were not tested for validation. C) Left: Micrographs of an endogenous *lep-5* GFP reporter in CAN in L1 and L4. The CAN cell body was labeled with an endogenous *swt-3*::RFP marker. Right: Quantification of *lep-5* expression. D) Left: Confocal micrograph of an endogenous *ins-30* GFP reporter in CAN in L1 vs L4. The CAN cell body was labeled with a *pks-1*::TagRFP transgene. Right: Quantification of *ins-30* reporter expression between L1 and L4. Fisher’s Exact Test. E) Left: Micrographs of *srlf-1* expression in HSN (labeled by *cat-1* reporter fosmid in L1 and L4. Right: Quantification of decreased *srlf-1* expression in HSN from L1 to L4. Fisher’s Exact Test. F) Left: Micrographs of an endogenous *flp-27::*GFP reporter in L1 (top) and L4 (bottom). Right: Quantification of *flp-27*::GFP intensity in ALM, BDU and PLM. ALM and PLM showed significantly decreased *flp-27* reporter expression from L1 to L4, whereas BDU showed no change. Unpaired t-tests. * = p-value < 0.05, ** p-value ≤ 0.01, *** p-value ≤ 0.001, **** p-value ≤ 0.0001.

Supplement 8 **Supplemental Figure 8.** A) Dotplot showing validation results for five endogenous neuropeptide reporters tested for differential expression between L1 and L4 in embryonically derived neurons (x- axis). All neurons with differential expression from scRNA-seq for these genes are shown. Color scale represents log2 fold change values from scRNA-seq data. Positive values (orange - red) = higher in L4, negative values (blue) = higher in L1. Shape represents the result of fluorescent reporter validation: squares indicate the reporter expression was consistent with scRNA-seq. Circles indicate the reporter was expressed in the given neuron but did not show differential expression between L1 and L4. Diamonds indicate the reporter was not detected in the neuron in either L1 or L4. B-F) Micrographs and quantification of reporter validation. Neurons with stable expression are not labeled in the micrographs for clarity. Postembyronically-derived neurons were not scored for validation due to lack of NeuroPAL coloring at L1. B) Left: Micrographs of *flp-32* reporter expression in the head at L1 (top) and L4 (bottom). Right: quantification of GFP intensity in seven neuron classes. C) Left: Micrographs of *ins-5* reporter expression in the head at L1 (top) and L4 (bottom). Right: quantification of GFP. The reporter showed a significant increase in AFD, the change in scRNA-seq data in AFD was not significant. D) Left: Micrographs of *flp-5* reporter expression in the head, midbody and tail at L1 (top) and L4 (bottom). Right: quantification of GFP intensity. E) Left: Micrographs of *nlp-73* reporter expression in the midbody and tail at L1 (top) and L4 (bottom). Right: quantification of GFP intensity. F) Left: Micrographs of *nlp-64* reporter in L1 (top) and L4 (bottom). Right: quantification of GFP intensity in AVE and CAN. Mann-Whitney U tests were used for significance testing. * = p-value < 0.05, ** p-value ≤ 0.01, *** p-value ≤ 0.001, **** p-value ≤ 0.0001.

Supplement 9 **Supplemental Figure 9. Ciliated neurons share differential expression between the embryo and L1.** A) Heatmap as in Figure 5A showing genes (columns) differentially expressed across the nervous system (neurons on y-axis). Neuron categories are depicted by the color scale on the left. Gene sets used for gene set enrichment analysis are depicted by the color bars on the bottom and labeled on the right. B) Wormcat enrichment analysis for five gene sets that showed clustered differential expression (labeled on right, corresponding to labels in panel A). Wormcat gene annotations are shown on the x-axis. The color scale represents fold enrichment over background, and categories with red borders were significant. Fisher’s Exact Test with Bonferroni-corrected p-values. Ciliated neurons shared patterns of higher expression in the embryo of genes (dark blue bars) related to cilia formation. C) Bar graph showing the number of DEGs (Differentially Expressed Genes) per neuron class in the embryo vs L1 comparison, colored by functional category. The horizontal dashed line marks the median of 571 DEGs/neuron. D) Box and jitterplot showing the number of DEGs per neuron class in the embryo vs L1 comparison grouped by functional category.

Supplement 10 **Supplemental Figure 10.** A) Heatmap as in Figure 5B showing differential gene expression between L1 and L4. Columns are genes, rows are neurons. Color scales on y-axis (left) denote functional groups and birthtimes. Hierarchical clustering grouped genes with similar patterns together. Color bars across columns indicate gene clusters. Colored bars below heatmap indicate gene sets that were used as input to Wormcat for gene set enrichment analysis, based on shared pattens of differential expression in multiple related neuron classes, as indicated by labels on the right. B) Gene set enrichment analysis for all DE genes (green, top row), or subsets of genes with shared differential expression across groups of neurons. Wormcat annotation categories are shown on the x-axis. Color scale reflects fold enrichment of genes in each category in the queried gene set over background (all genes detected by scRNA-seq). Dots with red borders were significantly enriched (Bonferroni-corrected p-value < 0.05). Significance was tested with Fisher’s Exact Test followed by Bonferroni correction for multiple comparisons. C) Bar graph showing the number of DEGs/neuron class for the L1 vs L4 comparison, with neurons colored by functional category. The median of 227 DEGs/neuron is depicted by the horizontal dashed line. D) Box and jitterplot of the number of DEGs/neuron grouped by functional category. E) Bar graph of DEGs/neuron for L1 vs L4 as in C but colored by birthtime (embryonic vs postembryonic). F) Box and jitterplot showing the number of DEGs/neuron grouped by birthtime. Postembryonically-derived neurons exhibited more DEGs/neuron than embryonically derived neurons. Linear regression with pairwise comparisons of estimated marginal means and Tukey’s method for multiple comparisons.

Supplement 11 **Supplemental Figure 11.** A) Heatmap displaying the log2 fold change between L1 and L4 for ribosomal protein encoding genes (columns) with significant differential expression. Positive log2 fold change values (orange, red) reflect higher expression in L4, negative values (blue) reflect higher expression in the L1 stage. Color scales on y-axis (left) denote functional groups and birthtimes. B) Confocal micrographs showing RPS-4::GFP expression in hypodermis, muscle, intestine and neurons in the anterior regions of L1 (left) and L4 (center) larvae. Quantification (right) of fluorescence intensity shows a reduction in intensity among neurons (p = 0.0504) and intestine between L1 and L4 stages. C) Confocal micrographs of RPL-7A::GFP expression in anterior regions of L1 (left) and L4 (center). Quantification (right) of fluorescence intensity shows decreased expression in neurons, hypodermis and intestine, with no change in muscle, from L1 to L4. D) Confocal micrographs showing RPL-25.1::GFP expression in hypodermis, muscle, intestine and neurons in the anterior regions of L1 (left) and L4 (center) larvae. Quantification (right) of fluorescence intensity shows significant increases in RPL-25.1 levels in neurons, muscle and hypodermis, but not intestine, between the L1 and L4 stages. 2-way ANOVA with Sidak’s correction.

Supplement 12 **Supplemental Figure 12. Morphological description of neurons at the end of the first larval stage in *C. elegans*.** A-B) Morphological descriptions of neurons born during the first developmental stage (L1) are interpreted from annotated EM images of *C. elegans* 16h after hatching^6^. Shown are EM images of the last annotation for each neuron, representing the end of the neuron at this developmental stage. Neuron morphologies are compared to the fully matured adult stage descriptions published in the Mind of the Worm^26^ (MoW). Panel A features neurons that are fully matured at the end of the L1 larval stage. Panel B features neurons whose morphology is not fully matured at this stage. C) Table adapted from^20^. Neurons born and removed during each developmental stage, transdifferentiation events and differentiation/maturation of hermaphrodite neurons are listed.

Supplement 13 **Supplemental Figure 13. Thresholded neuropeptidergic networks (mid-range) of the 88 NPP-GPCR pairs conserved between L1 and L4 larval stages.** The adjacency matrices display the developmental pattern of connections between sending neurons (y-axis) and receiving neurons (x-axis). Columns and rows are sorted by neuron class as in Figure 6 panel A. Core (conserved) connections and developmentally-dynamic connections are color-coded.

Supplement 14 **Supplemental Figure 14.** (A) Degree distributions of *C. elegans* L1 neural networks. In each case, degree (incoming plus outgoing connections) is shown in green, in-degree (incoming connections) in blue and out-degree (outgoing connections) in yellow. The 10 highest-degree hubs in each network are indicated. (B) Degree distributions of *C. elegans* L4 neural networks, displayed in the same way as panel A. (C) Top 20 neurons that show convergence (similar number of connections, Knorm) or divergence (different number of connections, Kstd) between L1 and L4 larval stages.

Supplement 15 **Supplemental Figure 15.** A) Volcano plot showing enrichment of CAMs based on synaptic connectivity. Each dot represents a comparison of one gene tested in the synaptically-connected vs adjacent only neurons for a given neuron. Negative log fold change values represent enrichment in non-synaptic adjacent cells, whereas positive log fold change represent enrichment in synaptically-connected cells. Light gray dots represent instances with Benjamini-Hochberg adjusted p-values > 0.05. Dark gray dots represent cases with adjusted p-values < 0.05. Black dots represent cases with adjusted p-values < 0.05 and in which the tested gene has a known binding partner expressed in the neuron of interest. B) Top: Schematic representation of the AIY neurons, from WormAtlas. Bottom: Adjacent neurons with only membrane contact (left) or synaptic inputs (right) with AIY. Only a subset of adjacent neurons with membrane contact are shown. C) 3D reconstruction from NeuroScan of late L1 nerve ring featuring AIYR, AUAR and AFDR. Both AUAR (light blue) and AFDR (orange) contact AIYR (dark blue and red, respectively), but only AFDR is presynaptic to AIYR (red triangle). D) Heatmap showing the log fold change of 15 CAMs enriched in AIY + non-synaptic adjacent cells (below red line) compared to AIY + presynaptic inputs (above red line). Genes are sorted by log fold change (lowest fold change on left). Genes with known binding partners expressed in AIY are denoted with red arrows, and the respective binding partners are listed below. E) Heatmap showing several genes with large fold changes between AIA synaptically connected neurons and membrane adjacent-only neurons. These eight genes are expressed in only a subset of AIA presynaptic inputs and therefore have adjusted p-values > 0.05. They may, however, still regulate specific individual connections. F) Scatterplot showing -log10 transformed p-values for each gene-neuron combination in L1 (x-axis) and in L4 (y-axis). Genes only detected at one age are not shown. Significant cases (Benjamini-Hochberg adjusted p-value <0.05) of enrichment in L1 (pink), cases significantly enriched at L4 (blue), and cases enriched at both L1 and L4 (green). The p-value adjustments were made for multiple genes within each neuron.

Supplement 16 Supplemental Table 1. List of strains used in this study.
Supplemental Table 2. 10X Genomics sample information.
Supplemental Table 3. Table of the number of cells captured for each cell type in each experimental sample.
Supplemental Table 4. Genes used to annotate progenitor cell classes in this study.
Supplemental Table 5. Genes with significant correlations to maturation index in SDQ, AVM and PVM.
Supplemental Table 6. Comparison of sequencing statistics for ventral cord motor neurons in this study and in previous studies^16,28^.
Supplemental Table 8. Genes excluded from differential expression results due to overexpression in transgenic constructs.
Supplemental Table 9. Sample metadata from re-analysis of embryonic single cell data from Packer, et al., 2019^24^.
Supplemental Table 12. Results from gene regulatory network analysis using *Cel*EsT on differentially expressed genes between embryo and L1 stages.
Supplemental Table 13. Results from gene regulatory network analysis using *Cel*EsT on differentially expressed genes between L1 and L4 stages.

Supplement 17 Supplemental Table 7. Differential gene expression results between cell types in the P lineage.

Supplement 18 Supplemental Table 18. Enrichment test results for cell surface and secreted molecules in postsynaptic outputs vs adjacent, non-synaptic neurons for each nerve ring neuron in L1 and L4.

Supplement 19 Supplemental Table 19. Enrichment test results for cell surface and secreted molecules in presynaptic inputs vs adjacent, non-synaptic neurons for each nerve ring neuron in L1 and L4.

Supplement 20 Supplemental Table 14. L1 ground truth matrix used for thresholding accuracy.

Supplement 21 Supplemental Table 15. Thresholding metrics (TPR, FPR, FDR) from comparing four thresholded datasets to ground truth for each neuron class.

Supplement 22 Supplemental Table 16. Changes in neuropeptide network modules between the L1 and L4 stages.

Supplemental Table 10. Differential gene expression results for 89 neurons between the embryonic and L1 stages.

Supplemental Table 11. Differential gene expression results for 121 neurons between the L1 and L4 stages.

Supplemental Table 17. Enrichment test results for cell surface and secreted molecules based on membrane contact.
