# Supplementary figures and images for: A gene expression atlas of a juvenile nervous system
Source: bioRxiv. 2025 Nov 22:2025.11.21.689793. Preprint. [Version 1] doi: 10.1101/2025.11.21.689793 (PMC12667811; doi:10.1101/2025.11.21.689793)

### Estimated age range of animals

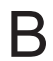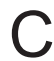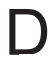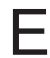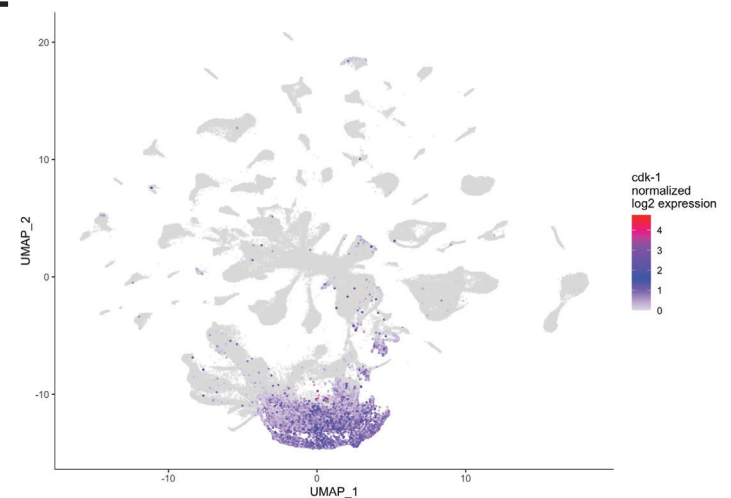

Supplement: Supplement 1 — Supplemental Figure 1. Single-cell RNA sequencing of L1 larvae. A) Graphical representation of neuron-specific fluorescent reporter strains and developmental ages sampled for scRNA-Seq. The rab-3, flp-7 and ceh-34; unc-4 reporter strains were used for two experimental samples each. B) Schematic of mesh protocol used to generate large synchronous cultures (top) and use of FACS to enrich for targeted cell populations (bottom). C) UMAP showing the entire dataset of 161,562 cells, colored by cell type annotation. D) UMAP of all cells colored by expression of the pan-neural gene sbt-1 marking post-mitotic neurons. E) UMAP of all cells colored by expression of the cell cycle gene cdk-1 marking progenitor cells. [file media-1.pdf]

**A**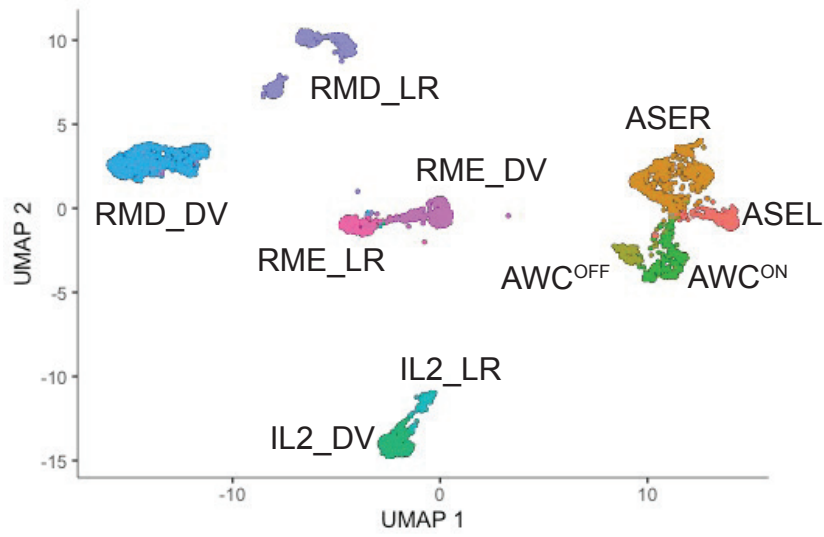**B**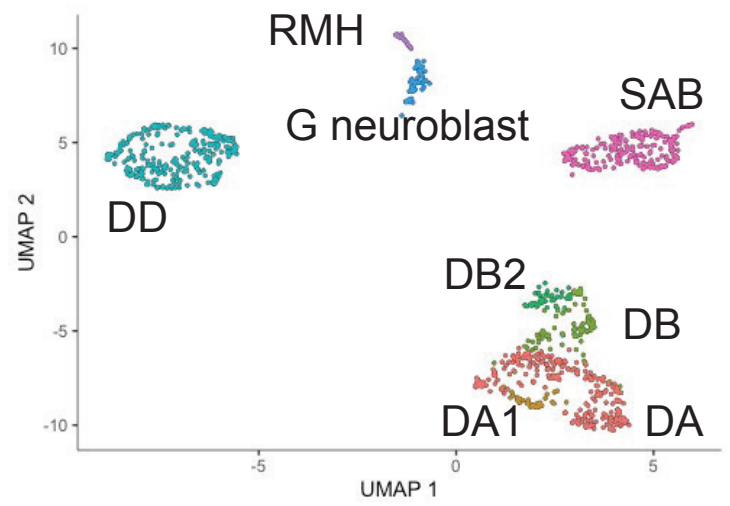**C**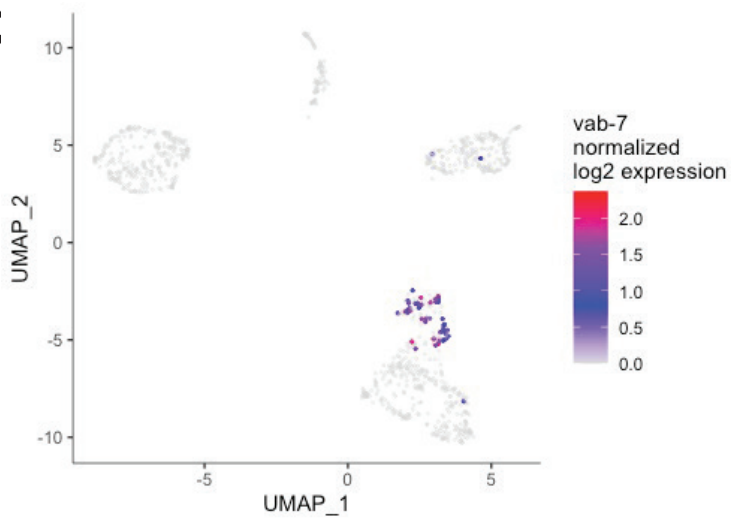**D**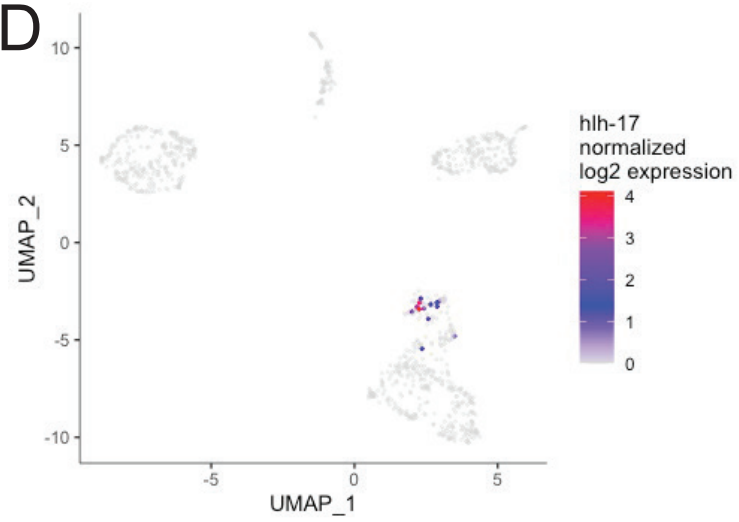**E**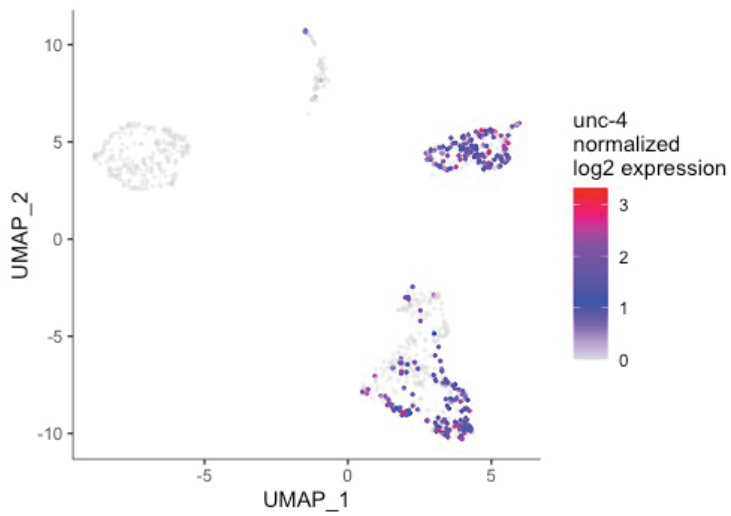**F**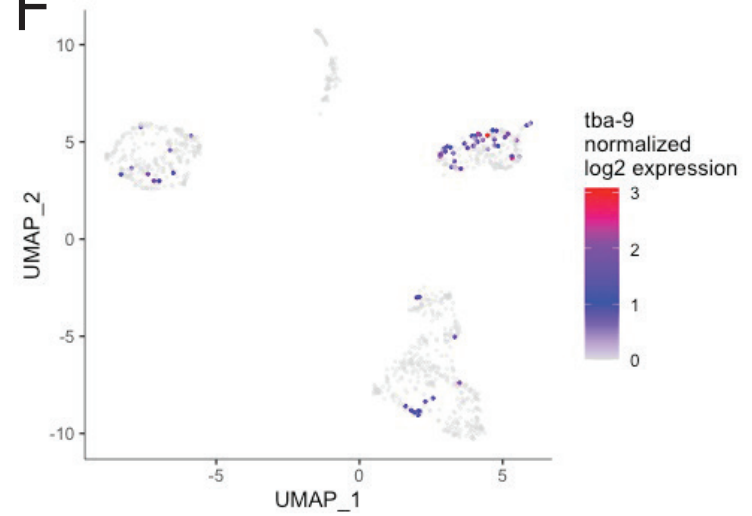

Supplement: Supplement 2 — Supplemental Figure 2. Neuronal subclasses are present in the L1. A) Sub-UMAP showing separation of neuronal subclasses for RMD, RME, IL2, ASE, and AWC. B) Sub-UMAP showing embryonic motor neurons that include the newborn RMH cluster, the G neuroblast, DD, SAB, and subclasses of the DA and DB motor neurons. C) Sub-UMAP showing expression of the DB class marker vab-7. D) Sub-UMAP showing expression of the DB2-specific marker hlh-17. E) Expression of the DA and SAB class marker unc-4. F) Expression of the tubulin tba-9, which is restricted to DA1 among DA neurons28. [file media-2.pdf]

A

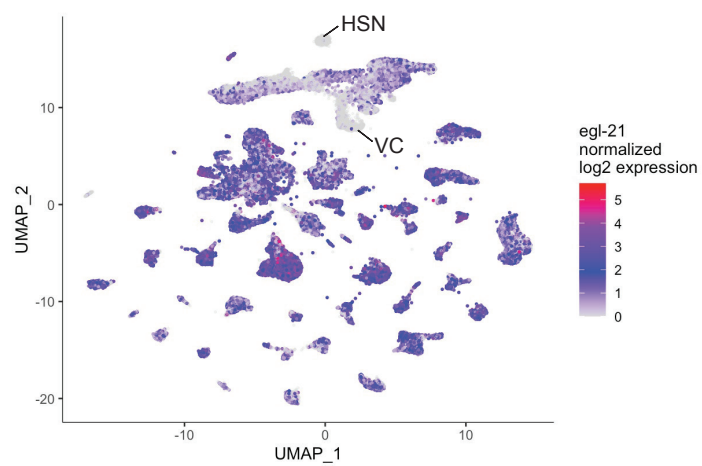

B

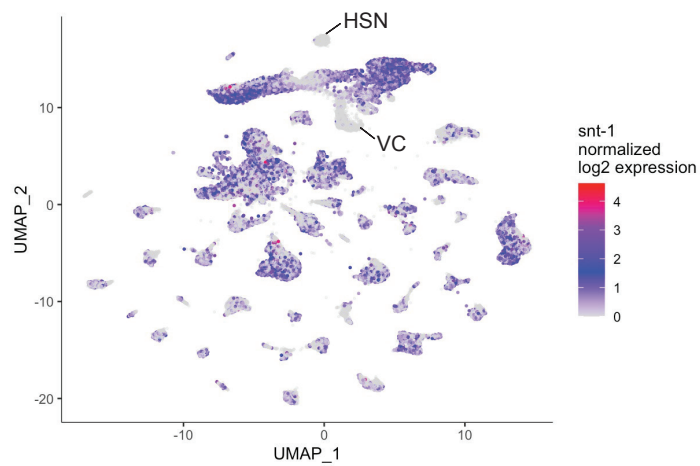

C

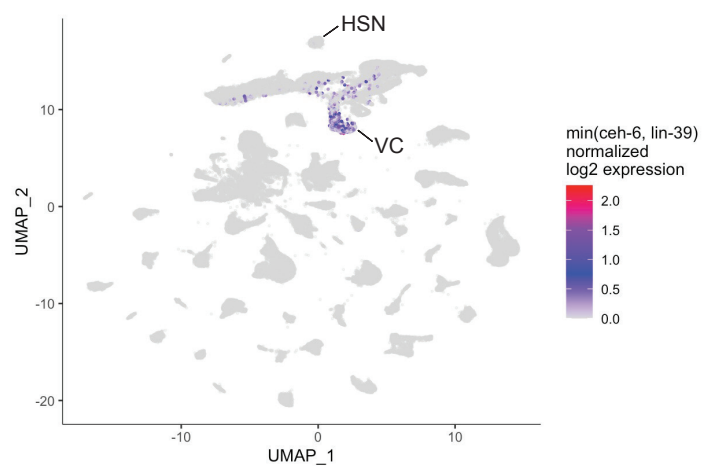

D

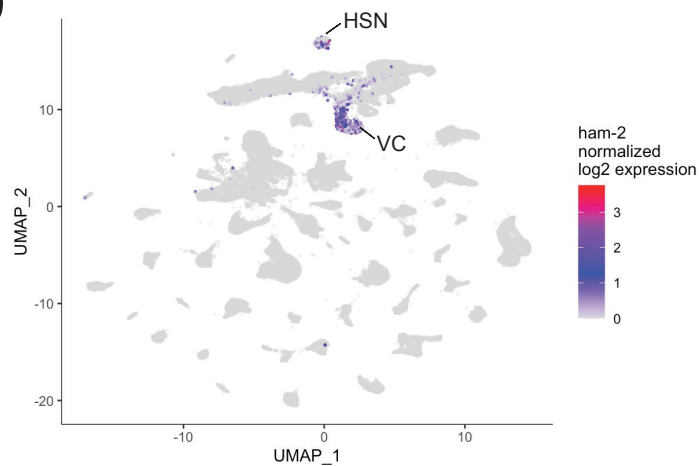

E

*wgl-748 ceh-6::TY1::EGFP*

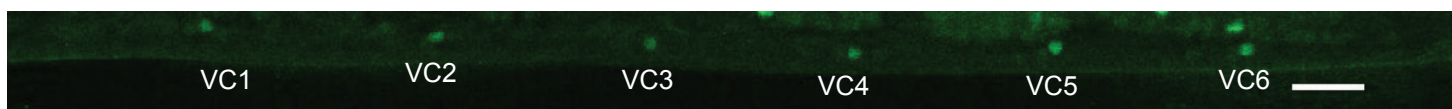

Supplement: Supplement 3 — Supplemental Figure 3. Annotation of VC neurons in L1. L1 HSN and VC neurons express low levels of the pan-neural genes egl-21 (A) and snt-1 (B). C) The VC neuron cluster uniquely shows co-expression of the transcription factors ceh-6 and lin-39. D) UMAP of ham-2 expression, which is restricted to HSN and VC. [file media-3.pdf]

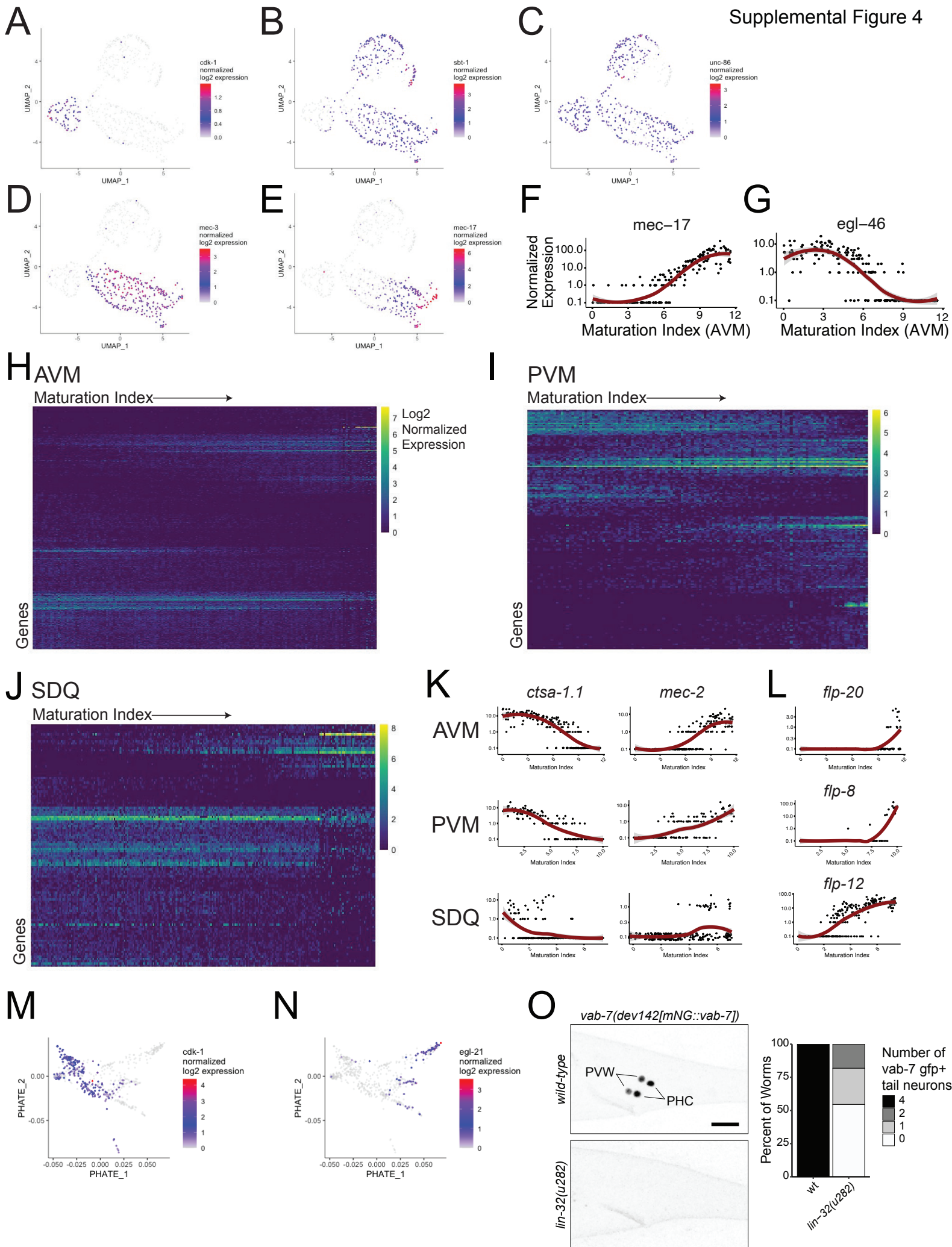

Supplement: Supplement 4 — Supplemental Figure 4. Q and T lineage progenitors and neurons in L1. A-E) Sub-UMAPs of the Q.pa lineage showing expression of A) the cell cycle gene cdk-1 marking the Q.pa cluster B) sbt-1 in post-mitotic neurons, C) unc-86 in Q.pa, AVM and PVM, but with decreased expression in the SDQ cluster (top right), especially in cells further from the Q.pa cluster. D) Expression of mec-3 is stable among all AVM and PVM neurons. E) Expression of mec-17 increases in both AVM and PVM in cells with increasing UMAP distance from the Q.pa progenitor cluster. F) Jitter plot of mec-17 expression (y-axis) by maturation index (x-axis) for AVM. Maroon line shows the smoothed mean expression using the loess method. G) Jitter plot as in F, but for egl-46. Note the decrease with maturation index. H) Heatmap of log2 expression of 530 genes that showed significant statistical correlations (Bonferroni-corrected p-value < 0.05) with the maturation index in AVM. The columns are cells ordered by increasing maturation index. Rows are genes, clustered by similarity. I) Heatmap as in H, but for 475 genes with statistically significant correlation with maturation index in PVM. J) Heatmap as in H, I, but for 395 genes with significant correlation with maturation index in SDQ. K) Jitter plots showing shared regulation of genes across early postmitotic development in AVM, PVM, and SDQ. The lysosomal enzyme cathepsin A, ctsa-1.1 (left), shows shared downregulation with maturation in all three neurons, whereas mec-2 showed shared upregulation with maturation (right). L) The neuropeptide encoding genes flp-20, flp-8 and flp-12 showed cell-type specific upregulation. M) PHATE plot of the T-lineage showing expression of cdk-1, which marks neuronal progenitors. N) PHATE plot of the T-lineage showing expression of egl-21 in three branches corresponding to postmitotic neurons. O) An endogenous GFP reporter of vab-7 is expressed in the PVW and PHC neuron pairs in the tail in wild-type (top) but not lin-32(u28 [file media-4.pdf]

Supplemental Figure 5

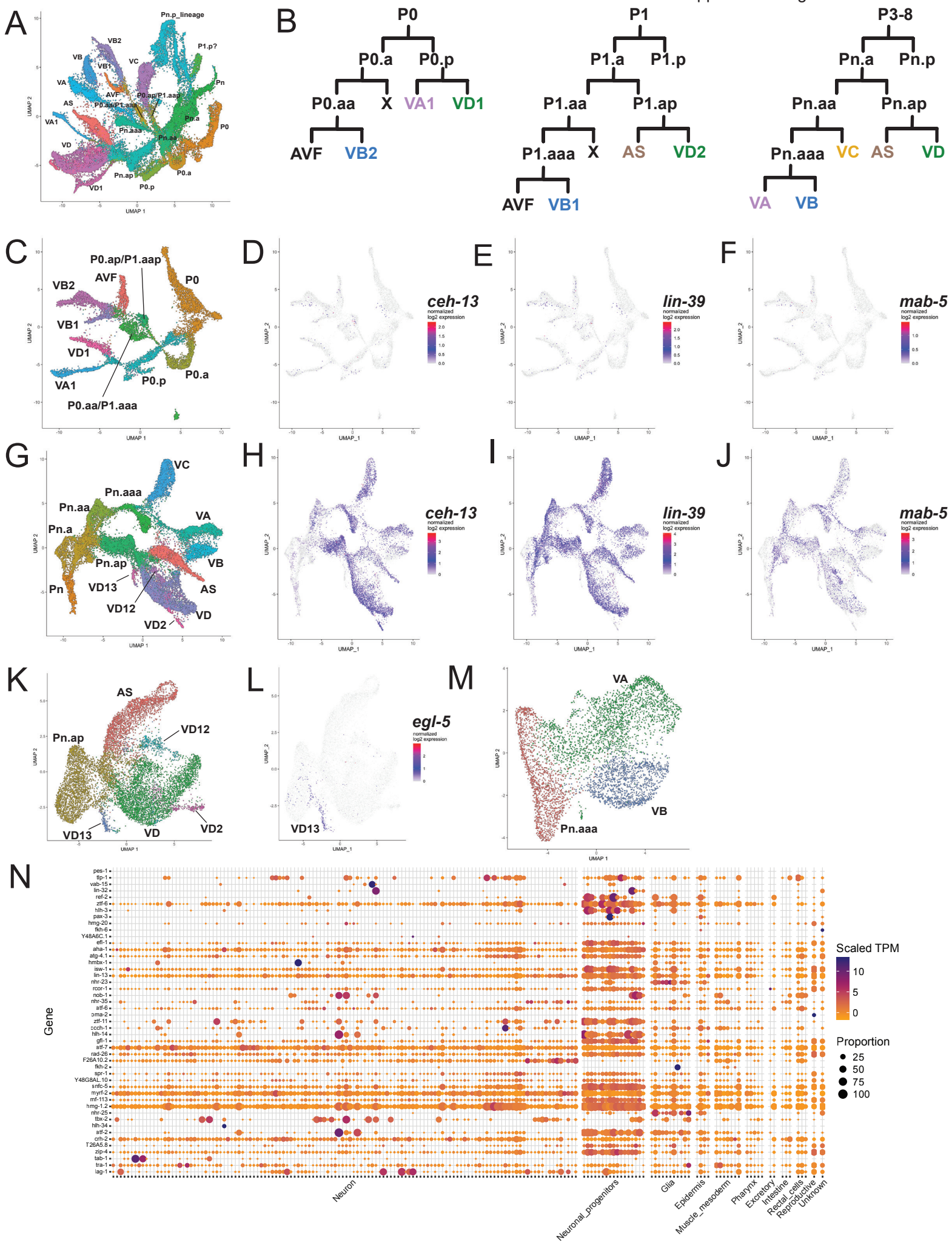

Supplement: Supplement 5 — Supplemental Figure 5. Distinguishing P0-P1 and P2-P12 derived progenitors and neurons. (A) Sub-UMAP of the P0-P12 lineages, colored by cell type. (B) Diagrams of the P0, P1, P3-P8 lineages, which adopt related but distinct patterns of division for a subset of cells. (C) Sub-UMAP of P0-P1 progenitors and neurons, colored by cell type. Sub-UMAP in C, colored by expression of the Hox genes (D) ceh-13, (E) lin-39, and (F) mab-5.(G) Sub-UMAP of P2-P12 progenitors and neurons, colored by cell type. Sub-UMAP in G (Same as Fig 3E), colored by expression of the Hox genes (H) ceh-13, (I) lin-39 and (J) mab-5. (K) Sub-UMAP of P.ap progenitors, VD neurons, and AS neurons, showing the VD subclasses VD2, VD12, and VD13. (L) Sub-UMAP as in panel K, colored by expression of the Hox gene egl-5. (M) Sub-UMAP of P.aaa progenitors, VA neurons, and VB neurons, showing lack of distinct VA and VB subclasses. N) Dotplot showing the expression of 49 transcription factors previously assigned to post-embyronic neuronal progenitors across all scRNA-seq cell types. [file media-5.pdf]

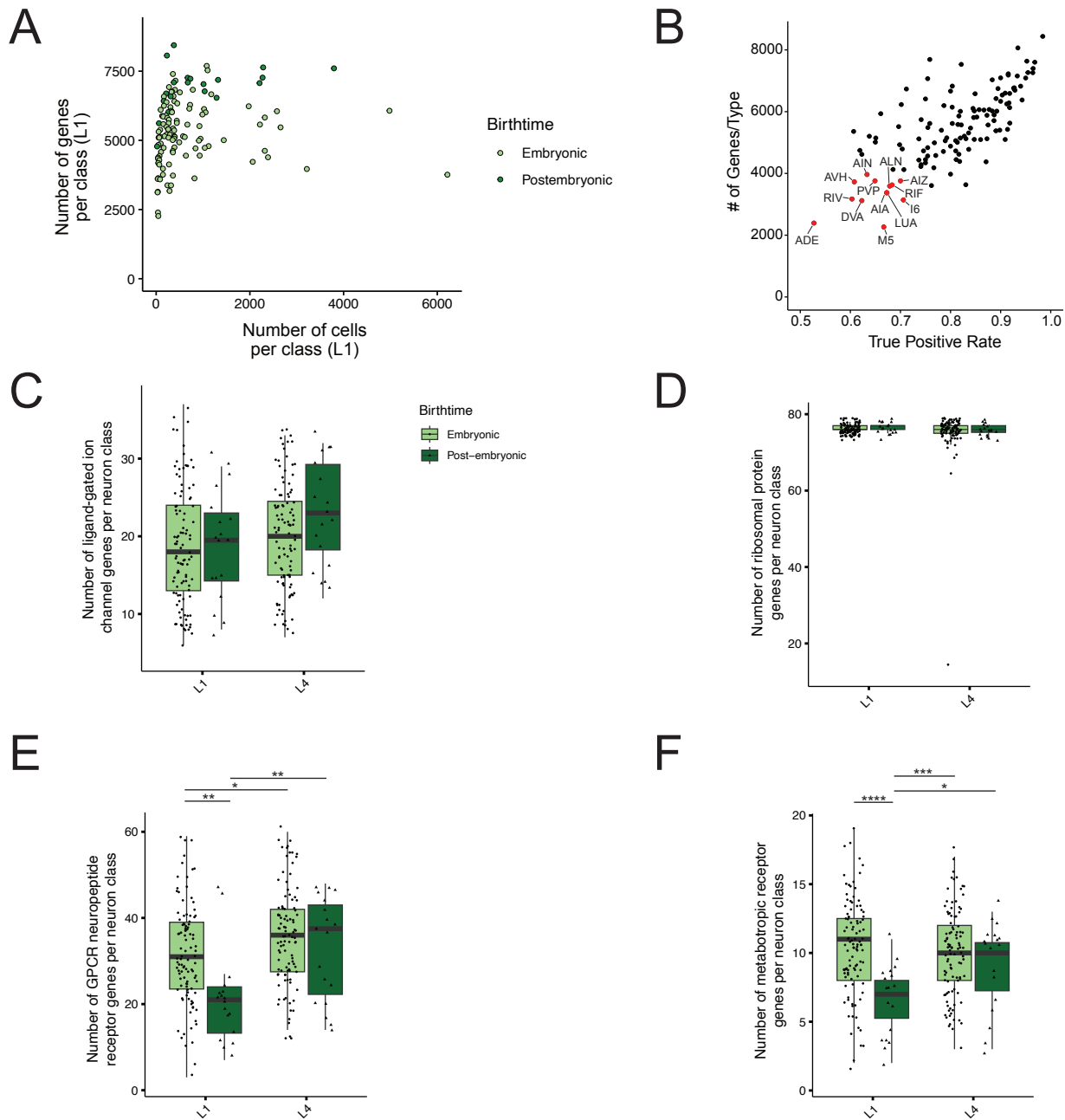

Supplement: Supplement 6 — Supplemental Figure 6. Expression of selected gene families in the larval nervous system. A) Scatterplot showing the relationship between the aggregate number of genes detected in each neuron class (y axis) using threshold 2 and the number of cells captured per neuron class (x axis) in L1. B) Scatterplot showing true positive rate (TPR, x-axis) and number of genes detected on aggregate (y-axis) for each neuron class from threshold 2. Labeled neuron classes in red show the lowest TPRs and numbers of genes and are more likely to have false negatives. C-F) Combined boxplot and jitter plots showing the number of genes belonging to selected gene families detected per neuron class in L1 (left) and in L4 (right) for embryonic (light green) and post-embryonic (dark green) neurons. Gene families: C) ligand-gated ion channels, D) ribosomal proteins, E) GPCR neuropeptide receptors and F) metabotropic neurotransmitter receptors. All statistical tests were performed using linear models featuring the birthtime, stage, and number of cells per neuron class as covariates. Between group comparisons were performed on the estimated marginal means using the Tukey p-value adjustment for multiple comparisons. * p-value < 0.05, ** p-value < 0.01, *** p-value < 0.001, **** p-value < 0.0001. [file media-6.pdf]

**A**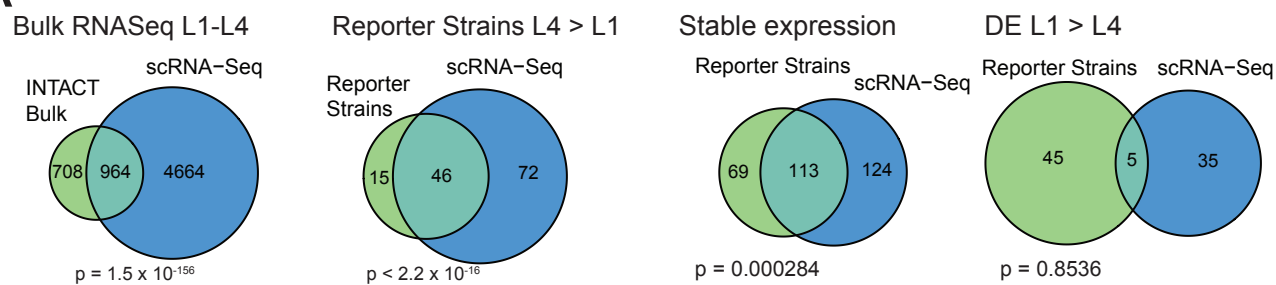**B**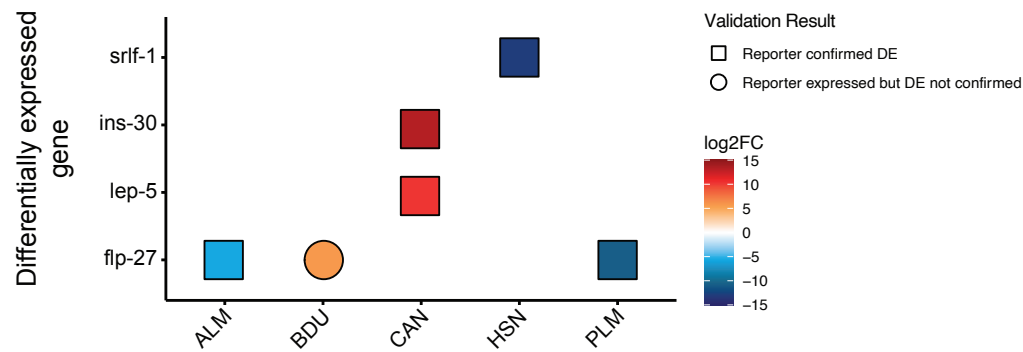**C**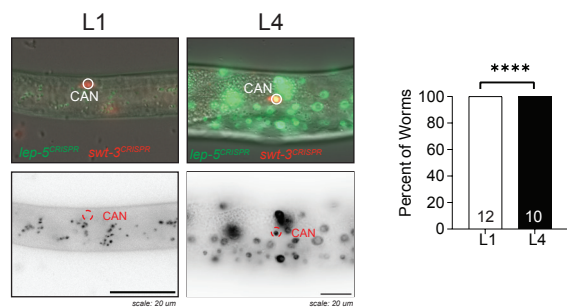**D**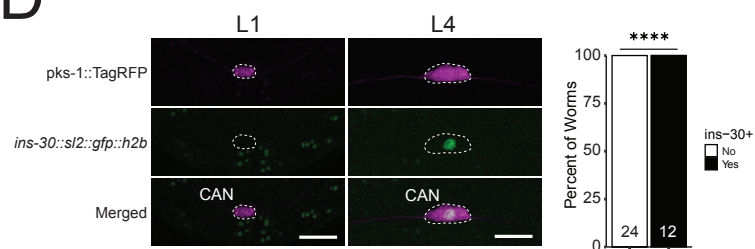**E**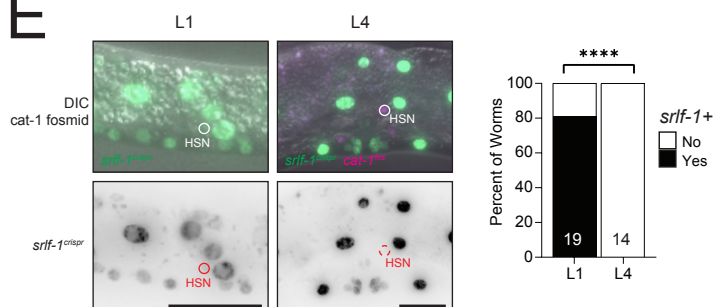**F**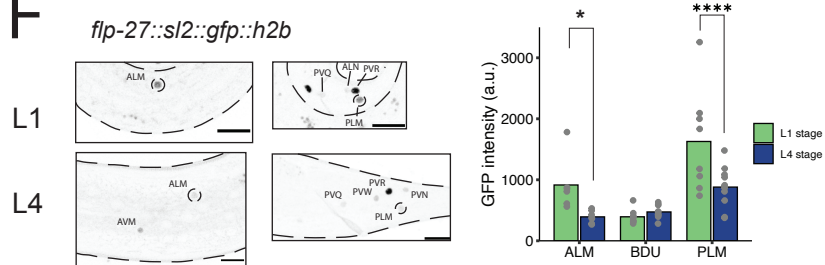

Supplement: Supplement 7 — Supplemental Figure 7. A) Venn diagrams showing overlap of Differentially Expressed Genes (DEGs) between L1 and L4 in scRNA-Seq and data from (Sun and Hobert, 2021). There was significant overlap between scRNA-seq comparisons and bulk neuronal samples using INTACT (left), between scRNA-seq and reporter strain data for cases where expression was higher in L4 than L1 (middle left), and between scRNA-seq and reporter strain data for stable expression (middle right). There was less overlap for cases with higher expression in L1 than L4 (rightmost). One-sided Fisher’s Exact Tests were used to test for significant overlap. B-F) Validation of individual DEGs between L1 and L4. B) Dotplot denoting a subset of DEGs between the L1 and L4 in specific neurons compared to fluorescent reporter strains. The color scale reflects the log2 fold change from scRNA-seq data (red, positive values = higher in L4; blue, negative values = higher in L1). The shape of the point reflects whether the fluorescent reporter was consistent with scRNA-seq data. Squares indicate the reporter showed the same differential expression as scRNA-seq. Circles indicate the reporter did not show differential expression. Some of these genes showed differential expression in additional neuron classes which were not tested for validation. C) Left: Micrographs of an endogenous lep-5 GFP reporter in CAN in L1 and L4. The CAN cell body was labeled with an endogenous swt-3::RFP marker. Right: Quantification of lep-5 expression. D) Left: Confocal micrograph of an endogenous ins-30 GFP reporter in CAN in L1 vs L4. The CAN cell body was labeled with a pks-1::TagRFP transgene. Right: Quantification of ins-30 reporter expression between L1 and L4. Fisher’s Exact Test. E) Left: Micrographs of srlf-1 expression in HSN (labeled by cat-1 reporter fosmid in L1 and L4. Right: Quantification of decreased srlf-1 expression in HSN from L1 to L4. Fisher’s Exact Test. F) Left: Micrographs of an endogenous flp-27::GFP reporter in L1 [file media-7.pdf]

**A**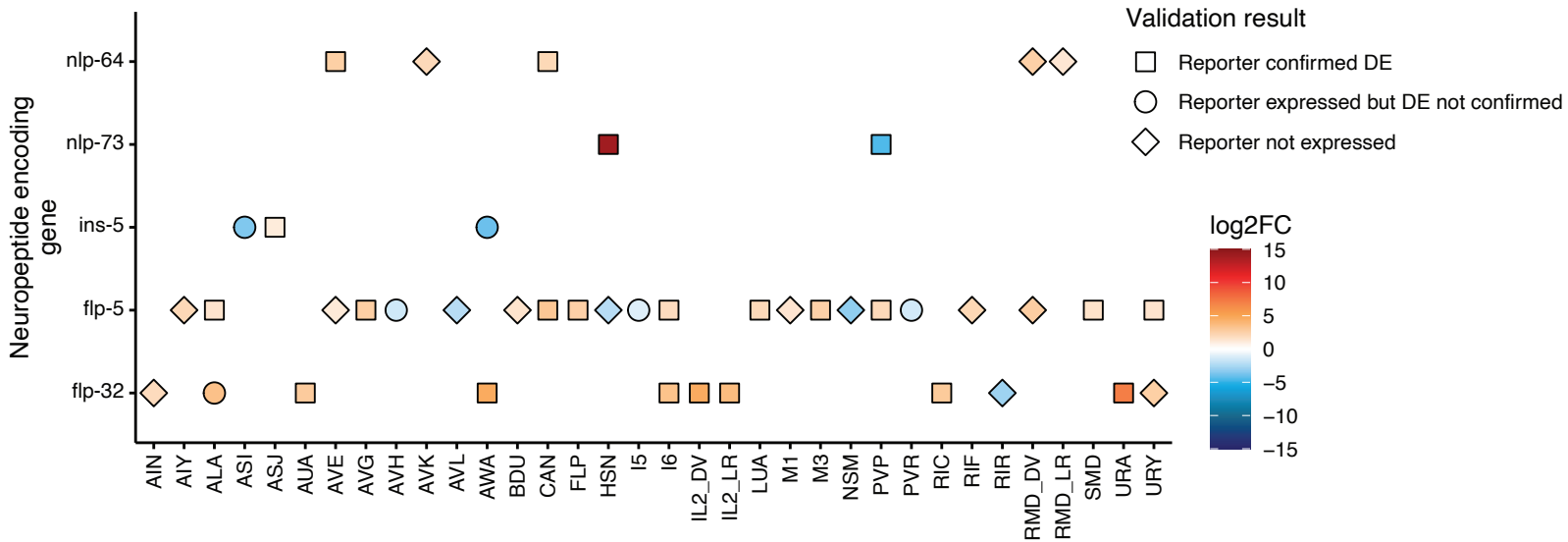**B**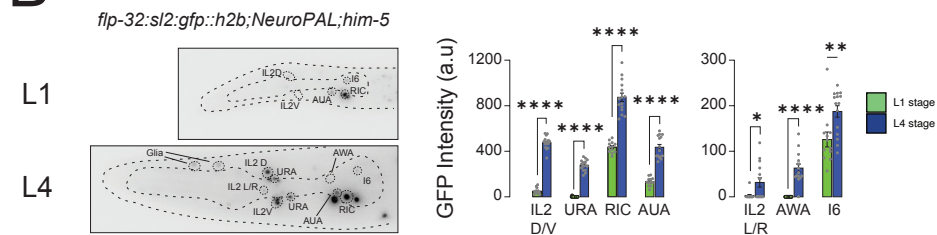**C**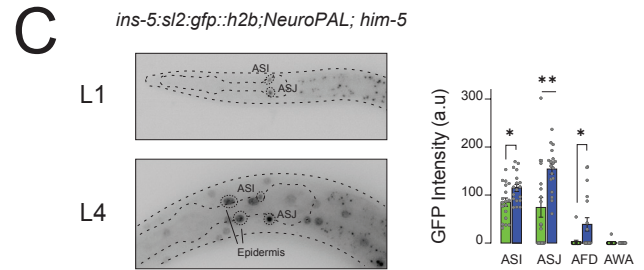**D**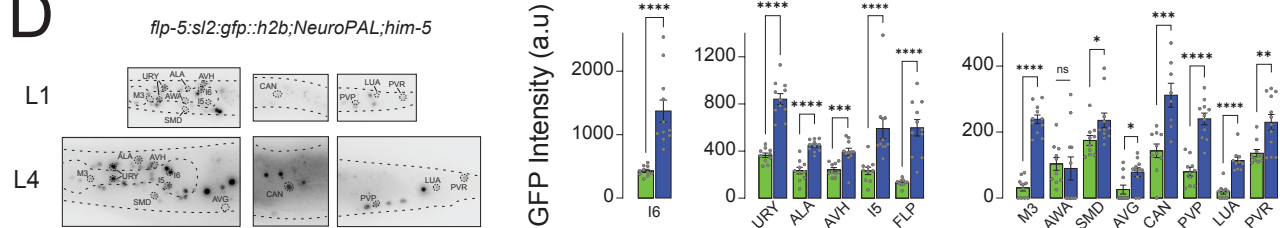**E**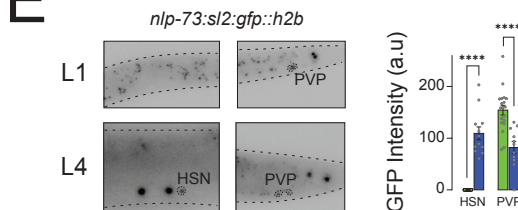**F**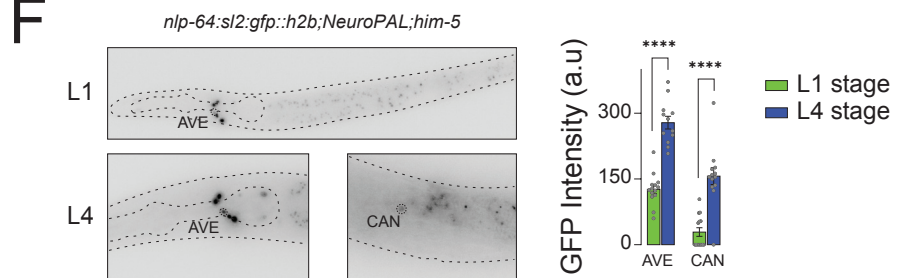

Supplement: Supplement 8 — Supplemental Figure 8. A) Dotplot showing validation results for five endogenous neuropeptide reporters tested for differential expression between L1 and L4 in embryonically derived neurons (x- axis). All neurons with differential expression from scRNA-seq for these genes are shown. Color scale represents log2 fold change values from scRNA-seq data. Positive values (orange - red) = higher in L4, negative values (blue) = higher in L1. Shape represents the result of fluorescent reporter validation: squares indicate the reporter expression was consistent with scRNA-seq. Circles indicate the reporter was expressed in the given neuron but did not show differential expression between L1 and L4. Diamonds indicate the reporter was not detected in the neuron in either L1 or L4. B-F) Micrographs and quantification of reporter validation. Neurons with stable expression are not labeled in the micrographs for clarity. Postembyronically-derived neurons were not scored for validation due to lack of NeuroPAL coloring at L1. B) Left: Micrographs of flp-32 reporter expression in the head at L1 (top) and L4 (bottom). Right: quantification of GFP intensity in seven neuron classes. C) Left: Micrographs of ins-5 reporter expression in the head at L1 (top) and L4 (bottom). Right: quantification of GFP. The reporter showed a significant increase in AFD, the change in scRNA-seq data in AFD was not significant. D) Left: Micrographs of flp-5 reporter expression in the head, midbody and tail at L1 (top) and L4 (bottom). Right: quantification of GFP intensity. E) Left: Micrographs of nlp-73 reporter expression in the midbody and tail at L1 (top) and L4 (bottom). Right: quantification of GFP intensity. F) Left: Micrographs of nlp-64 reporter in L1 (top) and L4 (bottom). Right: quantification of GFP intensity in AVE and CAN. Mann-Whitney U tests were used for significance testing. * = p-value < 0.05, ** p-value ≤ 0.01, *** p-value ≤ 0.001, **** p-value ≤ 0.0001. [file media-8.pdf]

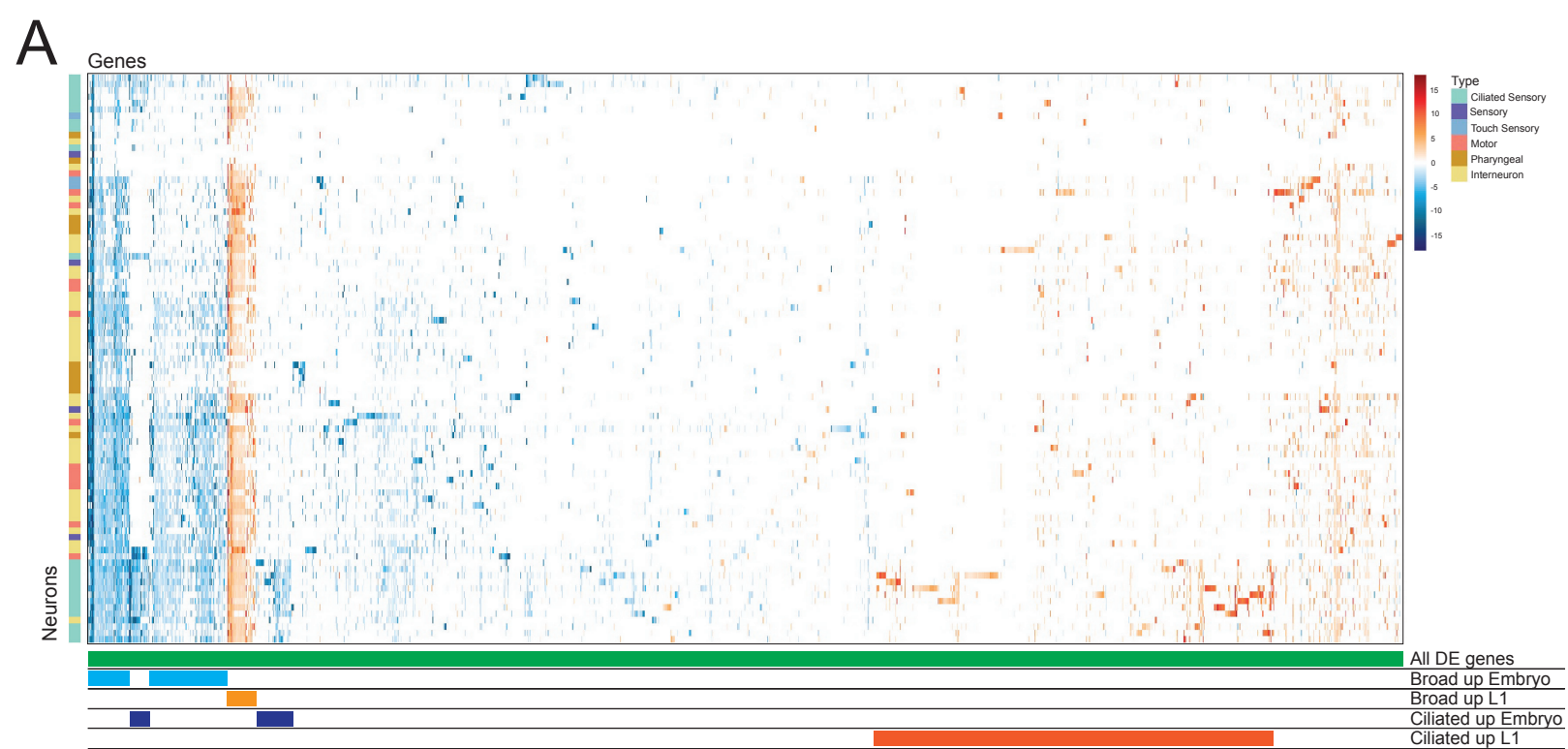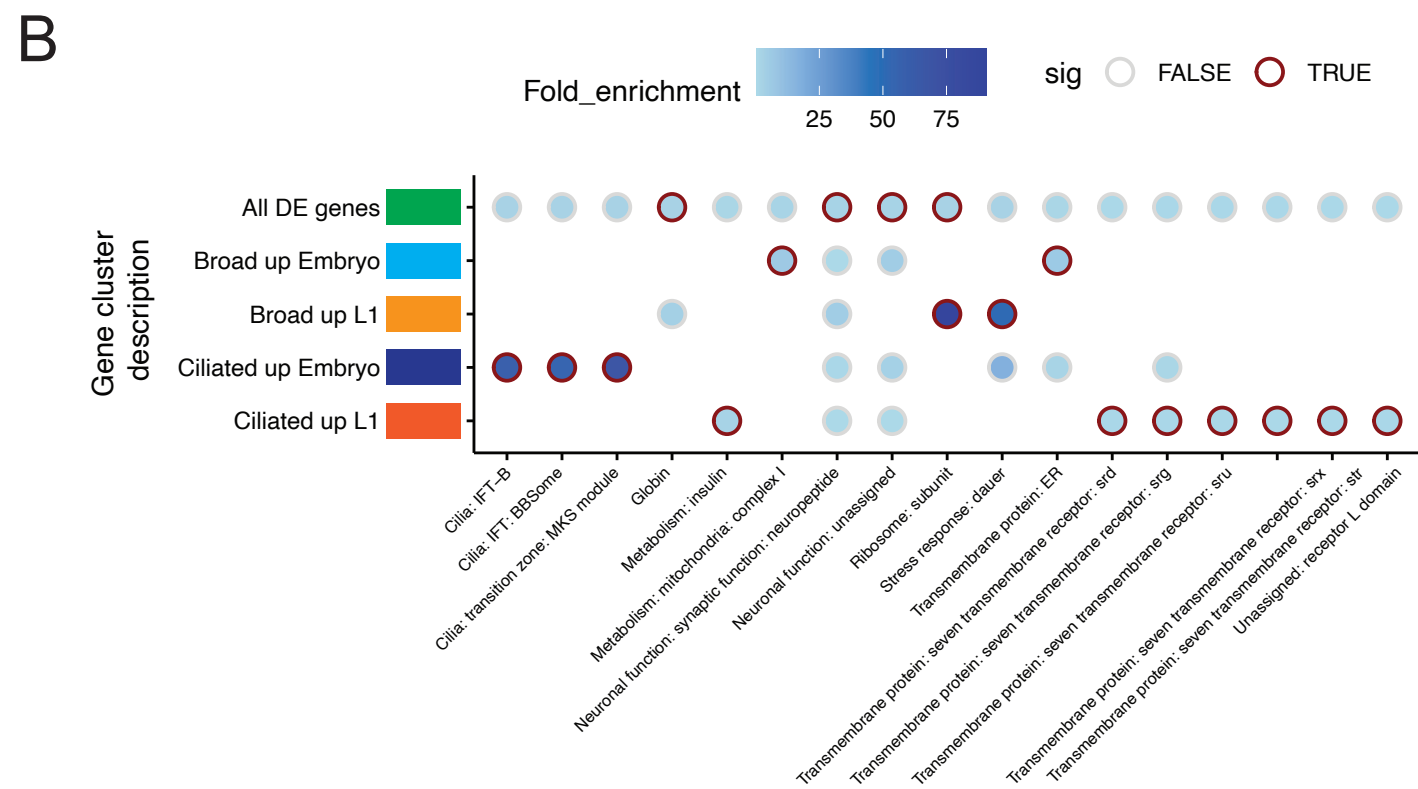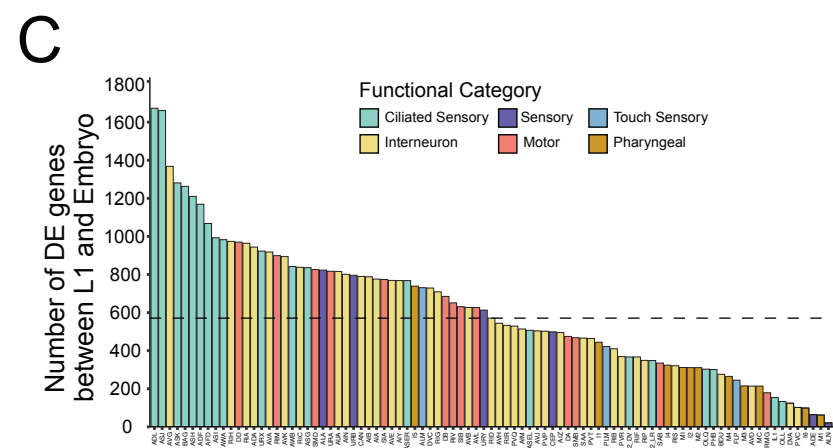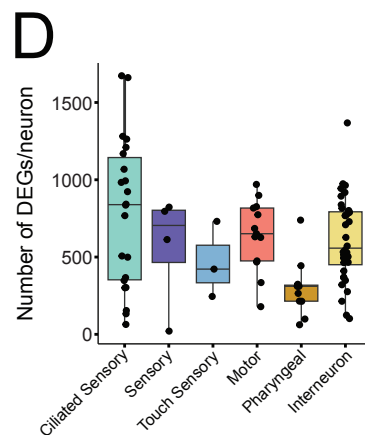

Supplement: Supplement 9 — Supplemental Figure 9. Ciliated neurons share differential expression between the embryo and L1. A) Heatmap as in Figure 5A showing genes (columns) differentially expressed across the nervous system (neurons on y-axis). Neuron categories are depicted by the color scale on the left. Gene sets used for gene set enrichment analysis are depicted by the color bars on the bottom and labeled on the right. B) Wormcat enrichment analysis for five gene sets that showed clustered differential expression (labeled on right, corresponding to labels in panel A). Wormcat gene annotations are shown on the x-axis. The color scale represents fold enrichment over background, and categories with red borders were significant. Fisher’s Exact Test with Bonferroni-corrected p-values. Ciliated neurons shared patterns of higher expression in the embryo of genes (dark blue bars) related to cilia formation. C) Bar graph showing the number of DEGs (Differentially Expressed Genes) per neuron class in the embryo vs L1 comparison, colored by functional category. The horizontal dashed line marks the median of 571 DEGs/neuron. D) Box and jitterplot showing the number of DEGs per neuron class in the embryo vs L1 comparison grouped by functional category. [file media-9.pdf]

A

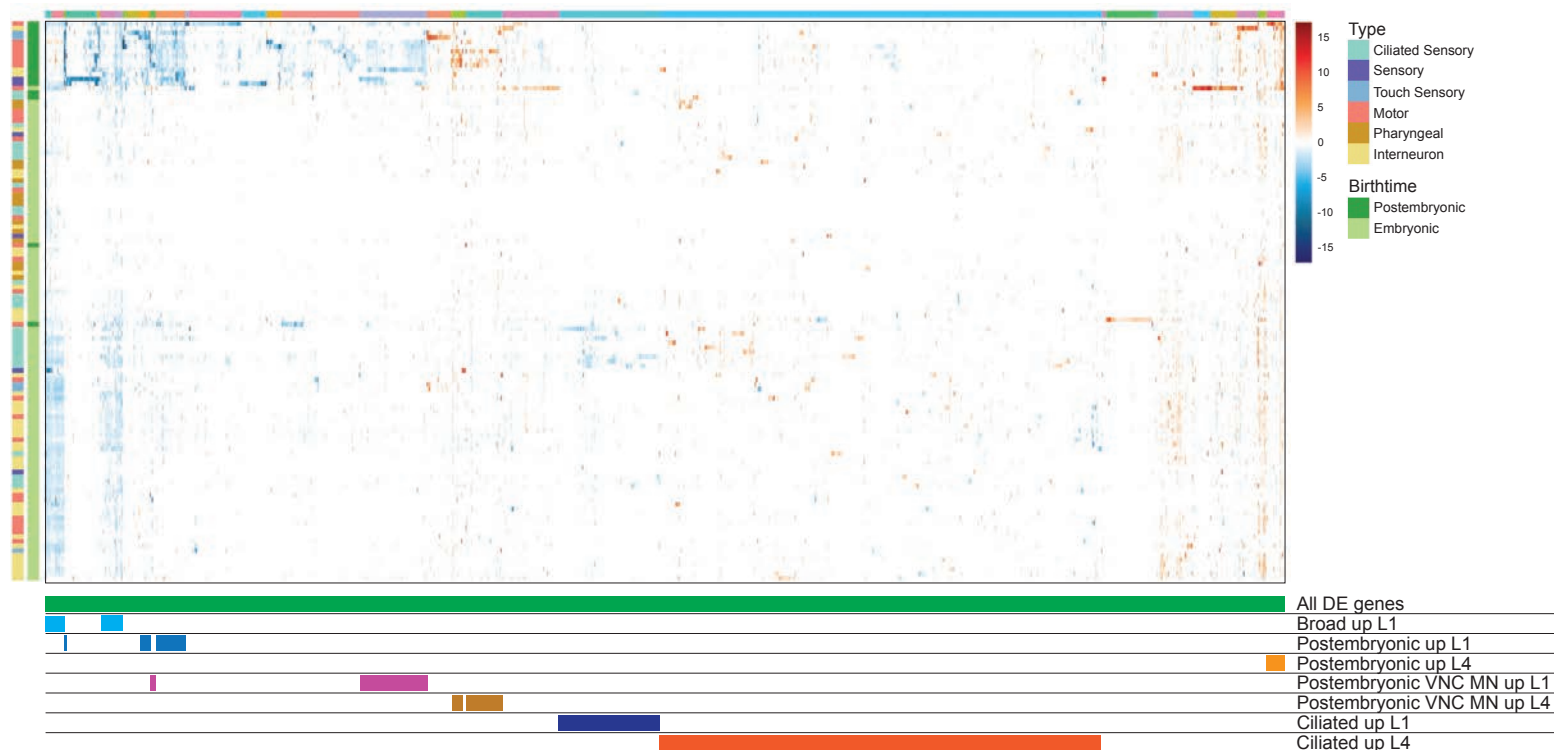

B

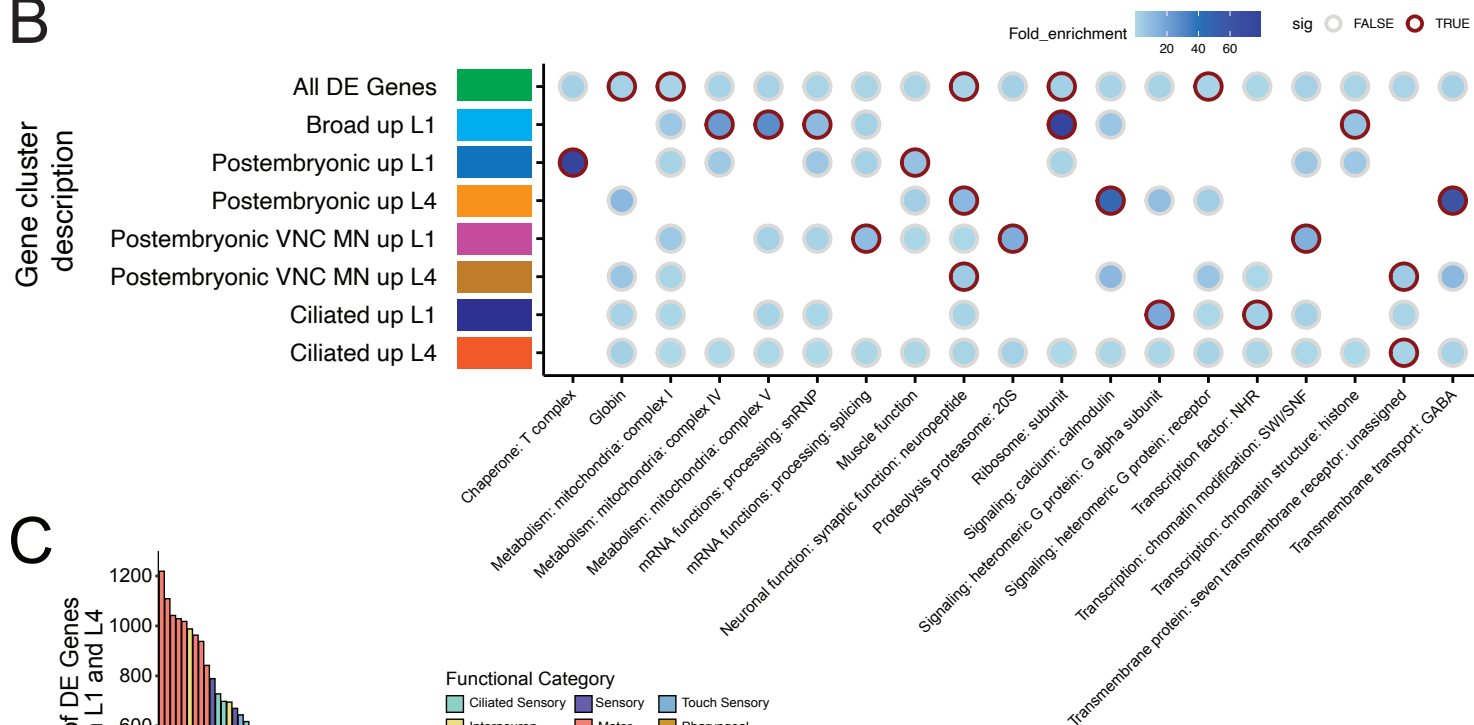

C

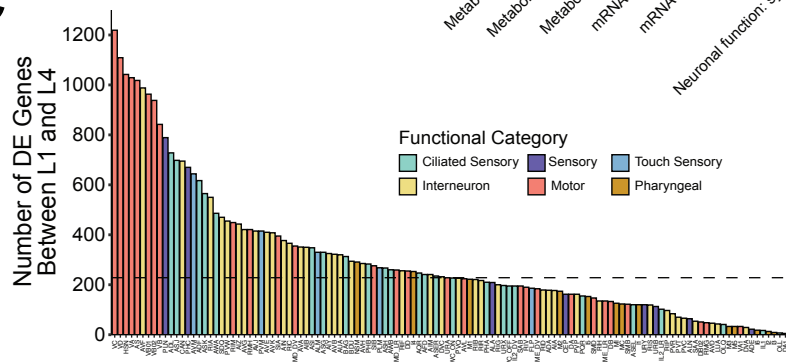

E

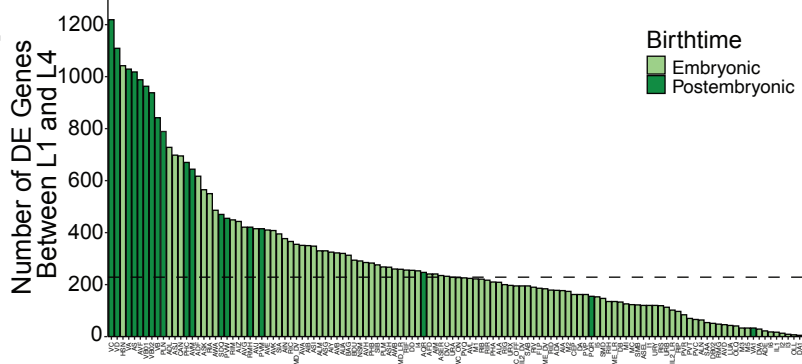

D

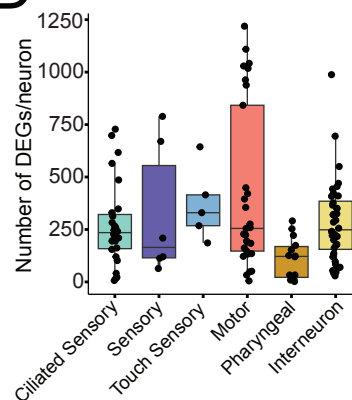

F

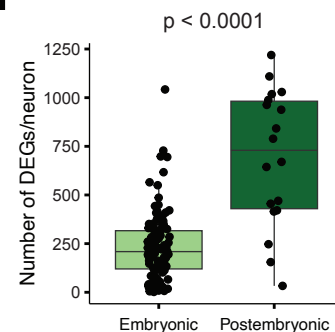

Supplement: Supplement 10 — Supplemental Figure 10. A) Heatmap as in Figure 5B showing differential gene expression between L1 and L4. Columns are genes, rows are neurons. Color scales on y-axis (left) denote functional groups and birthtimes. Hierarchical clustering grouped genes with similar patterns together. Color bars across columns indicate gene clusters. Colored bars below heatmap indicate gene sets that were used as input to Wormcat for gene set enrichment analysis, based on shared pattens of differential expression in multiple related neuron classes, as indicated by labels on the right. B) Gene set enrichment analysis for all DE genes (green, top row), or subsets of genes with shared differential expression across groups of neurons. Wormcat annotation categories are shown on the x-axis. Color scale reflects fold enrichment of genes in each category in the queried gene set over background (all genes detected by scRNA-seq). Dots with red borders were significantly enriched (Bonferroni-corrected p-value < 0.05). Significance was tested with Fisher’s Exact Test followed by Bonferroni correction for multiple comparisons. C) Bar graph showing the number of DEGs/neuron class for the L1 vs L4 comparison, with neurons colored by functional category. The median of 227 DEGs/neuron is depicted by the horizontal dashed line. D) Box and jitterplot of the number of DEGs/neuron grouped by functional category. E) Bar graph of DEGs/neuron for L1 vs L4 as in C but colored by birthtime (embryonic vs postembryonic). F) Box and jitterplot showing the number of DEGs/neuron grouped by birthtime. Postembryonically-derived neurons exhibited more DEGs/neuron than embryonically derived neurons. Linear regression with pairwise comparisons of estimated marginal means and Tukey’s method for multiple comparisons. [file media-10.pdf]

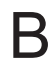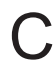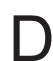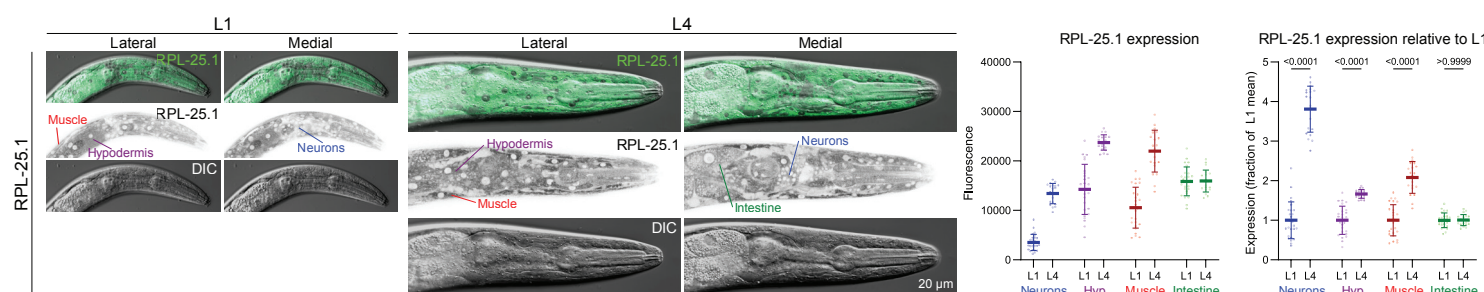

Supplement: Supplement 11 — Supplemental Figure 11. A) Heatmap displaying the log2 fold change between L1 and L4 for ribosomal protein encoding genes (columns) with significant differential expression. Positive log2 fold change values (orange, red) reflect higher expression in L4, negative values (blue) reflect higher expression in the L1 stage. Color scales on y-axis (left) denote functional groups and birthtimes. B) Confocal micrographs showing RPS-4::GFP expression in hypodermis, muscle, intestine and neurons in the anterior regions of L1 (left) and L4 (center) larvae. Quantification (right) of fluorescence intensity shows a reduction in intensity among neurons (p = 0.0504) and intestine between L1 and L4 stages. C) Confocal micrographs of RPL-7A::GFP expression in anterior regions of L1 (left) and L4 (center). Quantification (right) of fluorescence intensity shows decreased expression in neurons, hypodermis and intestine, with no change in muscle, from L1 to L4. D) Confocal micrographs showing RPL-25.1::GFP expression in hypodermis, muscle, intestine and neurons in the anterior regions of L1 (left) and L4 (center) larvae. Quantification (right) of fluorescence intensity shows significant increases in RPL-25.1 levels in neurons, muscle and hypodermis, but not intestine, between the L1 and L4 stages. 2-way ANOVA with Sidak’s correction. [file media-11.pdf]

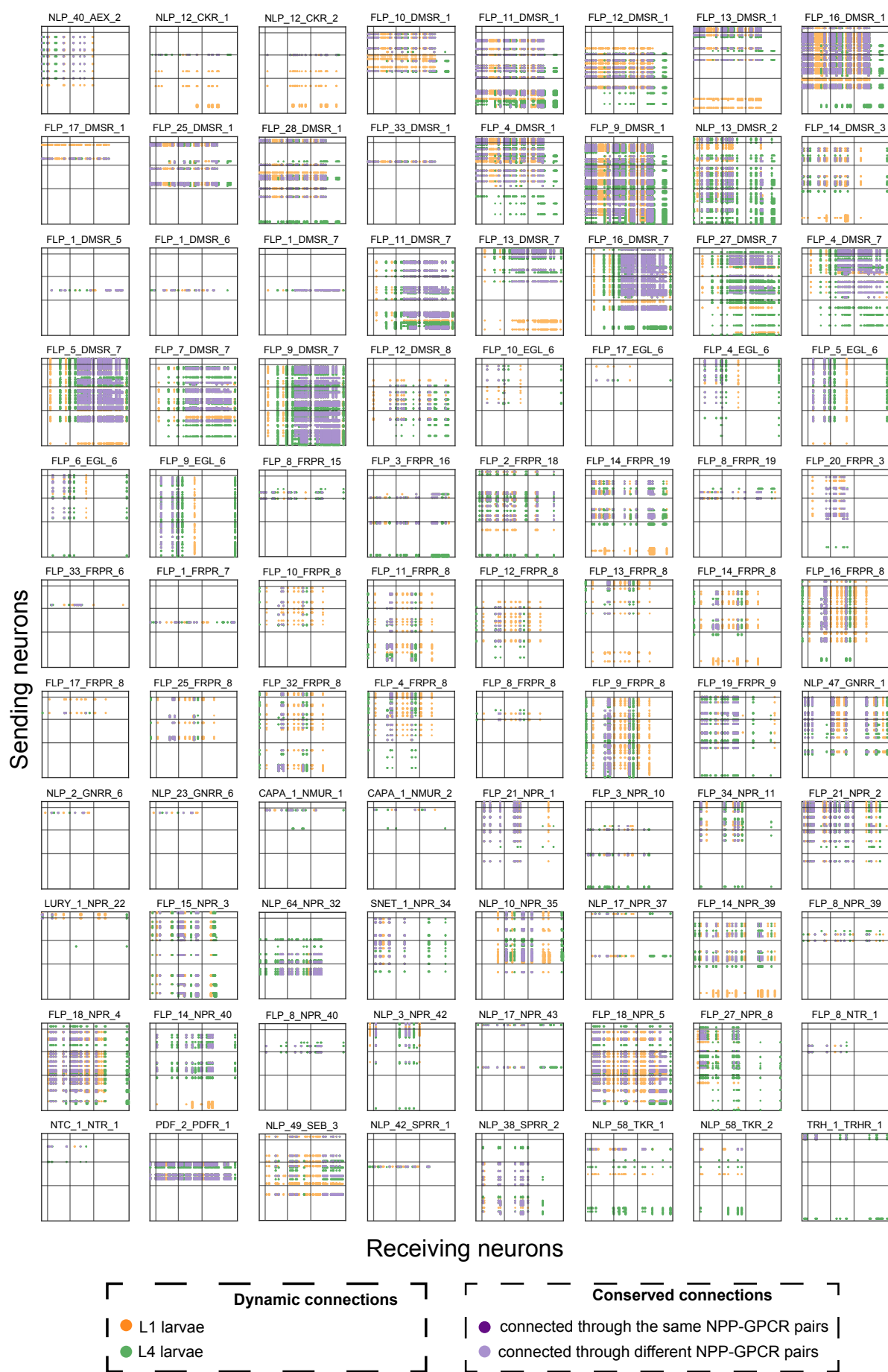

Supplement: Supplement 13 — Supplemental Figure 13. Thresholded neuropeptidergic networks (mid-range) of the 88 NPP-GPCR pairs conserved between L1 and L4 larval stages. The adjacency matrices display the developmental pattern of connections between sending neurons (y-axis) and receiving neurons (x-axis). Columns and rows are sorted by neuron class as in Figure 6 panel A. Core (conserved) connections and developmentally-dynamic connections are color-coded. [file media-13.pdf]

A

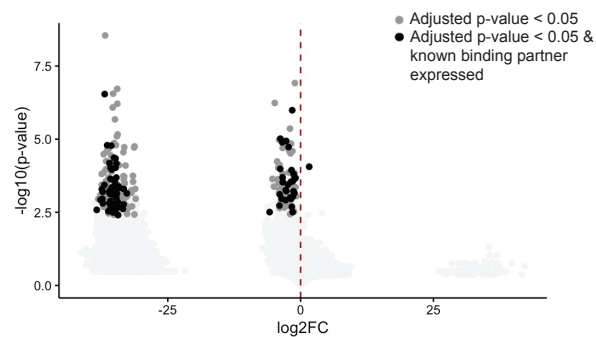

B

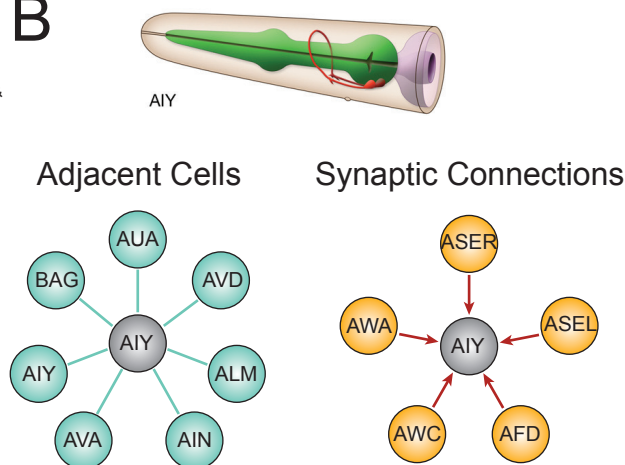

C

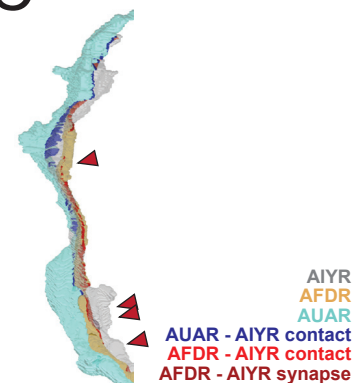

D

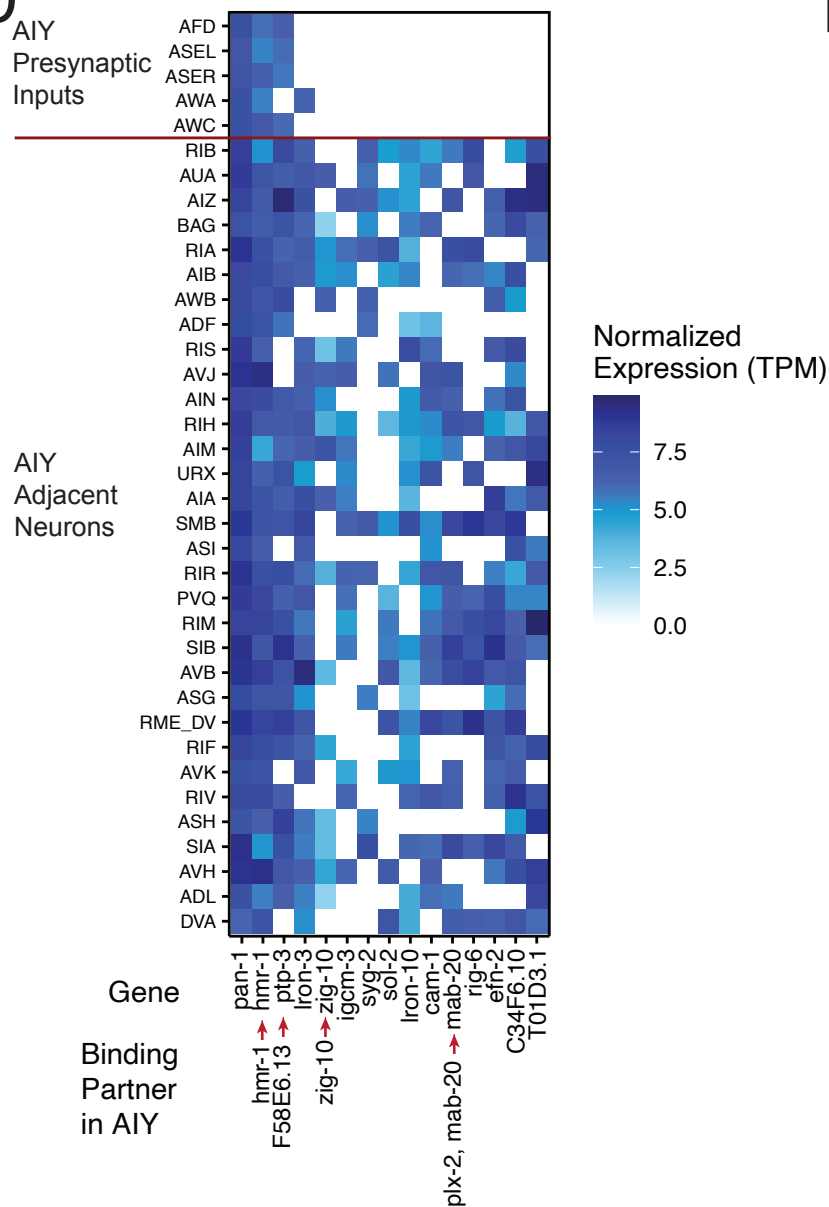

E

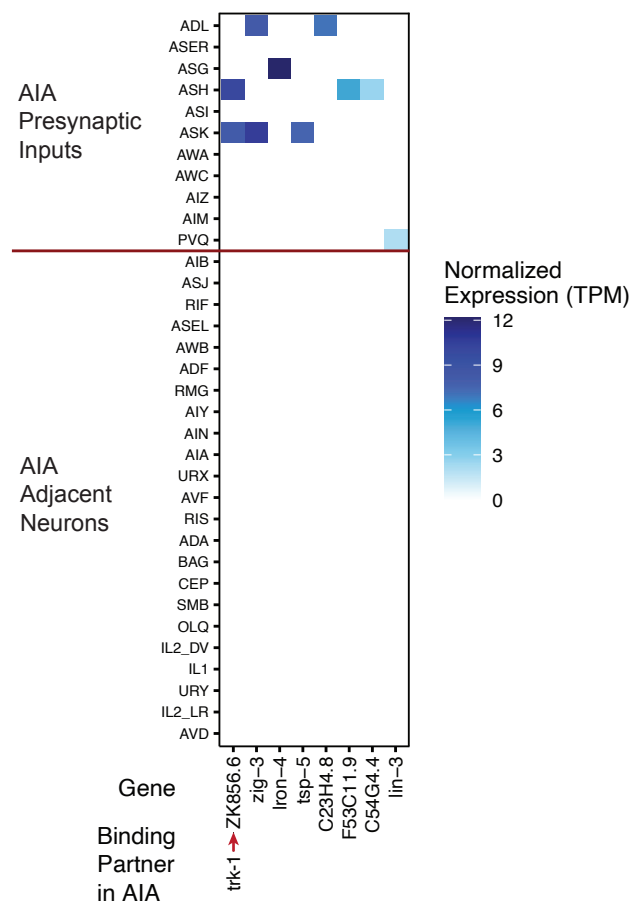

F

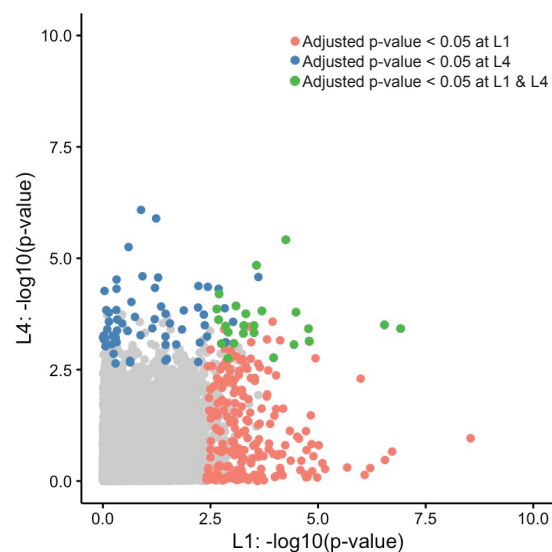

Supplement: Supplement 15 — Supplemental Figure 15. A) Volcano plot showing enrichment of CAMs based on synaptic connectivity. Each dot represents a comparison of one gene tested in the synaptically-connected vs adjacent only neurons for a given neuron. Negative log fold change values represent enrichment in non-synaptic adjacent cells, whereas positive log fold change represent enrichment in synaptically-connected cells. Light gray dots represent instances with Benjamini-Hochberg adjusted p-values > 0.05. Dark gray dots represent cases with adjusted p-values < 0.05. Black dots represent cases with adjusted p-values < 0.05 and in which the tested gene has a known binding partner expressed in the neuron of interest. B) Top: Schematic representation of the AIY neurons, from WormAtlas. Bottom: Adjacent neurons with only membrane contact (left) or synaptic inputs (right) with AIY. Only a subset of adjacent neurons with membrane contact are shown. C) 3D reconstruction from NeuroScan of late L1 nerve ring featuring AIYR, AUAR and AFDR. Both AUAR (light blue) and AFDR (orange) contact AIYR (dark blue and red, respectively), but only AFDR is presynaptic to AIYR (red triangle). D) Heatmap showing the log fold change of 15 CAMs enriched in AIY + non-synaptic adjacent cells (below red line) compared to AIY + presynaptic inputs (above red line). Genes are sorted by log fold change (lowest fold change on left). Genes with known binding partners expressed in AIY are denoted with red arrows, and the respective binding partners are listed below. E) Heatmap showing several genes with large fold changes between AIA synaptically connected neurons and membrane adjacent-only neurons. These eight genes are expressed in only a subset of AIA presynaptic inputs and therefore have adjusted p-values > 0.05. They may, however, still regulate specific individual connections. F) Scatterplot showing -log10 transformed p-values for each gene-neuron combination in L1 (x-axis) and in L4 (y-axis). Genes only detected at one age [file media-15.pdf]
