# Supplementary material for: A gene expression atlas of a juvenile nervous system
Source: bioRxiv. 2025 Nov 22:2025.11.21.689793. Preprint. [Version 1] doi: 10.1101/2025.11.21.689793 (PMC12667811; doi:10.1101/2025.11.21.689793)
Supplement: Supplement 12 — Supplemental Figure 12. Morphological description of neurons at the end of the first larval stage in C. elegans. A-B) Morphological descriptions of neurons born during the first developmental stage (L1) are interpreted from annotated EM images of C. elegans 16h after hatching6. Shown are EM images of the last annotation for each neuron, representing the end of the neuron at this developmental stage. Neuron morphologies are compared to the fully matured adult stage descriptions published in the Mind of the Worm26 (MoW). Panel A features neurons that are fully matured at the end of the L1 larval stage. Panel B features neurons whose morphology is not fully matured at this stage. C) Table adapted from20. Neurons born and removed during each developmental stage, transdifferentiation events and differentiation/maturation of hermaphrodite neurons are listed. [file media-12.pdf]

A

Supplemental Figure 12

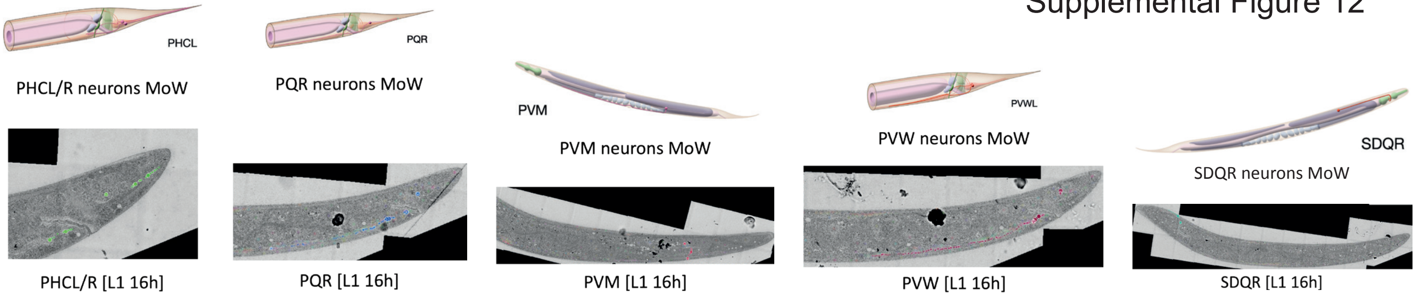

B

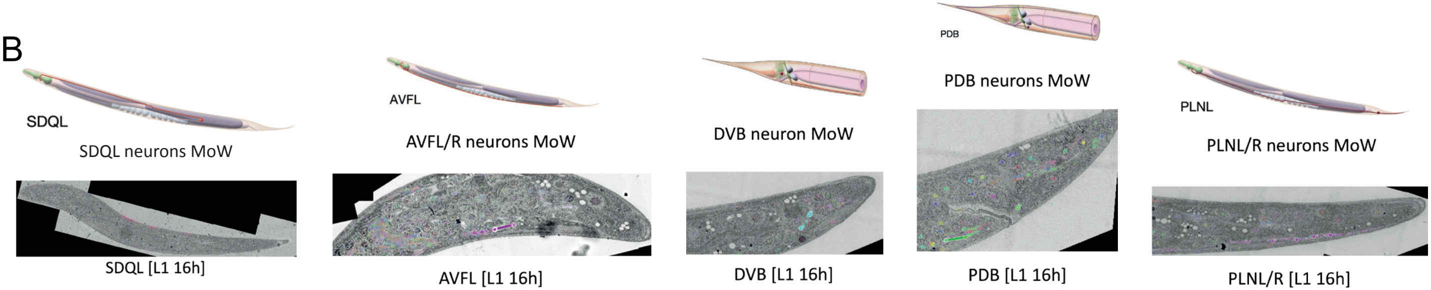

C

	Embryo	L1	L2	L3	L4	Adult
Sex-shared neurons added		AQR PQR<br>AS PVM<br>AVF PVW<br>AVM RMH<br>DVB SDQ<br>PDB VA<br>PHC VB<br>PLN VD	PDE<br>PVD<br>PVN<br>RMF	PDA (transdifferentiation)		
Hermaphrodite specific neurons added/removed	CEMD/V<br>Cell death	VC			HSN and VC terminal differentiation/ maduration	
