# Supplementary material for: A gene expression atlas of a juvenile nervous system
Source: bioRxiv. 2025 Nov 22:2025.11.21.689793. Preprint. [Version 1] doi: 10.1101/2025.11.21.689793 (PMC12667811; doi:10.1101/2025.11.21.689793)
Supplement: Supplement 14 — Supplemental Figure 14. (A) Degree distributions of C. elegans L1 neural networks. In each case, degree (incoming plus outgoing connections) is shown in green, in-degree (incoming connections) in blue and out-degree (outgoing connections) in yellow. The 10 highest-degree hubs in each network are indicated. (B) Degree distributions of C. elegans L4 neural networks, displayed in the same way as panel A. (C) Top 20 neurons that show convergence (similar number of connections, Knorm) or divergence (different number of connections, Kstd) between L1 and L4 larval stages. [file media-14.pdf]

Supp. Fig 14

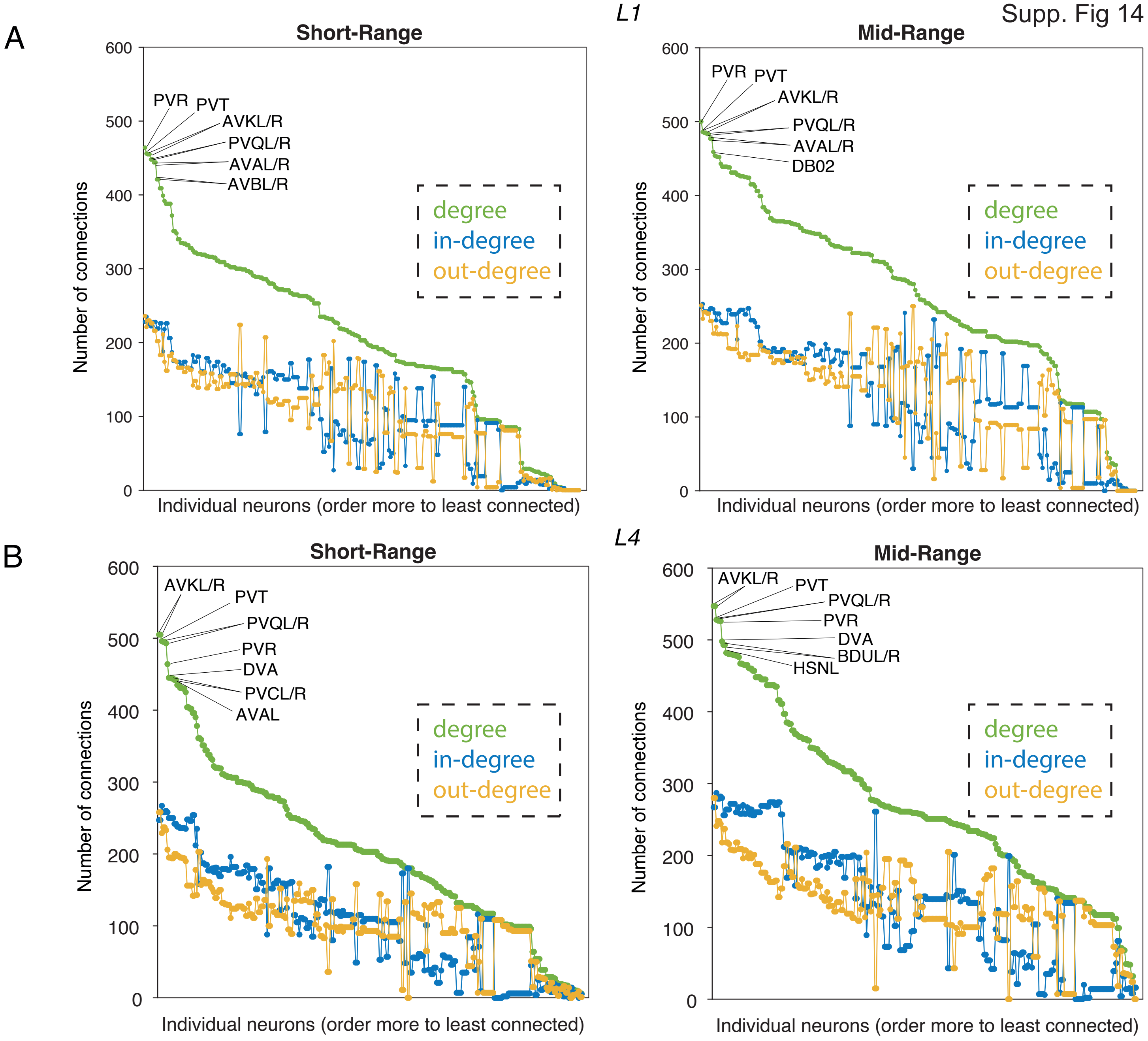

C

Convergent

Neuron	Knorm	Kstd	L1	L4
PVR	1	0	466	476
PVT	0.957739019	0.005914618	458	464
PVQL	0.938955347	0.004720072	450	463
PVQR	0.938955347	0.004720072	450	463
AVKL	0.916003105	0.032982389	457	445
AVKR	0.916003105	0.032982389	457	445
AVAL	0.869518765	0.034344818	446	433
AVAR	0.869518765	0.034344818	446	433
DVA	0.801926765	0.050487334	401	444
AVBL	0.797639485	0.020507453	423	419
AVBR	0.797639485	0.020507453	423	419
AVEL	0.728289654	0.023548703	390	415
AVER	0.728289654	0.023548703	390	415
PVPL	0.720591727	0.045928687	411	390
PVPR	0.720591727	0.045928687	411	390
AVDL	0.681992512	0.015567843	390	389
AVDR	0.681992512	0.015567843	390	389
RMEV	0.651209935	0.053231562	394	368
RID	0.642060086	0.001820876	374	382
PVCL	0.579399142	0.181177145	300	429

Divergent

Neuron	Knorm	Kstd	L1	L4
HSNL	0.166487079	0.507404484	86	430
HSNR	0.166487079	0.507404484	86	430
AVM	0.248365446	0.41351961	133	415
SDQL	0.004858004	0.394122762	4	272
VC01	0	0.34151753	0	233
VC02	0	0.34151753	0	233
OLQDL	0.166560131	0.280750642	304	126
OLQDR	0.166560131	0.280750642	304	126
OLQVL	0.166560131	0.280750642	304	126
OLQVR	0.166560131	0.280750642	304	126
PVM	0.004132043	0.264724352	5	187
IL1DL	0.118573646	0.254670793	265	104
IL1DR	0.118573646	0.254670793	265	104
IL1L	0.118573646	0.254670793	265	104
IL1R	0.118573646	0.254670793	265	104
IL1VL	0.118573646	0.254670793	265	104
IL1VR	0.118573646	0.254670793	265	104
CEPDL	0.104784951	0.251532688	255	96
CEPDR	0.104784951	0.251532688	255	96
CEPVL	0.104784951	0.251532688	255	96
